# Supplementary material for: The genomic history of the indigenous people of the Canary Islands
Source: Nat Commun. 2023 Aug 15;14:4641. doi: 10.1038/s41467-023-40198-w (PMC10427657; doi:10.1038/s41467-023-40198-w)
Supplement: Supplementary file 1 — Supplementary Information [file 41467_2023_40198_MOESM1_ESM.pdf]

Supplementary Information for
The genomic history of the indigenous people of the Canary Islands

Supplementary Information Guide

Supplementary Note 1: Archaeological background 3

Supplementary Note 2: Endogenous content and enrichment results 9
- Shotgun sequencing 9
- WISC capture 9
- MEGA capture 9

Supplementary Note 3: Authentication criteria 11

Supplementary Note 4: Y-chromosome analysis 12
- Molecular sex identification 12
- Y-chromosome haplogroup classification 12

Supplementary Note 5: Ancestry Inference 16
- Datasets 16
- Principal component analysis 16
- Global ancestry 19

Supplementary Note 6: f-statistic analyses 21

Supplementary Note 7: qpAdm modelling 22

Supplementary Note 8: Heterozygosity, inbreeding and family relationships 25
- Family relationship estimations 25
- Heterozygosity estimations 25
- Inbreeding estimations 26

Supplementary Note 9: Effective population size and founder effects 27
- Effective population size 27
- Founder effects 27

Supplementary Note 10: Phenotype analyses 30

Supplementary Note 11: Admixture proportions for the modern Canarian population 31

Supplementary Note 12: IBD analyses 33

Supplementary Note 1: Archaeological background

Angostura (Tenerife)

The archaeological site of Angostura is located in the volcanic caldera of Las Cañadas at El Teide National Park at 2,050 meters above sea level. The site of Angostura is a small cave facing the Northwest (NW). This cave was accidentally discovered in 1982 and the National Park staff members recovered several human remains from the site[1]. Anthropological studies have allowed archaeologists to identify a minimum number of eight individuals[1]. Four of them were young adults, according to the diagnostic traits on their mandible. The preservation of the human remains was relatively poor due to weathering. The bodies probably were wrapped as funerary shrouds made in leather were present on the site. The human remains were associated with other materials such as retama and pine branches, and also remains from lizards (from the now-extinct *Gallotia goliath*) and fragments from one ceramic vessel[1]. One individual from Angostura (Ang-5) was dated between 1318 – 1394 AD (University of Georgia, Athens) by Arnay and colleagues[2]. In this study, we dated two additional individuals, yielding dates between 1396 – 1447 cal AD (CAN.039) and 1276 – 1389 cal AD (95% probability) (CAN.040).

Antoncojo (La Gomera)

The cave site of Antoncojo is located in the middle slopes of the southern side of La Gomera. These human remains were accidentally found in the late 1970s by local villagers. The Antoncojo material was firstly studied by the paleontologist Francisco García-Talavera Casañas in the 1980s (unpublished). The remains consist of several cranial and postcranial remains belonging to one male individual. The radiocarbon dating of this individual produced a chronology between 774 – 994 cal AD (95% probability) (CAN.027).

Arenas-1 (Tenerife)

The Arenas burial site is part of a larger archaeological complex in Buenavista del Norte on the northwest coast of Tenerife. This site was excavated in the late 1990s[3]. The Arenas complex comprises at least three areas that include both burial and domestic sites. Archaeologists recovered remains from a minimum of nineteen individuals in the burial cave of Arenas-1[4]. This cave is a collective burial site where individuals were deposited through time. Primary and disturbed primary burials were recorded in the cave due to its continuous funerary use during a prolonged period. Furthermore, anthropological analyses have indicated a high frequency of certain non-metric traits which has been interpreted as evidence of consanguineal relationships among the individuals of Arenas-1. The present study includes a tibiae fragment from Arenas-1 that produced a radiocarbon date between 1228 – 1297 cal AD (95% probability) (CAN.041).

Gerían (La Gomera)

Gerían is a burial cave located in the Argaga ravine on the southwestern side of La Gomera. This area is well-known due to the high concentration of archaeological sites from the indigenous period, including cave dwellings, burial caves, rock art and ceremonial sites. It is noteworthy that this ravine has evidence of a long-term occupation from the 5th century AD to the 14th century AD[5]. The anthropological analysis of the human remains considered in this study indicated they belong to a female individual. The radiocarbon data obtained from this individual yielded a chronology between 1160 – 1264 cal AD (95% probability) (CAN.031).

Barranco Majona (La Gomera)

This burial site is located in a volcanic tube in the ravine of Barranco Majona in the northern region of La Gomera. The analysis of the human remains from Barranco Majona yielded a minimum number of two individuals (one male and one female), both included in this study. Radiocarbon analysis confirms that both belonged to the indigenous period: 1399 – 1446 cal AD (95% probability) (CAN.028) and 1288 – 1396 cal AD (95% probability) (CAN.029).

Cascajo (Tenerife)

Cascajo is a funerary cave in the volcanic caldera of Las Cañadas at the El Teide National Park. The cave is opened at the northern base of Cascajo Mountain. This burial site is part of a complex consisting of several dwelling caves, one of them with evidence of an extensive occupation[6]. The human remains belong to a non-adult individual treated using natural unguents to preserve its soft tissues. The upper part of the body had a shroud made from goat leather that maintained its anatomical connection and preserved most of the soft tissue[6]. The radiocarbon dates for these remains indicated that this individual lived in the 15th century AD between 1400 – 1450 cal AD (95% probability) (CAN.042)[7].

Cendro (Gran Canaria)

The site of Cendro is a settlement of artificial caves and stone houses on the Telde ravine's left side at the eastern side of Gran Canaria. Cendro and the nearby site of Tara are considered the most populated sites of Telde in indigenous times. In fact, this area played a significant role in the political administration and economy of the indigenous population based on the information provided by European chronicles[8]. Cendro has been systematically excavated in several archaeological seasons since the 1980s. The human remains appeared commingled with young animal remains and other archaeological items in a collapsed artificial cave. The human remains consist of several perinatal remains found in primary position but also disturbed by pillage. Some scholars have proposed that the presence of perinatal remains in Cendro could be related to the practice of infanticide mentioned in some ethnohistorical sources[9]. However, recent studies have provided an alternative hypothesis through genetic and anthropological analyses pointing to high mortality rates and specific burial practices for the perinatal individuals of Cendro[10]. This previous study included the genetic analysis of four individuals dated between the 11th and 13th centuries cal AD (95% probability) (CAN.049, CAN.050, CAN.051, CAN.052). This site has also provided additional radiocarbon dates on charred barley seeds between the 10th and 13th centuries cal AD (95% probability)[11].

Cuermeja (Gran Canaria)

The Cuermeja site is located in the La Aldea area on the western side of Gran Canaria. La Aldea is a wide valley connecting the mountainous inland with the island's western coast. Archaeological evidence highlights that this site was intensively occupied during the indigenous period. Cuermeja site includes dry-stone dwellings and burials on tumuli and cysts. In this study, we analyze the human remains of a single and primary burial in a cyst excavated in the 1980s by the archaeologists of El Museo Canario[12]. The human bones belong to a woman of 17 – 25 years of age. Radiocarbon dates obtained for this individual are between 1270 – 1316 cal AD (95% probability) (CAN.020)[12].

El Agujero (Gran Canaria)

El Agujero is located on the northwestern coast of Gran Canaria. This site consists of dry-stone dwellings and tumuli sharing the same space. El Agujero site is currently separated into several areas due to urban development: Bocabarranco, El Agujero and La Guancha. However,

these sites were part of a continuous burial space during the indigenous period. The most prominent burial is known as the tumulus of La Guancha. This large circular stone monument contains 42 burial areas, with a clear hierarchic space organization in concentric rings and radial walls that arrange the graves and cysts around a central burial. Besides the tumulus of La Guancha, there are other minor tumuli, which can be easily recognized by their central turret, where they buried the individuals, surrounded by one or more stone terraces. Previously available radiocarbon dates came from the central and smaller tumuli and gave this site a chronology between the 11th and 15th centuries AD[8,13]. The individual included in this study was excavated in one of the small tumuli (tumulus 6) and produced a radiocarbon date between 1313 – 1424 cal AD (95% probability) (CAN.008).

El Capricho (Tenerife)

El Capricho is a burial cave on the volcanic caldera of Las Cañadas at the El Teide National Park. Archaeologists discovered and excavated the site in the 1980s[14]. El Capricho contained the remains of two individuals placed in parallel on two large wooden planks used as litter. They rested on a floor conditioned with stones and plant elements. Along with the bodies, there was a perforated bead, another fragmented bead, leather from the funerary wraps, and vegetable elements of the litter[14]. The anthropological analysis indicated the remains belonged to a male between 44 – 50 years of age and a female between 40 – 50 years of age[15]. Further anthropological studies on this site include paleoparasitology, paleodiet and taphonomy analyses. The absolute chronology places the female individual around 428 – 601 cal AD (95% probability)[16–18]. In the present study, we include a teeth sample of the male individual, which produced a radiocarbon date between 1316 – 1437 cal AD (95% probability) (CAN.043).

El Hormiguero (Gran Canaria)

This archaeological site is located on the eastern side of the Cabezo small ravine that joins the Azuaje ravine just before its end at the northern plateau of the island of Gran Canaria. This area is characterized by impressive burial nuclei such as El Hormiguero, El Cabezo and El Barranquillo del Cabezo[19]. These necropolises are collective deposits that take advantage of the natural caves and overlaps of the cliffs that, with small transformations, allowed the indigenous people to use them as burial deposits that were later closed from the exterior with stone walls. One of the burial deposits was studied during an archaeological intervention in 2005. The body placed in this space was an adult male. However, two newborns were also placed on this site after the first burial. Two individuals from El Hormiguero were radiocarbon dated. One produced a date around 790 – 900 cal AD (95% probability) and the other one around 1170 – 1280 cal AD (95% probability)[8,20]. In this study, we include four individuals from El Homiguero necropolis, three dated around the 12th – 16th centuries (CAN.009; CAN.011; CAN.012) and one around the 3rd to 6th centuries cal AD (CAN.010).

El Huriamen (Fuerteventura)

This burial site is located in the volcanic tube of Huriamen on the western side of Fuerteventura. The Huriamen site was accidentally discovered by local villagers and later excavated by archaeologists[21]. Archaeological work on the cave determined the presence of bone remains from at least three individuals. They were two males and one female placed in primary position. The female was between 30 – 35 years of age, and the one male with a preserved skull was between 25 and 30 years of age. Human remains were radiocarbon dated around the 11th – 12th centuries AD. One teeth sample from the female individual is included in the present study with a radiocarbon date between 1041 to 1213 cal AD (95% probability) (CAN.006).

El Portillo (Tenerife)

The site of El Portillo is located in the northern area of the volcanic caldera of Las Cañadas at the El Teide National Park. This cave was discovered in the mid-20th century by local researchers. The cave was initially closed by big stone blocks at the time of the discovery. Behind the blocks, they found six big wood boards, partially burnt, placed vertically, closing the entrance to the funerary space. Behind the logs, it was found the access to the funerary space. In 1980 an archaeological intervention was undertaken. A total of four individuals were found in a primary position, including two young males, one mature female and one young female. Radiocarbon dates from two of the individuals yielded chronological ranges from 1430 to 1520 cal AD (95% probability) and from 1540 to 1652 cal AD (95% probability)[17,22]. Two individuals from El Portillo are included in the present study. One individual was female and was dated between 1300 – 1418 cal AD (95% probability) (CAN.046), before the European conquest. The other individual is one of the young males and belonged to a later phase, yielding a date around 1444 – 1625 cal AD (95% probability) (CAN.045).

Guayadeque (Gran Canaria)

Guayadeque is a large ravine with several archaeological sites on the southeastern side of Gran Canaria. This site includes natural and artificial caves used for domestic and funerary purposes by the indigenous people[23–26]. Burial caves have been surveyed since the 19th century by local and overseas explorers. Because of that, this area has provided a vast number of human remains that are conserved in both Canarian and European museums. These sites contained hundreds of well-preserved individuals buried over a long period, including mummified human remains[27]. Radiocarbon dates from Guayadeque range from the 3rd to the 15th century cal AD, yielding the oldest evidence of human presence in Gran Canaria (this study). We included four individuals from Guayadeque, and radiocarbon dated them between the 3rd to the 9th centuries cal AD (CAN.014; CAN.017; CAN.018; CAN.013).

Huerto de los Morales (La Palma)

The burial site of Huerto de Los Morales is located on the left margin of the Fernando Porto ravine in Garafía at the northwestern side of La Palma. This site was accidentally discovered in 1988 by locals. The archaeologists found a minimum number of 11 individuals. The Huerto de Los Morales site stands out because it has a significant number of non-adult individuals from perinatal to adolescents. In addition, it is worth mentioning the presence of a small clavicle that could belong to an unborn child in the last semester of pregnancy[28]. A phalanx from El Huerto de Los Morales is included in the present study. The human remains belong to a female that lived around 651 – 775 cal AD (95% probability) (CAN.034).

La Fortaleza (Gran Canaria)

The site of La Fortaleza is a large archaeological complex located on the southeast of Gran Canaria. La Fortaleza comprises three main areas: La Fortaleza Grande, La Fortaleza Chica, and Titana. This region has natural and artificial caves dedicated to domestic and funerary purposes, well-preserved grain storage areas (*silos*), rock engravings and numerous surface structures mainly used as houses. Other circular buildings in the upper part of La Fortaleza Grande have been interpreted as spaces destined for ritual activities. Radiocarbon dates demonstrate this is a long-term settlement occupied from the 5th to the 13th century AD[29]. This study includes a female radiocarbon dated between 432 – 642 cal AD (95% probability) (CAN.019).

Lomo Galeón (Gran Canaria)

Lomo Galeón is a burial site on the southwestern coast of Gran Canaria. It is situated on the left margin of the ravine just above the Bahía of Santa Águeda. The site comprises several sets of gravestone cysts excavated in the 1940s and 1980s[8]. The archaeologists found four individuals, including three men and one woman[30]. The three men presented auricular exostosis, a condition related to swimming in cold/temperate waters[31]. The site has already been dated between 1250 – 1290 cal AD (95% probability) based on the analysis of one of the individuals[32,33]. The four individuals from Lomo Galeón are included in the present study, although paleogenomic analysis point to them as being two males and two females (instead of three males and one female as determined by anthropological analyses). All remains have been consistently dated between the 12th and 14th centuries cal AD (95% probability) (CAN.021, CAN.022, CAN.023, and CAN.024).

Los Pasitos (La Palma)

Los Pasitos is an archaeological complex situated on the eastern side of La Palma comprising open-air and cave dwellings, rock-art stations, and a burial cave. A disturbed funerary deposit was recovered by local archaeologists. This site is located nearby significant archaeological sites such as Roque de Los Guerra and Belmaco. In this study, we include a male individual from the site of Los Pasitos (CAN.035) radiocarbon dated between 1157 – 1264 cal AD (95% probability).

Montaña Mina (Lanzarote)

The collective burial cave of Montaña Mina is located in the central area of Lanzarote on the eastern side of a volcano. Montaña Mina was excavated in 1979 and represents the first indigenous burial in Lanzarote to be systematically excavated[34]. The cave is placed in an area where significant archaeological sites are located, including La Atalaya, Lomo de San Andrés, Zonzamas and Fiquinineo. In this study, we include two male individuals from Montaña Mina. These individuals are radiocarbon dated between 1045 – 1222 cal AD (95% probability) (CAN.037) and 1162 – 1264 cal AD (95% probability) (CAN.038).

Puente de la Calzada (Gran Canaria)

Puente de la Calzada is a burial cave located in the Guiniguada ravine in the central-eastern area of Gran Canaria. The site was discovered in 1993 and excavated in 2002. The cave contained seven individuals that were placed in two different periods. The human remains from the later episode were found in a primary position, while those from the earlier burial were mainly recovered in a secondary position[35]. We included two individuals from Puente de La Calzada, one male and one female, with radiocarbon dates between 1265 – 1388 cal AD (95% probability) (CAN.26), and 1303 – 1410 cal AD (95% probability) (CAN.025), respectively.

Punta Azul (El Hierro)

Punta Azul is a burial site located in a volcanic tube at the cliff of the southwestern coast of El Hierro. The site of Punta Azul is part of a large sepulchral area composed of dispersed natural caves that worked as collective burials. Among them, we can find some of the most important indigenous burial places, such as Montaña de La Lajura, Cueva de la Ballena or Letime[36,37]. The site was discovered in 1947 by the archaeologists Álvarez Delgado and Diego Cuscoy. They identified six individuals in a primary position and other human remains in a secondary/disturbed position. They only recovered those elements that were considered relevant at the time. The recovered materials included five complete and five fragmented skulls, 18 mandibles, ten

femurs, a piece of fur from a mortuary shroud, a lithic instrument, and a goat horn[39]. This site was re-excavated in 1994. They found an intensively disturbed burial where human remains were totally disarticulated in a secondary/disturbed position. More than 6,000 bone specimens were collected, including bones, bone fragments and teeth[40]. Some studies on the Punta Azul site have been focused on performing paleopathological[41–45], paleodietary and paleo-nutritional studies[46–48], as well as the establishment of discriminant functions for the tibia[49], and the analysis of the particular characteristics of this population and its comparison with other contexts[44,50,51].Two radiocarbon dates have already been obtained for two individuals from Punta Azul: 1022 – 1159 cal AD (95% probability) and 1040 – 1214 cal AD (95% probability)[36]. Four individuals from Punta Azul are included in the present study. Two are male and two are female (CAN.001, CAN.002, CAN.003, and CAN.004). Radiocarbon dates were obtained for these individuals yielding a chronology between the 13th and 15th centuries cal AD.

Salitre (Tenerife)

Salitre is a burial site located in a volcanic crevice on the northern side of Montaña Rajada at the volcanic caldera of Las Cañadas (El Teide National Park). The site has been known since the 19th century, but archaeological fieldwork was not performed until 1945. Human remains and fragments of leather shrouds and clothes were recovered from this site[39]. The bodies were in supine decubitus over a two-layered bed mostly made of stone and vegetables. There is evidence of planks and forks used to make floorboards and even shelf systems that could hold standing bodies[39,52]. The human remains show excellent preservation due to the natural presence of natron or *salitre* in the cave's walls. There is an ongoing debate on the minimum number of individuals buried here, ranging from 50 to 300. Archaeological excavations have identified a dozen individuals, male and female, and from all age groups, including children between four and seven years of age[17,53]. There are two already available radiocarbon dates for this site: 1045 – 1282 cal AD (95% probability) and 1517 – 1656 cal AD (95% probability)[2]. This study includes one male radiocarbon dated around 1024 – 1155 cal AD (95% probability) (CAN.048).

Salto Casimiro (La Palma)

The burial site of Salto de Casimiro is located in the Hermosilla ravine in the central western area of La Palma. The site was accidentally discovered in the 1970s during road construction. Unfortunately, these road works destroyed a significant part of the necropolis. However, an archaeological intervention was performed in the preserved area of the site (Nuria Álvarez Rodríguez, *personal communication*). We included a sample from one female radiocarbon dated between 579 to 652 cal AD (95% probability) (CAN.052).

Supplementary Note 2: Endogenous content and enrichment results

Individuals with high endogenous DNA content (>10%) and post-capture libraries were sequenced to saturation on an Illumina NextSeq 500 platform (paired-end reads, 2 x 75 bp and 2 x 42 bp). Mapping and filtering were performed as in[54]. Briefly, reads were trimmed and adapters removed using AdapterRemoval version 1.5.4[55], with a minimum insert size of 30 bp and a minimum base quality of 20. Paired-end reads were then merged with a minimum overlap length of 11 bp. Trimmed merged reads were then mapped to the human reference genome (GRCh37) using BWA version 0.7.12[56]. For libraries sequenced using the 2 x 42 bp paired-end method, unmerged reads up to 141 bp were also kept for analysis, replicating the same insert size as 2 x 75 bp paired-end merged reads. For libraries sequenced using the 2 x 42 bp paired-end method, we also used the clipOverlap function from bamUtil v.1.0.14 to trim overlaps smaller than 11 bp on paired-end reads[57]. The mapping was performed using "bwa -aln" with the seed option (-l) disabled. After mapping, we removed reads with a mapping quality lower than 30, duplicated reads and reads with alternative mapping coordinates. All filtering was performed using SAMtools version 0.1.19[58]. Bam files from different runs were merged per individual using SAMtools merge.

The amount of endogenous DNA for shotgun and capture libraries was calculated by dividing the number of reads after filtering by the total number of trimmed reads. To normalize results, a subset of 2 million raw reads was used for comparison between individuals. All figures were produced using R version 3.6[59] and the package ggplot2[60].

2.1. Shotgun sequencing

Endogenous DNA content was highly variable between and within archaeological sites. DNA conservation was relatively good for some of the indigenous individuals, with six individuals from four different archaeological sites showing rates above 30% (Supplementary Data 1; Supplementary Fig. 1). Overall, endogenous DNA in the shotgun libraries accounted for 10.7 ± 3.8% of the total (median = 4.6%, IQR = 1.5% – 17.7%). Complexity of the libraries was also good, with an average duplicate rate of 0.66 ± 0.12% (Supplementary Data 1; Supplementary Fig. 2).

2.2. WISC capture

WISC capture was applied to 23 shotgun libraries. WISC performance was extremely variable between individuals, with enrichment values ranging from 0.20X to 29.36X (Supplementary Fig. 3; Supplementary Data 1). In general, WISC results were modest, with a mean enrichment value of 6.8 ± 2.9X (median = 3.5X, IQR = 2.2X – 8.7X) and an important decrease of the libraries complexity (Supplementary Fig. 4). The average duplicate rate increase was 57.3 ± 41.8X (median = 29.0X, IQR = 9.5X – 66.3X), with values ranging between 3.9X and 492.7X.

2.3. MEGA capture

Ancient DNA libraries were also captured for SNPs contained in the Illumina Multiethnic Genotyping Array (MEGA) array. MEGA capture produces a relatively good increase of coverage on the targeted SNPs (Supplementary Data 1), with an average increase of intersected

MEGA array SNPs of 81.2 ± 18.5X (median = 60.0X, IQR = 42.1X – 94.6X). Enrichment values ranged from 22.5X in individual CAN.017 to 226X in individual CAN.026. As observed with WISC, the decrease of the libraries' complexity was high. The average duplicate rate increase was 48.94 ± 24.36X (median = 29.59X, IQR = 16.82X – 53.94X), with values ranging between 9.44X and 444.72X.

Supplementary Note 3: Authentication criteria

Authenticity of the data was assessed with MapDamage version 2.0.2[61,62] to identify the presence of damage associated with cytosine deamination and fragmentation, and with ContamMix version 1.0-10[63] and Schmutzi[64] to calculate contamination rates. For contamination analysis, we used the mtDNA bam files filtered using the same pipeline as for nuclear DNA (see Supplementary Note 2), and with 3 bp trimmed at both ends to avoid damage interfering with contamination estimations. Simultaneously, we assessed for the autosomal contamination using the non-pseudoautosomic X-chromosomal data on male individuals. For that purpose, we used ANGSD[65] on X-chromosome polymorphic sites using reads with a base quality >20 and mapping quality >30.

Post-mortem damage patterns were as expected for ancient DNA. Deamination at the 3' ends of reads ranged between 6.12% and 44.14%, with an average value of 18.14 ± 2.68% (Supplementary Data 1; Supplementary Fig. 5). Average insert size was 60.99 ± 3.51 bp, with values ranging between 39.18 and 85.79 bp (Supplementary Data 1; Supplementary Fig. 6).

As published before for most of the individuals of these dataset[66], contamination rates are low accounting for an average value of 1.95% ± 0.54% estimated with ContamMix and 1.52% ± 0.16% with Schmutzi (Supplementary Fig. 7). Most of the human remains also show low levels of autosomal contamination, except for five males who display 6.1% to 15.2% of contaminated reads (Supplementary Data 1). However, as ANGSD contamination estimations on ancient low coverage (<0.1X) data are problematic, we decided to keep the three individuals with autosomal contamination levels >5% and <0.1X for further analysis. Also, as the previously published gun002 and CAN.027 have contamination estimations of 6% and 8.2%, respectively, and their mtDNA contamination estimations are very low, we decided to keep them but considering their status.

Supplementary Note 4: Y-chromosome analysis

4.1. Molecular sex identification

The molecular sex of the individuals was identified using the ry estimate[67] and results were plotted using the R programming language (version 3.6)[59] and the package ggplot2[60]. As observed before[54], we needed to filter pseudo-autosomal and repetitive regions prior to ry calculation to accurately assess the molecular sex of captured individuals (Supplementary Fig. 8). Including previously published genomes from[68,71], 21 individuals are females and 28 are males (Supplementary Data 1, Supplementary Fig. 8).

4.2. Y-chromosome haplogroup classification

Male individuals were subjected to Y-chromosome analysis as in[54]. First, we performed a SNP calling based on the ISOGG Y-DNA Haplogroup tree 2019-2020 database (v.15.73) using samtools mpileup, filtering out bases with BASEQ < 30. The resulting variants were classified in two groups based on the putative presence of DNA damage (C → T or G → A) and were visually classified among the main haplogroups following the Y-chromosome phylogenetic tree[69]. Additionally, individuals were classified using pathPhynder's "best path" method[70]. All figures were produced using R v. 3.6[59] and the R packages ggplot2[60] and ggtree[70].

Including previously published genomes from[68,71], a total of 28 ancient individuals from the Canary Islands were identified as males. Their geographic adscription is as follows: El Hierro (n = 2), Gran Canaria (n = 12), La Gomera (n = 2), La Palma (n = 1), Lanzarote (n = 2) and Tenerife (n = 9). No males were observed for Fuerteventura; therefore, no information is available regarding Y-chromosome lineages present in the ancient population of this island.

Overall, the most frequent Y-chromosome haplogroup in the indigenous population of the Canary Islands is E-M183 (57.1%), followed by T-M184 (21.4%), E-M33 (10.7%) and R-M269 (7.1%). Finally, E-M78 was observed at a frequency of 3.6% (Supplementary Fig. 9; Supplementary Data 2).

Initially, information on the Y-chromosome composition of the indigenous population was obtained using classical methodologies based on PCR[36,72]. Although E-M183 was not genotyped, a SNP for the E-M81 branch was included. As E-M81(xE-M183) is extremely infrequent[73] we can consider all E-M81 males to belong to the E-M183 sublineage. In the 2009 study[72], E-M183 (E-M81) was also the most common Y-chromosome lineage, although in that case the frequency was estimated to be 26.7%. The frequency for E-M33 (10.7%) was also estimated to be lower when using PCR methods (3.3%). On the other hand, the frequency of E-M78 was higher in the previous study (23.3%) than in this NGS analysis (3.6%). Moreover, haplogroups I-M170, J-M267 and P-M45* were observed by Fregel et al.[72] but not in the present study. Additionally, in the 2017 Y-chromosome analysis of the Punta Azul site in El Hierro[36], both E-M183 and E-M269 were found at a frequency of 43.8%, and E-M33 at 6.3%. One possibility for the differences in haplogroup frequencies could be that the multiplex-PCR method used in the 2009 and 2017 studies produced incorrect results due to contamination and/or PCR artifacts. However, for those individuals genotyped using both multiplex-PCR and NGS techniques (Punta Azul site: CAN.001 and CAN.002; Guayadeque site: CAN.013 and CAN.018) results were consistent using both methodologies, indicating that contamination or PCR errors were not prevalent when using the PCR approach. Another possibility can be that these differences could be caused by the

fact that sample sizes for the Y-chromosome are still low and also that most of the people analyzed in the 2009 and 2017 studies came from the Guayadeque site in Gran Canaria and the Punta Azul site in El Hierro, respectively, instead of being a miscellaneous individuals from the entire archipelago. From the mtDNA point of view, it is evident that the populations of the different islands were not homogenous. For that reason, we must consider that the same scenario could be observed for the Y-chromosome. Future paleogenomic efforts would be needed to characterize the Y-chromosome composition of the Canary Islands at an insular level.

4.2.1. Phylogeographic analysis of Y-chromosome haplogroups

4.2.1.1. Haplogroup E-M183

As explained before, most males belonged to the E-M183 haplogroup. Concretely, there are two of them from El Hormiguero (Gran Canaria) and one male from each of the archaeological sites of Punta Azul (El Hierro), Puente de la Calzada (Gran Canaria), Antoncojo (La Gomera), Barranco Majona (La Gomera), Los Pasitos (La Palma), Arenas (Tenerife), El Cascajo (Tenerife), El Capricho (Tenerife), El Portillo (Tenerife) and El Salitre (Tenerife) that belong to E-M183 haplogroup (Supplementary Fig. 9; Supplementary Data 2). Also, three decontextualized Tenerife individuals from[68] belong to this lineage. This haplogroup has not been observed on the island of Lanzarote but given its small sample size (n=2), a larger sample size would be needed to characterize the Y-chromosome composition of this population.

Given that North Africa is its most probable origin, a high frequency of E-M183 in the indigenous population of the Canary Islands is expected. This lineage, derived from E-M81, is the most common haplogroup in modern North African populations and considered to be autochthonous in this region[74,75]. Actually, a lineage ancestral to E-M81 has been observed in early Neolithic people from Morocco dated 7,000 BP[54]. The highest frequencies of E-M81 are observed today in Western Sahara (76.9%), Morocco (62.1%) and certain populations in Algeria (82.1%)[73]. This study has estimated a TMRCA (time to most recent common ancestor) of only 2,000 - 3,000 years for E-M183, placing the origin of this lineage around the time the Canary Islands were colonized. Solé-Morata et *al.* 2017[73] also observed a rapid radiation for E-M183 and identified five different branches within E-M183. We tested our individuals to determine if they belonged to the basal E-M183* lineage or to some of the sister branches proposed by[73]. Our results indicate that all of them belong to E-M183*, which is expected given that its coalescence date coincides with the time estimated for the human colonization of the Canary Islands.

Males belonging to the E-M183 cluster were also assigned to the same haplogroup when analyzed using pathPhynder (E1b1b1b1a1), except for CAN.023 (Lomo Galeón) and CAN.025 (Puente de la Calzada) from Gran Canaria. According to pathPhynder, CAN.023 belonged to the ancestral E-M310 haplogroup (E1b1b1b1), and CAN.025 could not be resolved further than the E haplogroup, probably due to the low coverage of these individuals (<0.1X). Interestingly, E-M183 individuals are placed in the phylogeny near to Mozabite people. Along with them, indigenous individuals are placed near to Puerto Rican and Iberian individuals, who could be descendants of Canarian migrants.

4.2.1.2. Haplogroup E-M33

Three males from the eastern islands belong to haplogroup E-M33 (Supplementary Fig. 9; Supplementary Fig. 10; Supplementary Data 2): one from the site of Lomo Galeón (Gran Canaria) and two from the site of Montaña Mina (Lanzarote). E-M33 is considered to be a sub-

Saharan lineage whose highest frequencies (~50-60%) have been observed in South and Central Africa[74,75]. E-M33 is also present in current populations in North Africa, reaching its peak in West Sahara (9.0%); however no complete sequenced E-M33 Y chromosome is available from this region. Considering E-M33 is a lineage of sub-Saharan origin, the presence of E-M33 in the indigenous population of the Canary Islands confirms the impact of sub-Saharan migrations into North Africa prior to ~2,000 years ago. This result is consistent with sub-Saharan mtDNA lineages such as L1 or L2 observed in the Canarian native people[66]. It is worth mentioning that for the two Canarian individuals (CAN.037 and CAN.038) a fraction of the SNPs covered in the branch leading to E-M33 are ancestral. For CAN.037, only two of the three SNPs covered are derived (67%). For CAN.038, a larger number of SNPs were covered, with 9 out of 15 being derived (60%). Most of the E-M33 males included by[69] in the complete Y-chromosome phylogeny were of sub-Saharan African origin, mainly from Gambia. The results obtained for the Canarian indigenous males indicate that they belong to a different sublineage of E-M33 than that previously observed.

When analyzed using pathPhynder, all the individuals were placed within the E-M33 haplogroup. Furthermore, CAN.022 could be assigned to the E1a1 descendant lineage defined by the marker M44, next to a Mandenka individual.

4.2.1.3. Haplogroup R-M269

Two indigenous individuals belong to the R-M269 haplogroup (Supplementary Fig. 9; Supplementary Data 2): one from Punta Azul (El Hierro) and one from Guayadeque (Gran Canaria). R-M269 is the most common haplogroup in Western Europe, although it is also found in North Africa in lower frequencies[77]. When ancient individuals were further classified within R-M269, both showed derived SNPs on the branch clustering R-L11 individuals. Although the R-L11 lineage is commonly restricted to Western Europe[77], it was common in Early Bronze Age populations from Europe[78] and could have reached North Africa with Bronze Age migrations from this region (as implied by the presence of Bell Beaker pottery[79,80]). Analysis using pathPhynder confirmed previous results as both individuals were placed within the R-L11 branch of the R-M269 haplogroup (R1b1a2a1a).

4.2.1.4. Haplogroup T-M184

Six males from our dataset belong to the T-M184 haplogroup (Supplementary Fig. 9; Supplementary Fig. 11; Supplementary Data 2), all of them from different archaeological sites in Gran Canaria: El Agujero, Cendro, El Hormiguero and Guayadeque. T-M184 originated in the Near East and began to diversify approximately 25,000 years ago and then dispersed to Europe and the sub-Saharan Africa[81]. Today, haplogroup T is widespread, but it is considered to be rare.

As with mtDNA lineages T2 and J2[66], the presence of the Y-chromosome T-M184 haplogroup in the indigenous population of the Canary Islands can be explained by Neolithic expansions in North Africa, as this haplogroup has been found in early farmers from Europe[82]. In fact, this was one of the Neolithic-related lineages directly found in Late Neolithic Moroccan remains (3,700 - 3,600 BCE) from the Kef el Baroud site[54].

When analyzed using pathPhynder, four of the six males were assigned to the same T-M184 haplogroup. However, CAN.008 from Guayadeque could not be resolved further than the IJK haplogroup probably due to the low genome coverage. On the other hand, the Cendro individual (CAN.049) was assigned to the L208 branch of the T-M184 haplogroup that dispersed all over western Eurasia after its appearance in the Near East.

4.2.1.5. Haplogroup E-M78

One individual from La Angostura site in Tenerife belongs to the haplogroup E-M78 (Supplementary Fig. 9; Supplementary Fig. 12; Supplementary Data 2). This haplogroup is a widely distributed lineage that most likely originated in northeastern Africa[74,83,84] and dispersed from there approximately 20,300 - 14,800 years ago. For that matter, Upper Paleolithic people from Morocco dated 15,000 years BP belong to this lineage[85]. The highest frequencies of the haplogroup E-M78 are nowadays observed in southern Egypt (50.6%) and Somalia (52.2%)[83]. Contrary to what happens with E-M81, haplogroup E-M78 shows higher frequencies in eastern North Africa (25.4%) and decreases towards the West (11.6%). It is worth mentioning that, according to the data in[83] E-M78 is more frequent among Moroccan Arabs (40.0%) than in northwestern Berber populations (e.g. 3.7% in Asni, 1.5% in Bouhria or 0.0% in Mozabite Berbers). When analyzed using pathPhynder, this individual could not be resolved further than the parent E-M215 clade. However, our analysis only identified only one read covering the E-M78 mutation so probably the lack of coverage affected the pathPhynder results.

Supplementary Note 5: Ancestry inference

5.1. Datasets

The Canary Islands ancient dataset was first compared to the Human Genome Diversity Project (HGDP)[86], genotyped on Illumina, Inc.'s Multiethnic Genotyping Array (MEGA), curated as published before[54] and including available North African, sub-Saharan African, Near Eastern and European populations. Our dataset was also compared to the Human Origins panel, which contains 2,345 present-day humans from 203 populations and 281 ancient individuals genotyped for 594,924 SNPs[87]. Ancient DNA shotgun data from previously published data was also integrated into the Human Origins aDNA dataset, including Neolithic and Bronze Age individuals from Spain[88,89], Turkey[90], Ireland[91], Iran[92], and Morocco[54], as well as from the indigenous population of the Canary Islands[68,72], the Iberomaurusian site of Taforalt[85], ancient Egypt[93] and several archaeological sites from sub-Saharan Africa[94,95]. We also included published Punic[96] and Roman[97] individuals as they have been reported to carry North African ancestry and their comparison with the indigenous people of the Canary Islands could be of interest for determining historical contacts. Lastly, we filtered the Human Origins dataset in order to obtain a sub-dataset including both ancient and modern populations from Eurasia (including North Africa) and sub-Saharan Africa.

5.2. Principal component analysis

5.2.1. MEGA-HGDP panel

Ancestry of the indigenous people from the Canary Islands was first inferred from PCA using the MEGA-HGDP reference panel. PCAs were performed both using both LASER[98,99] (Supplementary Fig. 13) and the lsqproject option from smartpca[100] (Supplementary Fig. 14). LASER coordinates were calculated as the average of 10 replicates. The first component in this PCA separates sub-Saharan African from Eurasian populations, while the second one separates European from North African and Middle Eastern populations.

Both the LASER and the lsqproject PCA plot show that the indigenous people of the Canary Islands are clustered near to Late Neolithic Moroccans and modern North Africans. A previous study determined that Upper Paleolithic and Early Neolithic people from Morocco shared the same genetic composition, associated with what is known as the autochthonous Maghrebi component[101], while Late Neolithic Moroccans showed admixture with Anatolian and/or European early farmers[54]. The indigenous people of the Canary Islands are also placed in an intermediate position between the ancient North Africans and Sardinians (characterized by their higher European Neolithic component), and close to Late Neolithic Moroccans as previously noted[64,54,68,102]. As expected from the presence of sub-Saharan African mtDNA and Y-chromosome lineages in the indigenous individuals from the Canary Islands[66], both the LASER and lsqproject PCAs indicate that the Canarian natives are closer to sub-Saharan African populations in PC1 than Late Neolithic Moroccans. However, it could also be due to other additional migration sources from Eurasia. Also, with our increased sample size, we observe that the indigenous population is placed more scattered compared to late farmers in Morocco, with some individuals showing a higher affinity for ancient North Africans and other individuals clustered closer to Sardinians.

MtDNA evidence suggests that eastern and western islands could have a different genetic composition[66], which could account for this variation in the placement of ancient people from the Canary Islands. To test that hypothesis, we plotted the indigenous people using a different color code for each island and we observe that western islands are clustered closer to Upper Paleolithic and Early Neolithic North Africans, while eastern islands are placed closer to Sardinians and other modern European populations (Supplementary Fig. 15). There is only one exception to the eastern/western distribution: one individual from Tenerife (CAN.039) that is placed within the eastern islands' cluster. Some of the archaeological sites from Tenerife we included in this study belong to the post-contact period. Individual CAN.039 belong to the archaeological site of La Angostura, a site with clear indigenous funerary practices and material culture[17]. Radiocarbon dating from this site indicates this burial was used between 1,295 and 1,414 AD[17]. After performing RCD on this particular individual, results indicate that CAN.039 was dated between 1,396 – 1,447 cal AD. As contact with European sailors and explorers started in Tenerife in the 13th century, it leaves the possibility of European admixture or that this individual came from a different island.

Although aDNA libraries have been subjected to enrichment techniques, a limitation of our study is the low coverage of some individuals (Supplementary Data 1), which makes us question if sample clustering in the PCA can be accurate. The advantage of using LASER is that sequence data for each reference individual is simulated to match the coverage pattern of each individual. Then a PCA ancestry map is built based on the simulated sequence reads for both the reference and the ancient individuals. Finally, the ancestry map is projected into the PCA space built based on the whole reference panel. The stochastic variation introduced by the simulation procedure of the LASER method can lead to slightly different results in the placement of each individual. For that reason, our analysis was based on the average of 10 independent replicates. This approach allows us to better place each individual into the PC space, but also, by plotting the result obtained in 10 replicates, we can test how coverage correlates with uncertainty in the placement of a particular individual. For that, we compared the replicates of individuals with different coverage rates in our dataset (Supplementary Fig. 16). Placement in the first component is stable through replicates, even for individuals with very low coverage (~3%). Coordinate values in PC2 fluctuate slightly in low-coverage genomes (~10% of the SNPs covered) and increase in individuals with lower values (~3%). However, although this phenomenon can account for some uncertainty on the placement of the individuals in PC2, it is not enough for accounting for the differentiation between the two clusters of eastern and western Canarian people.

5.2.2. Human Origins panel

The indigenous population of the Canary Islands was also compared to other available modern and ancient populations using the Human Origins panel and the lsqproject option from smartPCA[100]. To this end, we compared the Canarian natives to ancient and modern populations from North and sub-Saharan Africa, the Middle East and Eastern, Southern and Western Europe.

In this PCA, the first component separates sub-Saharan African populations from the rest, while the second component accounts for differentiation within sub-Saharan Africa (Supplementary Fig. 17). The indigenous people of the Canary Islands cluster with Eurasian populations, close to modern North Africans and Late Neolithic Moroccans. A better differentiation between Eurasian populations can be observed when PC1 and PC3 are compared (Supplementary Fig. 18). In this PCA, Late Neolithic North Africans are place between Early Neolithic Europeans and Upper Paleolithic North Africans. The Canarian natives are also placed between Prehistoric populations from Europe and North Africa, but in this case, they are

displaced towards European Middle/Late Neolithic and Bronze Age individuals. Again, some distinction can be observed between eastern and western islands, especially in PC1, creating two separate clusters with two exceptions. As we observed for the MEGA-HGDP panel, individual CAN.039 from La Angostura (Tenerife) is placed within the eastern islands cluster. This time, individual CAN.035 from Los Pasitos (La Palma) also shows the same behavior. In this case, RCD analysis indicated this individual lived around 1,157 – 1,264 cal AD, predating the contact period with Europeans.

We investigated the relationship between the indigenous people of the Canary Islands and populations from the Iron Age associated with the Roman expansion in the Mediterranean region. For that, we compared indigenous Canarians with ancient people from Egypt, Iron Age populations from the Middle East, Sardinia and Western Europe; a Punic individual from Ibiza and Punic populations from Sardinia; and Roman individuals from the Middle East, Sardinia and western and southern Europe (Supplementary Fig. 19). As shown before[88,97,103,104], from the Iron Age onwards, populations are characterized by a high mobility. In line with that observation, some Romans from western and southern Europe[97], and Punic individuals from Ibiza and Sardinia [96,103] show genetic affinities with the ancient North African cluster, including the Canary Islands. Indeed, admixture modeling analyses performed in these individuals confirm the contribution of a North African component. For example, the Punic individuals from Sardinia have relatively high levels of North African ancestry (20–35%)[103], while individuals from Rome can be modeled with up to 30–50% of a North African contribution[97]. However, although some of these individuals have a substantial contribution from this component, they do not overlap with the indigenous people of the Canary Islands, who show higher affinities with ancient North Africans (Supplementary Fig. 19).

5.3. Global ancestry

For unsupervised clustering analysis, we used the same datasets as for lsqproject PCA, but pruning for linkage disequilibrium using PLINK v1.90[105], with parameters --indep-pairwise 200 25 0.4. In addition, the global ancestry of ancient individuals was determined by unsupervised clustering using ADMIXTURE software v1.3.0[106]. The analysis was performed in 10 replicates with different random seeds, and only the highest likelihood replicate for each value of *K* was taken into consideration.

5.3.1. MEGA-HGDP panel

In Supplementary Fig. 20, we show ADMIXTURE results for the MEGA-HGDP panel with *K*=4 ancestral populations. At *K*=4, the populations are clustered in four groups roughly corresponding to the four broad geographical areas: sub-Saharan Africa (red), Europe (green), the Near East (violet) and North Africa (yellow). At *K*=4, the ancient North African people reproduce the same results observed earlier, with Upper Paleolithic and Early Neolithic individuals from Morocco composed of 100% of the North African component. However, the Late Neolithic populations show only part of their genetic composition belonging to this North African component. Regarding the indigenous population of the Canary Islands, individuals from the western islands (El Hierro, La Palma, Tenerife and La Gomera) show the same result observed for the Upper Paleolithic and Early Neolithic individuals from Morocco. Meanwhile, individuals from the eastern islands (Gran Canaria, Lanzarote and Fuerteventura) are explained as mainly corresponding to North Africa, but with a minor European contribution. This result correlates with the one observed in PCA and can explain the clustering of eastern and western

individuals of the indigenous people of the Canary Islands, with the first group having a greater European contribution and the other being more similar to ancient North African people. However, given the limited amount of modern North African and ancient Eurasian individuals, the MEGA-HGDP panel is not appropriate to resolve in fine detail the genetic composition of the indigenous people of the Canary Islands.

5.3.2. Human Origins panel

In Supplementary Fig. 21 and Supplementary Fig. 22, we show ADMIXTURE results for the modern and ancient Human Origins panel, respectively, with *K* values ranging from 2 to 8. As described before, ADMIXTURE results suggest that both Late Neolithic individuals from Morocco and the Canarian indigenous population were admixed (Supplementary Fig. 22) with an ancient Maghrebi component (yellow) and an early Neolithic European component (green)[54]. Compared with Late Neolithic Moroccans, the Canary Islands' indigenous people have a greater contribution from the ancient Maghrebi component. Also, the Canarian natives present a component that maximizes in European hunter-gatherer and Steppe populations (blue), but is absent in Late Neolithic Moroccans, which could explain the behavior of the individuals in the PCA. As the human colonization of the Canary Islands archipelago has been estimated to have happened between the first-century BC and the second-century AD[5,107], additional migration waves are expected to have reached North Africa by that time. Given the presence of Bell-Beaker pottery in the North African archaeological record[79], this steppe component could be related to the expansion of European Bronze Age populations in North Africa[54,102]. Also, some individuals have a component associated with Neolithic Iranians (grey) that is present in ancient and modern populations from the Near East but also in Bronze Age people from the steppe. Although from a mitochondrial DNA perspective, a sub-Saharan African input in the indigenous people of the Canary Islands is inferred from the presence of L haplogroups, the unsupervised clustering analysis only shows a small sub-Saharan contribution (red) in a few individuals, and in all cases, the observed values are below 5%.

Interestingly, some differences are observed between western and eastern populations. At *K*=8, individuals from the western islands show a higher proportion of the autochthonous Maghrebi component and a lower contribution associated with steppe populations when compared with eastern islands (Fig. 2b). This differentiation can be observed both comparing the different islands using a ternary plot depicting the main components present on the indigenous population (Supplementary Fig. 23) and a boxplot showing the contribution mean of each component per island (Supplementary Fig. 24). On the other hand, the contribution of the European Neolithic and the Near Eastern components are similar in both regions (Supplementary Fig. 24).

When compared to the current population of the Canary Islands, an increment is observed in the component likely associated with the Steppe contribution and the Near Eastern component, and a lower autochthonous North African contribution (Fig. 2b). This result is expected giving the fact that the modern population of the Canary Islands is admixed with the main parental populations being the indigenous people and European colonizers.

Giving the time of the human colonization of the Canary Islands, it is also interesting to compare them to modern North Africans, to get an insight of the impact that later migrations had on the region (Fig. 2b). Modern North African people exhibit a lower proportion of the ancient Maghrebi component when compared to the Canarian natives. As this component is higher in western North Africa [101], the greatest differences are observed for Egypt and Libya. We also observe a greater contribution from the Near Eastern and Sub-Saharan African components in

modern North Africans. These differences correlate to additional migration reaching this area in the last two millennia, including the Arab expansion in the seventh century and the trans-Saharan migrations.

The high proportion of the Maghrebi component observed in the Canary Islands indigenous people is indicative of their origin being related to Berber populations in North Africa, a hypothesis that has been supported by extensive archaeological, linguistic, and genetic analyses[66,73,107–122]. The inclusion of other historical individuals in the PCA allows us to test some controverted hypotheses that have been proposed for the origin of the indigenous people (Supplementary Fig. 19; Supplementary Fig. 23). Based on radiocarbon data obtained from bulk sediment samples in the archaeological site of Buenavista in Lanzarote[112], some archaeologists have proposed chronologies that go as far as the 5th century BCE[112–114,121], leading them to propose a Phoenician origin for the first settlers of the Canary Island[112–114,123]. However, a recent review of this data and the application of radiocarbon hygiene criteria have shown that these older dates for the colonization of the Archipelago are not reliable[109]. Furthermore, the archaeological record show no clear cultural adscription with Phoenicians. Specifically, archaeological evidence is composed of small pieces of pottery from non-diagnostic regions and excavated in a site with a questioned stratigraphy that cannot discard the intrusion of materials from other periods. For those reasons, scholars have contested the affiliation to this site to the Phoenician culture[115]. Regarding an Egyptian origin for the indigenous people of the Canary Islands, although some scholar traditions up to the XIX century proposed a link between Ancient Egypt and the islands based on the practice of mummification in both regions, archaeologists promptly disregarded it. However, this association between Egypt and the Canary Islands has remained in the collective imaginary[116]. As for the hypothesis on the Roman colonization of the islands, a few aspects should be taken into consideration. The purple dye workshop in the islet of Lobos is the only site with a clear Roman adscription. However, its archaeological record shows no interaction between this population and the Canarian indigenous people[110,111]. In this sense, absolute radiocarbon dates from this site point to the 1st century BC, previous to the proposed date of the colonization of the islands (between the 2nd and 5th centuries AD). Also, the materials found in this site, especially Roman amphoras, provide a relative chronology that situates this settlement in a relatively short period (between 50 and 100 years), showing its limited impact on the population of the Canarian Archipelago as a whole[33,109].

Based on the ADMIXTURE results observed for the indigenous people and the historical individuals included (ancient Egyptians, Phoenician and Etruscan), the natives of the Canary Islands have a higher ancient North African component than Egyptians and the Phoenician individual from Ibiza (Supplementary Figures 23 and 24). Also, their percentage of the autochthonous Maghrebi component is higher than the Etruscan individual with North African admixture published by[97]. Egyptian individuals are characterized by having a higher Iran Neolithic contribution, while the Phoenician and Etruscan individuals have a higher steppe component than most of the individuals from the Canarian archipelago. Based on those results, it is unlikely that the origin of the natives could be solely related to a single migration event from these populations.

Supplementary Note 6: *f*-statistics analyses

We performed the analysis of outgroup *f3*-statistic using qp3Pop program from ADMIXTOOLS (https://github.com/DReichLab/AdmixTools), to determine the amount of shared drift between two ancient populations PopA and PopB. For that, we chose an outgroup population that has not experience any post-divergence gene flow with either PopA or PopB as the target population (e.g. Jo'hoan North), and calculated the *f3*-statistic in the form *f3(PopA, PopB; Outgroup)*. In this way, the result of the *f3*-statistic will be a positive value proportional to the length of the shared drift path of populations PopA and PopB with respect to the outgroup.

Considering the populations contained in the Human Origins dataset, the indigenous people of the Canary Islands share more ancestry with Iberian, Aegean and other European Early Neolithic populations (Supplementary Fig. 25), similarly to the results obtained for Late Neolithic Morocco[54].

PCA and unsupervised clustering results indicate that the eastern islands have a greater contribution from a component related to the Bronze Age expansion in Europe, while western islands show a higher ancient North African contribution. To test if differences are observed between islands, we repeated the outgroup *f3*-statistic calculations at insular level and compared the amount of shared ancestry with populations of interest. Differences were observed between the amount of shared ancestry between islands populations with Neolithic and Bronze Age populations from Europe, with the eastern islands producing higher values. Figure S26 shows the results obtained for Middle Neolithic to Bronze Age populations from Western Europe and Late Neolithic to Bronze Age populations from the steppe. When compared with Early and Late Neolithic individuals from North Africa, slightly differences between eastern and western islands are observed for the Late Neolithic population, but not for the Early Neolithic population. However, outgroup *f3*-statistics are difficult to compared and do not provide information about the contribution of different populations, and a more detailed analysis using admixture modeling is needed.

Supplementary Note 7: qpAdm modeling

Admixture modeling was performed using qpAdm from ADMIXTOOLS, following[124] and using the Allen Ancient DNA Resource curated by the Reich Lab at Harvard (https://reich.hms.harvard.edu/allen-ancient-dna-resource-aadr-downloadable-genotypes-present-day-and-ancient-dna-data, version 42.4). First, we defined the target as the whole population of the Canarian archipelago. For the right populations, we chose the same outgroups as in[87]: Mota, Ust-Ishim, Kostenki14, GoyetQ116-1, Vestonice16, Malta1, AfontovaGora3, ElMiron, Villabruna, WHG, EHG, CHG, Iran_N, Natufian, Levant_N and Anatolia_N. For the left populations, we started with the simpler model (one stream of migration) and increased complexity until reaching statistical significance. With the aim of exploring different admixture scenarios, we implemented a "rotating" scheme, in which populations are systematically moved from the set of reference populations to the set of sources.

For the one-stream model, no statistical significance was reached for any of the tested target with any of the possible contributing populations (Supplementary Data 3). However, the one-stream model with the p-value closer to significance was the one involving one stream of migration from Morocco_LN. The same result was obtained for the two-stream and three-stream models, with no populations' combinations reaching significance. Again, the two models closer to significance involved the contribution from Morocco_LN (2-stream model: Morocco_LN + Mota; 3-stream model: Morocco_LN + Mota + Russia_EBA Yamnaya). Considering the whole indigenous population, the simpler admixture models reaching significance involved the contribution of Morocco_LN, Mota, a source of autochthonous North African ancestry (either Morocco_IB or Morocco_EN) and a source of steppe ancestry (either Germany_BellBeaker, Russia_EBA_Yamnaya or Russia_HG_Karelia).

The best-fitting model is Morocco_EN (6.9% ± 1.0%), Morocco_LN (73.3% ± 2.2%), Germany_BellBeaker (13.4% ± 1.8%) and Mota (6.4% ± 1.3%) (Fig. 2c; Supplementary Data 3). This result indicates that most of the Canarian indigenous people's ancestry can be linked to Late Neolithic populations from Morocco. However, a single pulse from this population is not enough to account for their genetic composition. First of all, ancient Canarians need additional input from the North African Maghrebi component. As proposed earlier[54], this result can be explained considering that the indigenous people originated in a different area of the Maghreb where most probably the European Neolithic impact was lower. As observed for the mtDNA, additional pulses of migration from a sub-Saharan African and Eurasian Bronze Age sources are needed to model the indigenous people of the Canary Islands. Given that the colonization of the Canary Islands occurred long after the Late Neolithic period (~3,000 BCE), most probably around the first centuries CE, it is expected that additional waves of migration had already reached North Africa. Regarding the existence of trans-Saharan migrations, genome-wide data from modern North Africans[101] provided evidence of the contribution of sub-Saharan Africans into Morocco around 1,200 years ago. This date coincided with the rise of the Ghana Empire, which was involved in the trans-Saharan slave trading. However, our results indicate that sub-Saharan African migrations already reached North Africa by the time of the colonization of the islands (approx. 2000 BP). When testing modern populations instead of Mota for admixture modeling, Datog people from Kenia (8.9% ± 1%) and Somali from Tanzania (10.6% ± 1.2%) showed the highest correlation values as contributors to the indigenous population of the Canary Islands. However, as considering modern and ancient individuals as left populations may introduce bias in the results[124], we consider Mota as the best source population for the sub-Saharan contribution. In the same vein, the existence of a European Bronze Age component can be

explained by the presence of Bell-Beaker pottery in the North African archaeological record[66,102]. It is worth mentioning that models with Iberia_BellBeaker do not reach significance.

Then, we applied the best four 4-stream models to determine differences between a) the two geographical regions (eastern and western islands), b) each island population (El Hierro, La Palma, La Gomera, Tenerife, Gran Canaria, Fuerteventura and Lanzarote) and c) each individual. When comparing the eastern and western regions (Supplementary Data 5), we observe that western islands have a higher contribution of Morocco_EN (8.3% ± 1.1%) than eastern islands (4.9% ± 1.1%). Also, the Germany_BB contribution is slightly higher in the eastern islands (16.0% ± 2.0%) than the western islands (11.4% ± 1.9%), while the rest of the components remain relatively stable. If we look at each island by itself, we observe that La Palma has the highest Morocco_EN contribution (10.1% ± 1.7%), while Lanzarote has the highest Germany_BB contribution (17.9% ± 3.5%). At an individual level, individuals from the sites of Guayadeque (CAN.018; 20.2% ± 4.4%) and El Hormiguero (CAN.012; 19.0% ± 3.4%) in Gran Canaria show the highest Germany_BB proportion (Supplementary Data 6). The lowest values are observed in Antoncojo in La Gomera (CAN.027; 8.10% ± 3.60%) and Punta Azul in El Hierro (CAN.003; 9.20% ± 3.10%). The opposite occurs for the Morocco_EN contribution: individuals from the sites of Cendro (CAN.050; 3.4% ± 2.5%) and Guayadeque (CAN.014; 4.0% ± 2.3%) in Gran Canaria show the lowest Morocco_EN proportion (Supplementary Data 6), while the highest values are found in Salto Casimiro en La Palma (CAN.036; 10.4% ± 1.8%) and a decontextualized individual from Tenerife (gun012; 10.2% ± 2.0%). Interestingly, when analyzed in combination with radiocarbon data, we observe that the slight differences between eastern and western regions are observed for the entire period of indigenous occupation (Supplementary Fig. 27). This result is more clearly seen for the two islands with a larger sample size: Tenerife and Gran Canaria. When the Morocco_EN contribution is compared with radiocarbon data obtained from the individuals, we observed that values are relatively lower in Gran Canaria for the entire indigenous occupation period (Supplementary Fig. 28).

Although the presence of a steppe component in the indigenous people of the Canary Islands can be explained based on a Bronze Age migration to North Africa, it can also be the result of the impact of Punic or Roman populations in North Africa before the settlement of the archipelago. To test this hypothesis, we repeated the four-stream qpAdm modeling but replacing German Bell Beakers by available Roman and Punic populations (Supplementary Data 4). The only working models involved Roman and Punic populations with either a North African or Middle Eastern admixture. These populations include Roman individuals from England[125], a Punic individual from Ibiza[96], and Punic individuals from the Villamar site in Sardinia[103]. The best-fitting model involves the admixture of Morocco_EN (8.9% ± 1.4%), Morocco_LN (41.3% ± 7.9%), Mota (4.7% ± 1.6%) and Ibiza Punic (45.1% ± 7.3%) (Supplementary Data 4). However, this result should be taken with caution as data from Punic individual from Ibiza comes from a low coverage genome (0.47X). Apart from that one, the following best-fitting model involves the admixture of Morocco_EN (10.7% ± 2.2%), Morocco_LN (20.9% ± 16.6%), Mota (11.2% ± 1.6%) and Sardinia Punic (57.2% ± 13.8%) (Supplementary Data 6). These results indicate that another explanation for the presence of steppe ancestry in North Africa could be the admixture of local Berber populations with incoming Punics or Romans. It is worth mentioning that 5-stream model including both a Bell-Beaker and a Roman/Punic contribution did not reach significance.

One limitation of our study is that sample sizes are low for some islands, for example, Lanzarote and Fuerteventura with only two individuals. To account for sample size differences, we compared the qpAdm admixture values obtained at individual level by island (Supplementary

Fig. 29). Mean values for the contribution of Early Neolithic Moroccans to western islands (8.60% ± 0.57% for El Hierro, 7.67% ± 1.91% for La Gomera and 8.82% ± 1.09% for Tenerife) was higher than that of those of the eastern island of Gran Canaria (5.54% ± 1.20%). The only overlapping value between the western and eastern regions is the one calculated from the individual CAN.027 from La Gomera, but never reaching values as low those observed for the islands of Lanzarote and Fuerteventura. Hence, although some variation is observed within individuals from the same island, a regional differentiation between eastern and western islands is confirmed.

<u>Supplementary Note 8</u>: Heterozygosity, inbreeding and family relationships

8.1. Family relationship estimations

Family relationships between individuals were estimated using READ software[126] based on the MEGA-HGDP genome-wide data. This method allows us to infer up to second-degree relationships, including nephew/niece-uncle/aunt, grandparent–grandchild or half-siblings. This approach is especially appropriate for kinship inferences on ancient DNA data, as it can produce reliable results for individuals with coverage greater than 0.1X. To avoid lack of coverage to confound the estimations, we excluded CAN.043 from Tenerife and CAN.046 and CAN.039 from Gran Canaria (<0.2X covering the MEGA-HGDP dataset). We only detected two individuals from Tenerife (CAN.045 and CAN.046) with an average pairwise P0 coinciding with a second-degree relationship (Supplementary Fig. 30). The rest of the individuals from the islands showed no apparent kinship between them.

8.2. Heterozygosity estimations

Heterozygosity estimates were obtained for each island population and for each archaeological site using popstats[127]. For this analysis, we used the MEGA-HGDP to increase the individuals' coverage and retained only transversions to avoid damage to interfere with results. Only those archaeological sites with at least two individuals were selected: Punta Azul from El Hierro; Barranco Majona from La Gomera; El Portillo from Tenerife; El Hormiguero, Guayadeque, Lomo Galeón, Puente de La Calzada and Cendro from Gran Canaria; and Montaña Mina from Lanzarote. Heterozygosity values were calculated for the Early and Late Neolithic people from Morocco using the same procedure for comparison, considering that the first one is population showing signs of isolation and the other one was the result of admixture.

Gran Canaria, Tenerife, and La Palma showed the highest heterozygosity estimations (0.219 ± 0.001, 0.218 ± 0.001 and 0.215 ± 0.001, respectively) close to Late Neolithic populations from Morocco (0.213 ± 0.001) (Supplementary Fig. 31). On the other hand, Fuerteventura showed the lowest heterozygosity estimates (0.184 ± 0.003) followed by Lanzarote and El Hierro (0.193 ± 0.002 and 0.195 ± 0.001, respectively). La Gomera shows intermediate values (0.198 ± 0.001). However, all of them outgrow values seen for Early Neolithic populations from Morocco (0.157 ± 0.001). When sites are compared (Supplementary Fig. 32), Punta Azul from El Hierro has the lowest heterozygosity value (0.196 ± 0.001) as inferred using mtDNA [36,66], while Puente de La Calzada in Gran Canaria showed the highest (0.260 ± 0.016). Except for Punta Azul, the rest of archaeological sites are above the *pi* estimations obtained for Late Neolithic populations from Morocco (Supplementary Fig. 32). These results confirm previous diversity estimations based on uniparental markers[66] in which islands with more exploitable natural resources (Tenerife, Gran Canaria and La Palma) had more diversity than smaller islands or islands with more limited resources.

To account for sampling bias, heterozygosity values were estimated for all possible combinations of two individuals within each island (the lowest sample size, from Fuerteventura and Lanzarote) and the average value was calculated. To mirror the individuals of Lanzarote and Fuerteventura, we selected for heterozygosity estimations one individual with a low-coverage genome available and one captured individual. Again, heterozygosity estimations are variable depending on the individuals involved in the calculation, but mean and median values for the

islands of Tenerife and Gran Canaria are higher than those observed for El Hierro and La Gomera (Fig. 3a). In order to explore the variation on the heterozygosity estimations, we focused on the better characterized islands of Tenerife and Gran Canaria (Supplementary Fig. 33). Variation on the heterozygosity values estimated by pairs of individuals can be explained by low overlap within the two compared individuals, with lower coverages tending to produce lower heterozygosity estimations, with a moderate correlation between the *pi* value and coverage (Tenerife: r=0.449 and p=0.0020; Gran Canaria: r=0.510 and p=0.0078). However, no combination of two individuals produced values as low as those observed for Lanzarote (*pi* = 0.193) and Fuerveventura (*pi* = 0.184). Even though these values could be an underestimation of the real values for Lanzarote and Fuerteventura, the results obtained point to a lower heterozygosity value for these islands, as observed for El Hierro and La Gomera.

8.2. Inbreeding estimations

The presence of runs of homozygosity (ROHs) was detected using hapROH[128] for both the Human Origins and the MEGA-HGDP datasets. In this method, haplotype information from a modern DNA panel is used to detect ROHs longer than 4 cM in aDNA genomes with coverage as low as 0.3X. We screened for the total sum of ROH segments in four length ranges (4 – 8, 8-12, 12-20 and >20 cM) in individuals with genome coverage higher than 0.3X.

First, we focused on short ROH segments of 4 – 8 cM ($ROH_{[4,8]}$) that accumulate from parents on 1 – 30 generations ago and are indicative of the population size of the ancestral mating pool (background relatedness) over approximate the previous half millennium[128]. $ROH_{[4,8]}$ is high among the most ancient individuals and then decreases forward in time due to population expansions (e. g., the Neolithic transition). Most of the Canary Islands indigenous individuals have at least 20 – 60 cM of their genome composed of $ROH_{[4,8]}$ (Fig. 3b; Supplementary Data 8), which is indicative of relatively small effective population in the past, probably on the earliest stages of the human settlement.

Second, we tested our individuals having >20 cM ROH ($sROH_{>20cM}$) segments greater than 50 cM, which is indicative of small and isolated populations[128]. We identified five ancient individuals exceeding this long ROH threshold: CAN.004 from El Hierro, CAN.005 from Fuerteventura, CAN.030 from La Gomera, CAN.038 from Lanzarote and CAN.038 from Tenerife. Interestingly, the four of them with the greatest $sROH_{>20cM}$ and total ROH exceeding 300 cM are from either islands with the smallest areas and/or with poorer resources. This amount of ROH can be due to sporadic close-kin matings due to small population sizes. Also, all individuals are from the latest stages of the indigenous period of the Canary Islands (from 12th century onwards) which can be indicative of the effect of isolation in the archipelago population.

Supplementary Note 9: Effective population size and founder effects

9.1. Effective population size

Estimates of effective population size (N_e) for insular populations were obtained using hapROH[128] for HGDP-MEGA dataset as it is the one with a better coverage (Fig. 4a). Considering the whole population of the archipelago, we observe that the N_e was maintained in 412 individuals (95% CI: [371 – 460]), slightly lower than previously reported considering only ancient genomes from Gran Canaria and Tenerife[129]. Also, when considering how N_e evolved around the 1,200 year-transect we have sampled, we observe a decreasing N_e pattern over time when individuals from all the archipelago are included. When the indigenous population of each island is analyzed separately, we observe that Mahos from Fuerteventura (mean: 75; 95% CI: [56 – 101]) and Lanzarote (mean: 151; 95% CI: [106 – 221]) exhibit the smallest N_e, followed by Bimbapes from El Hierro (mean: 204; 95% CI: [163 – 263]). People from Gran Canaria had the highest N_e estimations (mean: 460; 95% CI: [366 – 594]), followed by and Beneahoritas from La Palma (mean: 395; 95% CI: [272 – 599]) and Guanches from Tenerife (mean: 285; 95% CI: [227 – 366]), coinciding with previous heterozygosity estimations. Surprisingly, La Gomera has the second-largest N_e estimations of all the islands (mean: 429; 95% CI: [292 – 664]). However, we have to consider that our individuals from La Gomera consist of one individual from an earlier occupation phase of the archipelago (CAN.027; 9th century) and the other ones from a later period (12th – 14th centuries), while individuals from Fuerteventura, Lanzarote and El Hierro are all from the late occupation phase (12th century onwards). If inbreeding increased over time, this difference may affect N_e estimations. When we removed CAN.027 from the analysis, we obtained a more reduced N_e for indigenous Gomerans (mean: 281; 95% CI: [180 – 473]).

As N_e estimations for the islands of Lanzarote and Fuerteventura are calculated from only one individual, it is possible that the actual N_e values were higher. We have to consider that the two individuals from Lanzarote and Fuerteventura presented values of $sROH_{>20cM}$ higher than 50, and therefore they should have been excluded from N_e estimations. For the island of El Hierro, we calculated N_e including individuals with a substantial portion of their genomes in ROHs and as expected, the N_e value was lower than the one calculated before (mean: 147; 95% CI: [123 – 177]). A similar result is observed when individuals with ROHs are included for La Gomera and Tenerife (Supplementary Data 9). In order to further explore the effects of the sample size limitation in Lanzarote and Fuerteventura, we also estimated N_e considering only one individual as representative of each island population. For that, we selected individuals with similar a genome-wide coverage as the ones from Lanzarote and Fuerteventura (Supplementary Data 9). We observed that for all the islands, the N_e confidence intervals estimates are considerably wider than when using the original sample size. As expected, we also observed that for different individuals of the same island, N_e estimates based on one individual vary depending on the proportion of his/her genome on ROHs. For all the above, although the inbreeding level observed in the only two available individuals from Lanzarote and Fuerteventura are a testament of the small N_e of these island populations, estimates for these islands should be reevaluated when additional paleogenomic data is available.

9.2. Founder effects

To evaluate the history of reductions in population size in the indigenous populations of the Islands, we used ASCEND[130]. ASCEND relies on the decay of the allele sharing correlation

across individuals in a population to infer the time and intensity of a founder/bottleneck event to low-coverage genomes. We followed the recommendations from the authors and ran the analysis for four different datasets from the MEGA-HGDP panel: (1) all indigenous individuals, (2) individuals divided by their geographical location (eastern –Lanzarote, Fuerteventura and Gran Canaria– and western –Tenerife, La Gomera, El Hierro, La Palma–); and (3) individuals from each island with at least N≥5 (Gran Canaria and Tenerife). To avoid confounding bias, we excluded CAN.039 from the analyses due to possible post-conquest admixture, and filtered out CAN.046 because of the 2nd-degree relatedness with another individual from Tenerife. In order to inspect if our results were determined by individuals's coverage, we filtered out genomes with <0.1X and <0.3X of coverage from each dataset. To obtain the time of the bottleneck or founder events, we considered the average radiocarbon date of each dataset and 28 years per generation. For all the analyses, the NRMSD estimator was below the recommended cut-off of 0.29 (median = 0.04).

According to the results, a shared founder effect for all the indigenous people occurred around 720 BCE (95% CI: 944 – 440 BCE) coinciding with the 5th to the 10th centuries BCE (Supplementary Data 10; Supplementary Fig. 34). This reduction in population occurred with a low-moderate intensity (95% CI: 1.7 – 2.0) (Supplementary Fig. 35) more similar to founder events seen in continental populations than to island ones[130]. Among islanders, only Maltese or Orcadians have shown similar intensities. Moreover, island populations tend to experience more drastic founder events due to lower founder population sizes and/or maintained low population sizes within the island due to resource limitation. Considering that different islands most probably experienced bottlenecks of different intensities, this result can be either an artifact or reflect a common bottleneck that affected the ancestors of the Canarian indigenous people in the continent, previous to the colonization time.

Considering the ancestry differences between eastern and western individuals commented before, we run the analysis for each geographic location. Western islands would share a bottleneck event that occurred around the Era change (95% CI: 184 BCE – 208 CE), while eastern individuals would share an earlier event between the 8th and 2nd centuries BCE (95% CI: 793 BCE – 121 BCE). The intensity of the bottleneck event is similar in both the eastern and western regions (on average 3.1% and 2.7%, respectively) sharing the continental-like characteristic with the Archipelago's. However, this analysis can also produce artifactual results as different islands could have experienced founder effects of different intensities.

If we consider Gran Canaria alone, a possible bottleneck event is estimated between the 4th century BCE and the 2nd century CE, similar to the eastern's potential bottleneck in intensity and time range (95% CI: 2.3% – 2.9%). This result is expected because most of the eastern people are from Gran Canaria. Alternatively, individuals from Tenerife could have experienced a more drastic event (95% CI: 3.7% – 4.7%) than the western people but in the same period (95% CI: 242 CE – 66 BCE), similar to those seen in present-day islanders[130].

When we filtered individuals based on their SNP coverage, we do not see significant differences in the bottleneck events' dating, except for Gran Canaria and the eastern dataset (Supplementary Fig. 34). As Gran Canaria is the island from which we have sampled a more ample territory (all of the individuals from Tenerife are from a very restricted area of the island) this result may not be due to coverage itself but to sample diversity.

Even though the minimum recommended sample size N≥5, as we see that coverage and sample size does not seem to affect results in islands such as Tenerife, we ran the analysis with the El Hierro individuals. In fact, although there are four individuals, only one of the genomes is below 1X of coverage. Using that dataset, we detected that El Hierro individuals shared a bottleneck event occurring between the 8th and the 12th centuries CE, few generations before the

individuals lived. The intensity of the event is ~12%, considerably greater than the rest of the events studied before. However, the fact that all of them are from the same archeological site and close in time could be affecting the results. More genome-wide data from El Hierro will be needed to determine if this event affected all the island population.

Supplementary Note 10: Phenotype analyses

The Canarian indigenous genomes were assessed for phenotypical traits by analyzing the alleles present at a population level in several loci of interest (Supplementary Table 1). For that, we performed SNP calling using samtools mpileup, filtering out low-quality bases (BASEQ < 30) and low-quality mapped reads (MAPQ < 25). Due to the low coverage of the genomes, we only considered one random allele for each position for all the variants (pseudo-haploid calling) and studied the variants present in the indigenous pool with depth > 1.

First, we assessed the five known *MCM6* SNPs associated with lactose tolerance (rs4988235G>T, rs41380347A>C, rs41525747G>C, rs145946881C>G, rs869051967A>C) [131]. All indigenous individuals carried the ancestral alleles for all the variants, except for one individual with two alleles for the rs4988235A variant (Supplementary Table 1). Probably the most common phenotype in the Canary Islands indigenous people was lactose intolerant as previously seen[68].

We also reviewed the bibliography and considered several medical conditions that are common in present-day Canarians when compared to the Iberian Peninsula, in order to prove if their associated variants were already presented in the indigenous gene pool (Supplementary Table 1). The *PLCG2* gene has been associated with autoimmune conditions such as asthma in present-day Canarians, at high frequency when compared with the Iberian founder population, and has been linked to their North African ancestry[132]. We found that the derived variant associated with asthma in this gene (rs3852738T) is found in a frequency of 79.2%, so the indigenous origin proposed by the authors is plausible.

Supplementary Table 1. Phenotypically informative SNPs.

Gene	SNP ID	Effect Allele	Association	Reference	allele				N	Effect allele frequency
					A	C	G	T		
**Carbohydrate metabolism**										
MCM6	rs4988235	A	Lactase persistence	Ref. [131]	2	0	20	0	11	0.09
MCM6	rs41380347	C	Lactase persistence	Ref. [131]	18	0	0	0	9	0.00
MCM6	rs41525747	C	Lactase persistence	Ref. [131]	0	0	22	0	11	0.00
MCM6	rs145946881	G	Lactase persistence	Ref. [131]	0	26	0	0	13	0.00
MCM6	rs869051967	C	Lactase persistence	Ref. [131]	26	0	0	0	13	0.00
**Medical conditions in Canary Islanders**										
PLCG2	rs3852738	T	Asthma Canary Islanders	Ref. [132]	5	0	0	19	12	0.792

Supplementary Note 11: Admixture proportions for the modern Canarian population

Admixture analysis using mtDNA determined that the contribution of the indigenous people to the modern Canarian population was 55.9% for the entire archipelago[66]. When considering the islands independently and using the total indigenous population for calculations, the native contribution ranged from 30.7% in Gran Canaria to 71.4% in La Gomera. When using the indigenous people of each island as parental populations, values ranged from 0% in El Hierro to 55.5% in La Gomera[66]. The result obtained for El Hierro was interesting, because when the whole indigenous population was used, the native contribution was 36.2%. This result was explained considering that the island of El Hierro was almost depopulated at the time of the European conquest and was later repopulated with indigenous populations from other islands and European colonizers[66]. This result was important because it highlighted the necessity of considering each island independently.

Regarding the Y-chromosome, previous admixture analysis estimated a contribution of indigenous males to the modern Canarian population of 5.9 ± 2.9%, using North Africans as a proxy for the indigenous population[133]. This result demonstrated that the modern population of the Canary Islands exhibit a marked sexual asymmetry on the survival of indigenous lineages: while mtDNA indicates a relatively high indigenous contribution (>50%), Y-chromosomes in the current population are mostly of European origin (>90%). Direct ancient DNA analysis of the Y-chromosome of the indigenous population produced an estimate of 16.1 ± 4.6% contribution of native males to the current population[73]. The presence of indigenous Y-chromosomes can explain the higher frequency of E-M183 in current and historical Canarian populations (8.3%, 11.9%)[73,134] when compared to the Iberian Peninsula (5.2 %)[135,136]. Using our new dataset, we repeated the Y-chromosome admixture analysis using the mL estimator as in[73], considering three main parental populations: the Canarian indigenous population, and current Iberian and Sub-Saharan populations. Admixture estimator mL was calculated based on Y-chromosome haplogroup frequencies using the WLSAdmix program[137]. For these admixture estimations, we only included ancient NGS individuals, with the aim of avoiding putative problems derived from analyzing nuclear DNA with PCR and from sample bias due to most of the individuals coming from the sites of Guayadeque and Punta Azul (see section 4.2). From a Y-chromosome perspective, most of the lineages present in the current Canary Islands population are of European origin (95.3%), while the indigenous and sub-Saharan African components accounting for 4.0% and 0.7%, respectively. Values within islands vary, with no indigenous components being observed in La Gomera and El Hierro, and with the highest values present in Fuerteventura (12.5%) and Gran Canaria (9.1%). Sub-Saharan African contribution is absent in Fuerteventura, La Gomera, El Hierro and Tenerife, and low values are observed in the remaining islands. Because our sample size is still too low at an insular level, the comparison between calculations performed using the total indigenous people and each insular population could not be carried out.

We also performed admixture estimations based on genome-wide data using qpAdm from ADMIXTOOLS as described for the ancient individuals, and considering the indigenous, Spanish and Yoruban populations as contributing sources. For that we merged the array data from the Canary Islands obtained by[132] with the Human Origins data, obtaining a total of 55,516 SNPs. For the total modern Canarian population, the admixture model would imply 17.8% ± 1.3% of indigenous people, 79.7% ± 1.0% of Spanish and 2.6% ± 0.5% of Yoruban contributions. To calculate the contribution of each parental population at an individual level, we used both the global indigenous population and each respective insular indigenous population.

When the global indigenous people are considered, the islands with the highest indigenous contributions are observed for El Hierro (25.2% ± 1.8%) and Fuerteventura (23.2% ± 1.4%), while the lowest are observed for La Palma (12.9% ± 1.4%), Gran Canaria (13.4% ± 0.9%) and Tenerife (14.2% ± 1.1%) (Supplementary Fig. 36). The sub-Saharan contribution is low in all the islands, with the highest value observed in La Gomera (4.0% ± 0.5%) and the lowest in La Palma (1.1% ± 0.5%) (Supplementary Fig. 36). When insular indigenous populations are considered for calculations, the contribution of the native people is higher in La Gomera, Lanzarote and Fuerteventura, although we cannot rule out the possibility that this result could be due to low sample sizes, as the two islands with the larger numbers of individuals (Tenerife and Gran Canaria) produce similar results in both analyses.

Regarding of the method used for obtaining the admixture proportions for the modern Canarian population, it is clear the impact that the European colonization had in the indigenous people. Although the contribution from the mtDNA is more than 50%, this value is lower than 5% when the Y-chromosome is considered. Genome-wide data point to an overall contribution around 17.8% ± 0.8%, similar to the value obtained from[68] (16.7% ± 5.0%). As was previously observed for the mtDNA, the contribution of the indigenous people to current Canarians is variable between islands. From the Y-chromosome, we observe than the contribution is almost zero for the smallest islands of El Hierro, La Palma and La Gomera. Higher values are observed for the larger islands of Gran Canaria (9.1%) and Tenerife (8.2%). It is interesting that the island of Fuerteventura has a contribution of 12% while Lanzarote, with a similar conquest and colonization history, has a value of 1.2%. As the Y-chromosome composition was most probably variable within the archipelago as observed for the mtDNA, all these analyses would need to be repeated when enough Y-chromosome data is available to perform calculations at an insular level. Regarding genome-wide analyses, it is expected from historical data that the islands that have received more migration since colonial to modern times, are the ones with the lowest indigenous and the highest European contributions. This includes the two islands with the capital cities of the provinces of Las Palmas de Gran Canaria and Santa Cruz de Tenerife, but also the island of La Palma. The island of La Palma and Tenerife had an important arrival of European populations, mainly Portuguese and Castillians since the beginning of the Modern period. Although the sub-Saharan African contribution is low, this result is also interesting as it demonstrates the impact of the slave trade in the Canary Islands and the incorporation of freed enslaved people into the Canarian society as demonstrated by[138].

Supplementary Note 12: IBD analyses

We imputed the genomes using GLIMPSE v1.0.1[139] with the default parameters as recommended by[140]. Given that 0.1X coverage is enough to get 80% of imputation accuracy at common variants (minor allele frequency >= 10%), only >0.1X individuals (N=22) were selected for the imputation step (Supplementary Data 1). We first computed genotype likelihoods for each genome using the candidate variant sites contained in the 1000 Genomes phase 3, filtering out for non-biallelic and singleton variants. We split the chromosomes into 2 Mb chunks using 200 Kb of buffer size with GLIMPSE_chunk. Each chunk was then imputed and phased using GLIMPSE_phase and ligated into the same chromosome using GLIMPSE_ligate. The most likely haplotypes (--solve parameter) were then sampled from the ligated VCFs. We filtered out the individuals with an average genotype probability (GP) lower than 0.95 and removed variants with an INFO score <0.5.

To analyze the population structure among ancient Canarians and between them and other ancient European and African populations in finer detail, we identified shared genomic segments that are identical by descent (IBD). IBD segments are identical haplotypes that two individuals share when inherited without recombination[141]. To obtain IBD segments we built a database using already published genomes from Neolithic to Iron Age Europe, sub-Saharan Africa, the Near East and Neolithic to Late Neoltithic North Africa[54,85,87,88,90,94,95,97,103,142–146]. We selected the individuals with an average coverage >0.1X and genotype probability (GP) >0.95, imputed them and merged with the imputed ones from the Canary Islands. We then recalculated their INFO score and filtered out variants with an INFO score <0.5. From the resulting VCFs, we called the IBD segments using IBDseq[141] with the default parameters. We converted the IBD segments from base pair to centimorgan (cM) using the HapMap II haplotype panel as reference. Finally, we carried out genetic clustering of the ancient individuals using hierarchical community detection on a network of pairwise IBD-sharing similarities, as in[140]. Briefly, we used igraph[147] to build a weighted network of the individuals using the fraction of the genome shared IBD between pairs as weights, and used this network to perform community detection using the Leiden algorithm[148] implemented in the *leidenAlg* R package (https://github.com/kharchenkolab/leidenAlg).

We observe that a single genetic cluster for all the African individuals was created separating them from European people (Supplementary Fig. 37). It is worth mentioning that this cluster does not contain the Late Neolithic people from Morocco, that are placed with Chalcolithic and Early Bronze Age individuals from Europe. The African cluster identifies five communities: three for the Canary Islands people and two for the rest of the ancient North African and sub-Saharan African individuals. Focusing on the Canarian ones (N=22), we observe that this analysis separates all individuals from the eastern islands from those of the western ones, except for El Hierro individuals who are placed in a specific cluster (Fig. 4b), as expected for individuals who share a high fraction of their genome in IBD and consistent with consanguinity due to isolation. When focusing on the western cluster, we observe that, as expected in isolated insular environments, individuals from the same island share a higher fraction of their genome in IBD, clustering them together. One of the cluster groups La Gomera and La Palma individuals separated from the Tenerife individuals, and then each island in their own cluster. In the case of the eastern islands, the individuals seem to be more difficult to group together than the western ones, as the fraction of the genome shared IBD within pairs is lower. Although it is possible to group the Fuerteventura and Lanzarote individuals separated from the ancient Gran Canarians, the difference is not as significant as in the western people. We can

observe that those individuals are more related to CAN.008 (Agujero) and CAN.017 (Guayadeque) than any other individual from Gran Canaria. The other two Gran Canaria clusters are formed by individuals from potentially the same archaeological site. Individuals CAN.049 to CAN.051 belong to the Cendro site, while gun005 and gun008 belong to the University of Edinburgh Museum collection and were probably obtained from the same site, although given the lack of archaeological context, this cannot be demonstrated. Finally, an important result is that putative migration between eastern and western islands is not observed, as no individual from either region shares IBD with the other cluster.

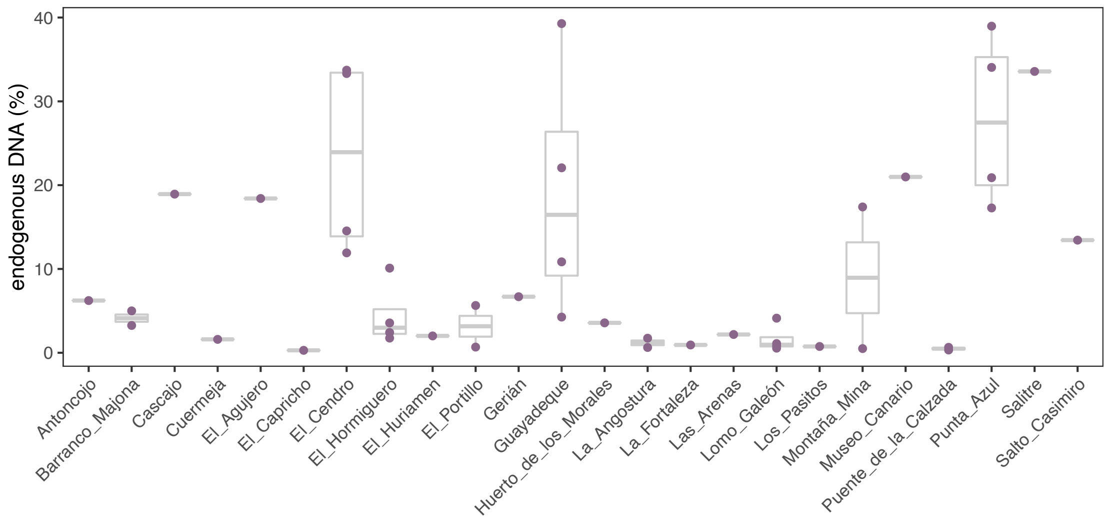

Supplementary Fig. 1. Comparison of endogenous DNA rates between archaeological sites. Sample sizes for each archaeological site are as follows: Antoncojo (n=1), Barranco Majona (n=2), Cascajo (n=1), Cuermeja (n=1), El Agujero (n=1), El Capricho (n=1), El Cendro (n=4), El Hormiguero (n=4), El Huriamen (n=1), El Portillo (n=2), Gerián (n=1), Guayadeque (n=4), Huerto de los Morales (n=1), La Angostura (n=2), La Fortaleza (n=1), Las Arenas (n=1), Lomo Galeón (n=4), Los Pasitos (n=1), Montaña Mina (n=2), Museo Canario (sample from Fuerteventura with no archaeological site adscription; n=1), Puente de La Calzada (n=2), Punta Azul (n=3), Salitre (n=1), Salto Casimiro (n=1). The box represents the interquartile range (25 – 75 percentiles), the bold line is the median and the whiskers represent the minimum and maximum values observed for each archaeological site. Each individual value is shown in violet dots.

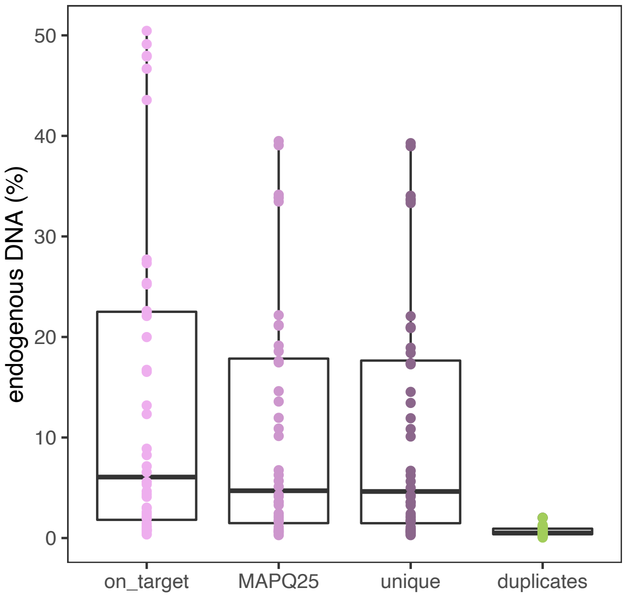

Supplementary Fig. 2. Endogenous DNA and duplicate rates for all individuals considered in the study. Results for the 44 individuals considered (40 new, 4 from[71]). The box represents the interquartile range (25 – 75 percentiles), the bold line is the median and the whiskers represent the minimum and maximum values observed for each filtering step. Each individual value is shown in colored dots.

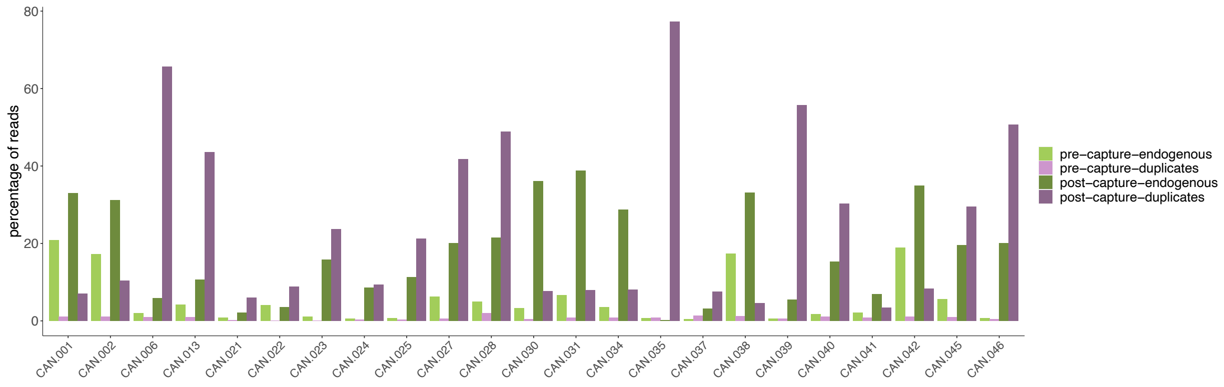

Supplementary Fig. 3. Comparison of % endogenous content in pre-capture and post-capture sequencing data of libraries captured using WISC.

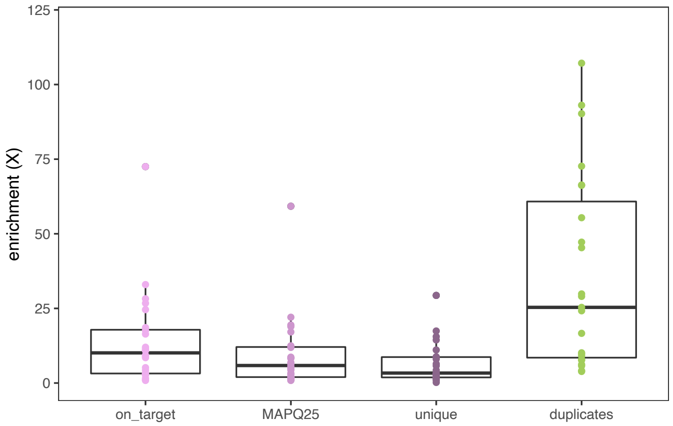

Supplementary Fig. 4. Comparison of WISC performance in all individuals considered in the study (n=23). Note: outlier duplicate rate value for individual CAN.023 was removed for clarity. The box represents the interquartile range (25 – 75 percentiles), the bold line is the median and the whiskers represent the minimum and maximum values observed for each filtering step. Each individual value is shown in colored dots.

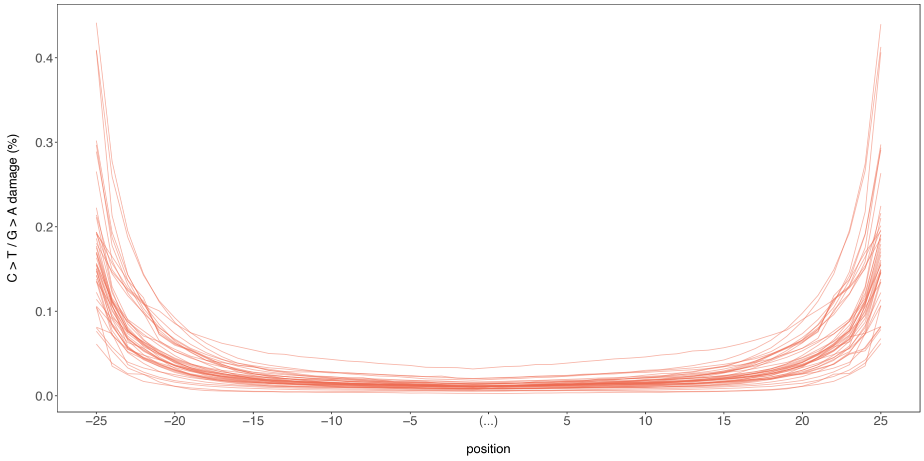

Supplementary Fig. 5. Deamination damage distribution along reads.

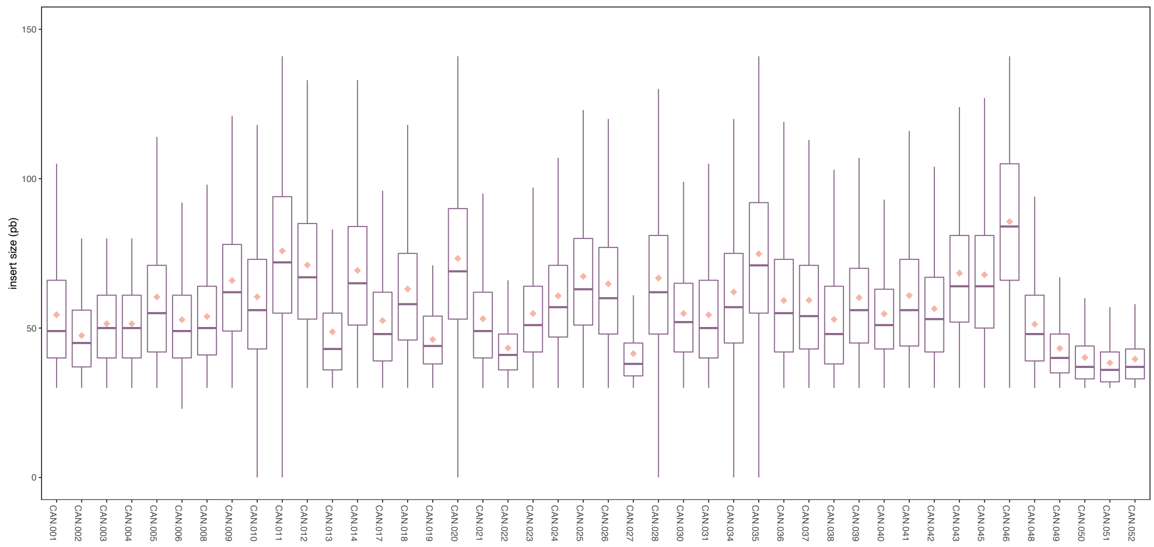

Supplementary Fig. 6. Insert size for all individuals. The box represents the interquartile range (25 – 75 percentiles), the bold line is the median and the orange diamonds are the mean. The whiskers represent the minimum and maximum values observed for each sample.

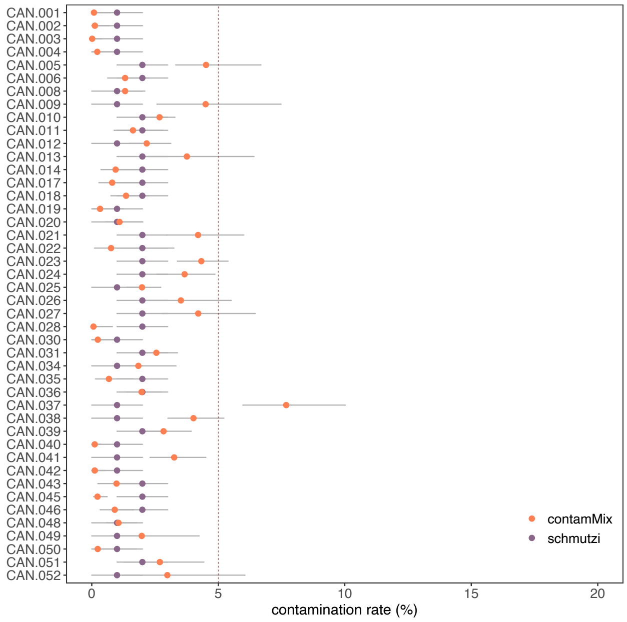

Supplementary Fig. 7. Contamination rates estimated using contamMix and Schmutzi software. For each of the 44 individuals, dots represent the mean value of the contamination estimation obtained, while error bars represent their lower and higher values of the 95% confidence interval.

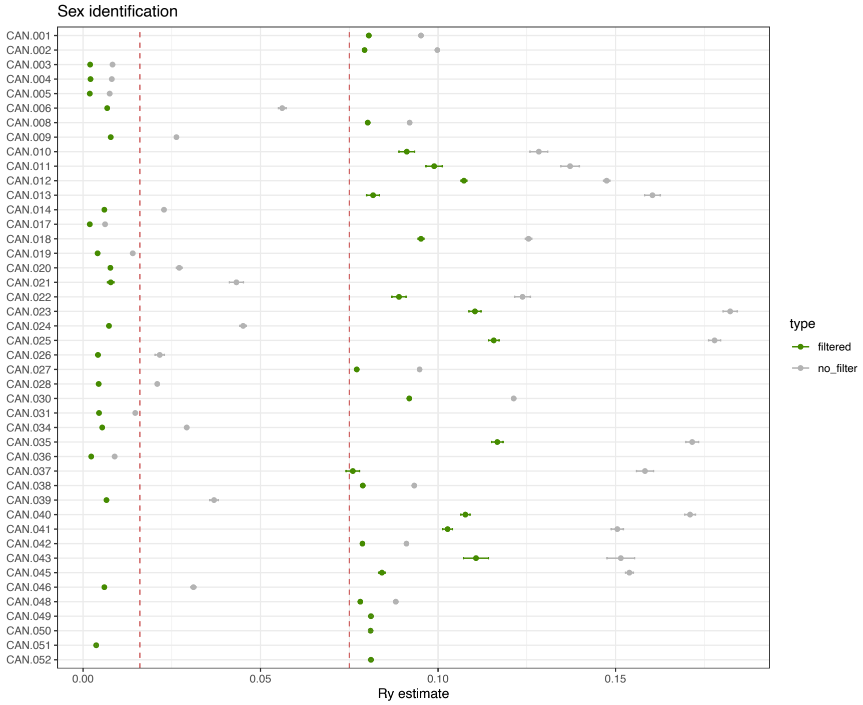

Supplementary Fig. 8. Molecular sex determination using the ry estimator, comparing values obtained before and after pseudo-autosomal and repetitive regions filtering. For each of the 44 individuals, dots represent the mean value of the ry estimate, while error bars represent their lower and upper bound of the 95% confidence interval.

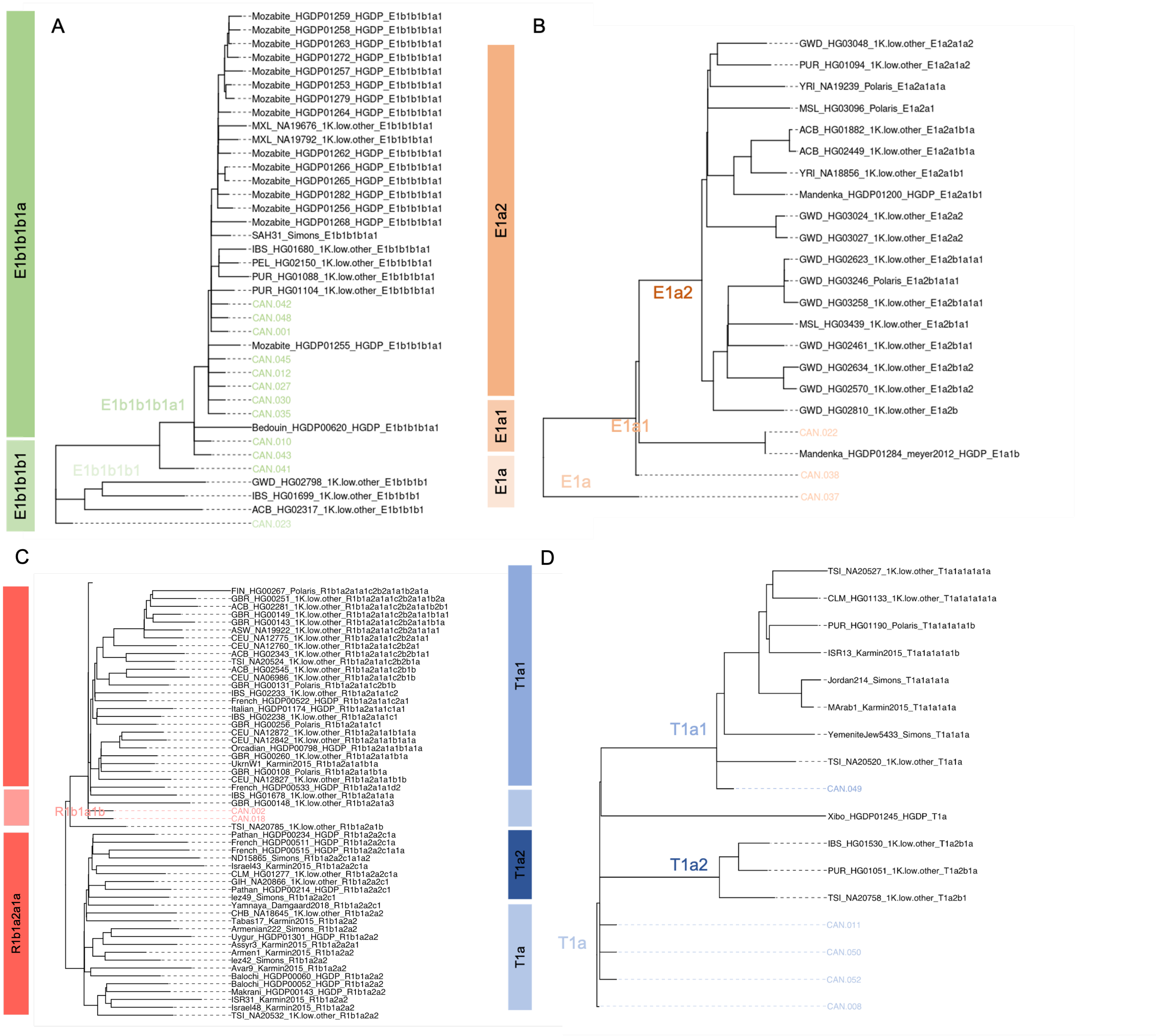

Supplementary Fig. 9. PathPhynder placement of the indigenous individuals within the Y-chromosome tree based on the 1000 Genomes database. (A) E-M183, (B) E-M33, (C) R-M269 and (D) T-M184 clades. CAN.008, CAN.025 and CAN.040 have been excluded from the plots as they were assigned outside the sub-haplogroups considered here.

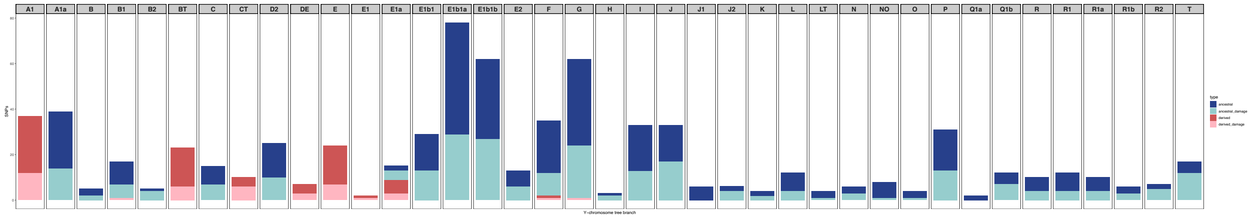

Supplementary Fig. 10. Y-chromosome plot for CAN.038 (E-M33).

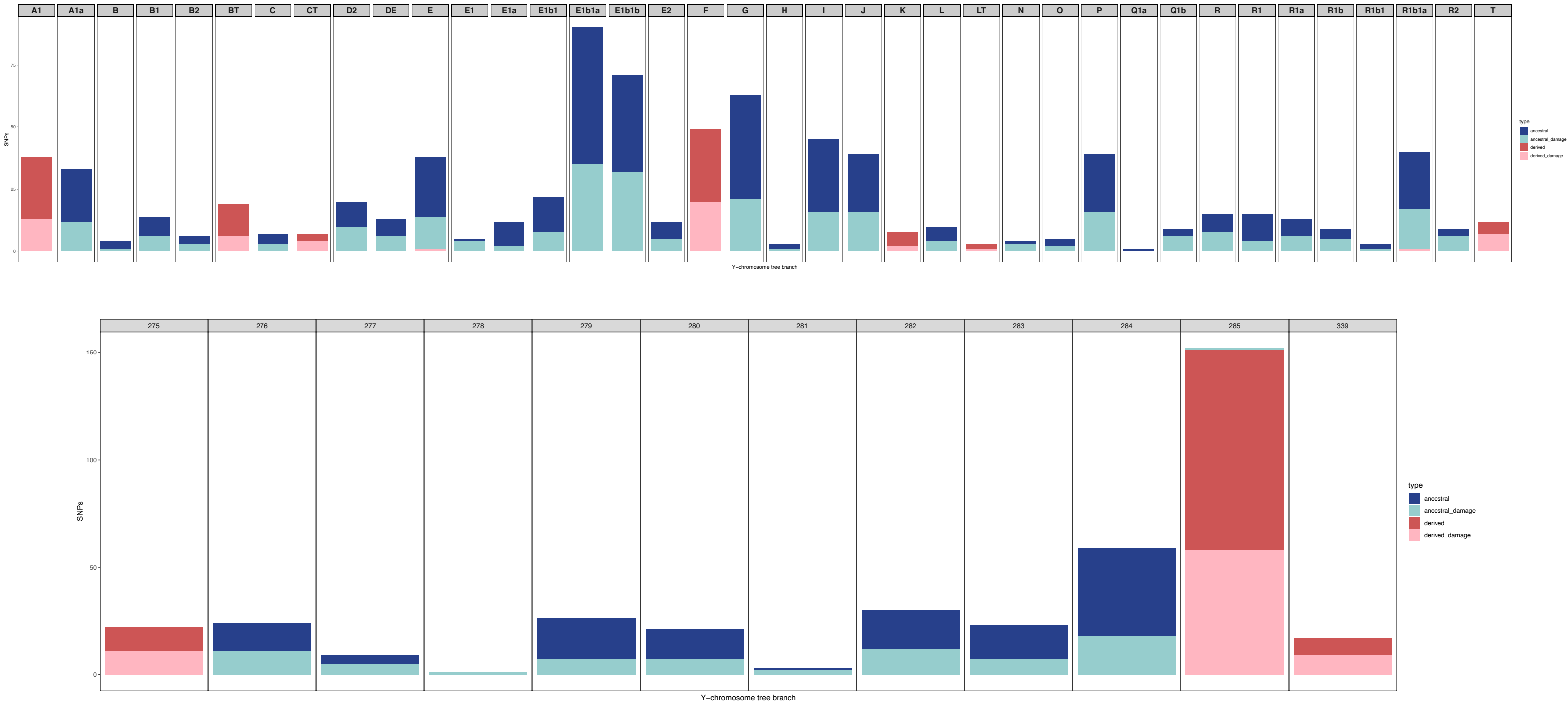

Supplementary Fig. 11. Y-chromosome plot for CAN.008 (above). Below is shown the SNPs covering each of the branches of the T-M184 haplogroup.

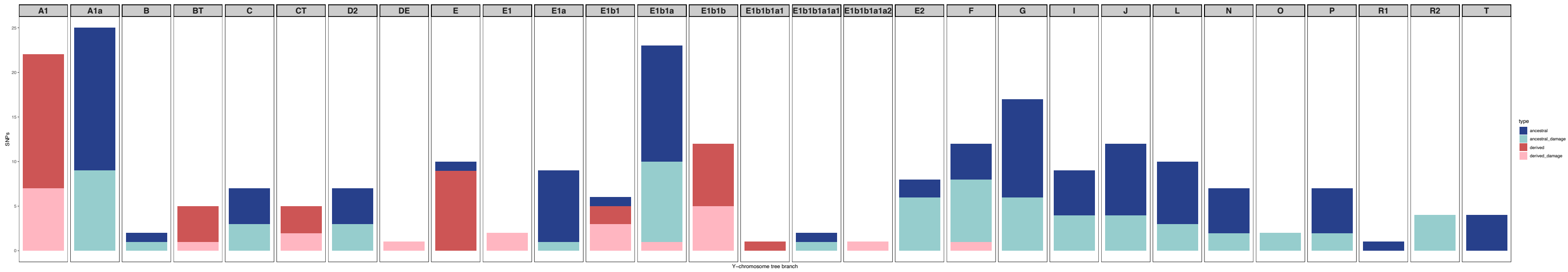

Supplementary Fig. 12. Y-chromosome plot for CAN.040 (E-M78).

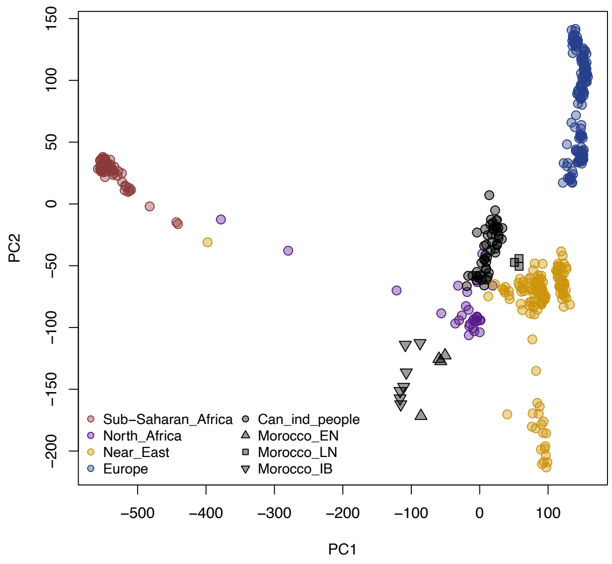

Supplementary Fig. 13. PCA of HGDP-MEGA panel with the indigenous population of the Canary Islands and other ancient individuals from North Africa projected using LASER.

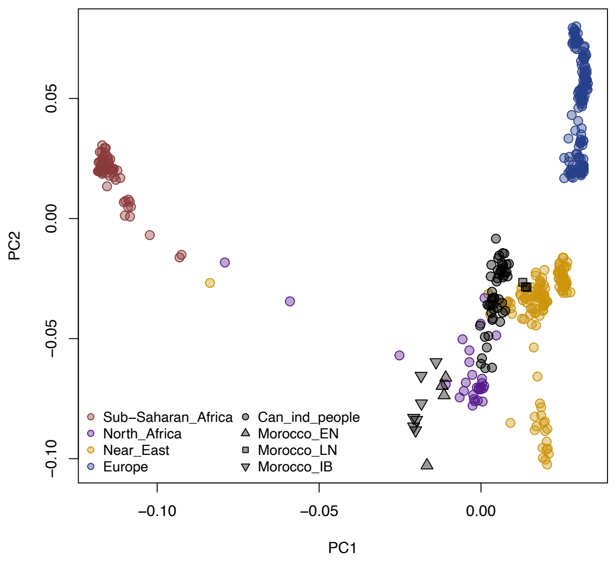

Supplementary Fig. 14. PCA of HGDP-MEGA panel with the indigenous population of the Canary Islands and other ancient individuals from North Africa projected using lsqproject.

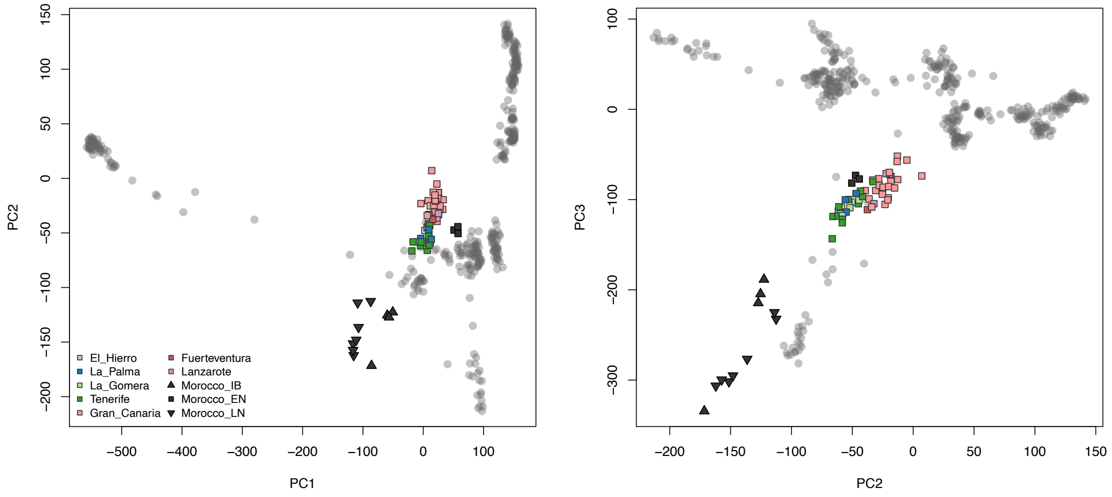

Supplementary Fig. 15. PCA of HGDP-MEGA panel with the indigenous population of the Canary Islands and other ancient individuals from North Africa projected using LASER (PC1/PC2 left; PC2/PC3 right), where the differentiation between western (green/blue) and eastern islands (red/violet) can be observed.

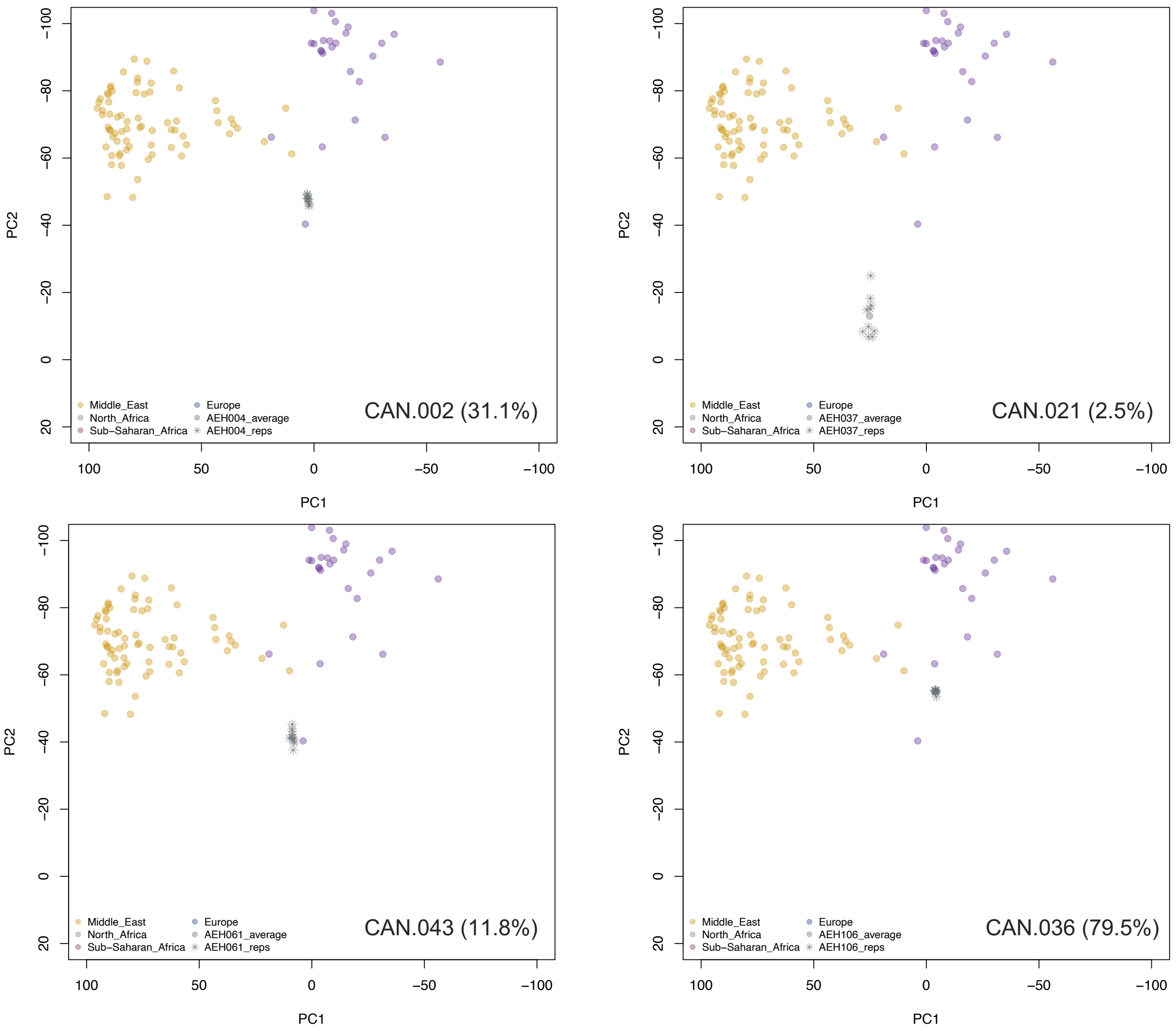

Supplementary Fig. 16. Replicates of the HGDP-MEGA PCA for four ancient individuals of our dataset. Coordinates for the 10 replicates are indicated with grey asterisks and average value is indicated with a black circle. ID and coverage percentage is indicated for each individual.

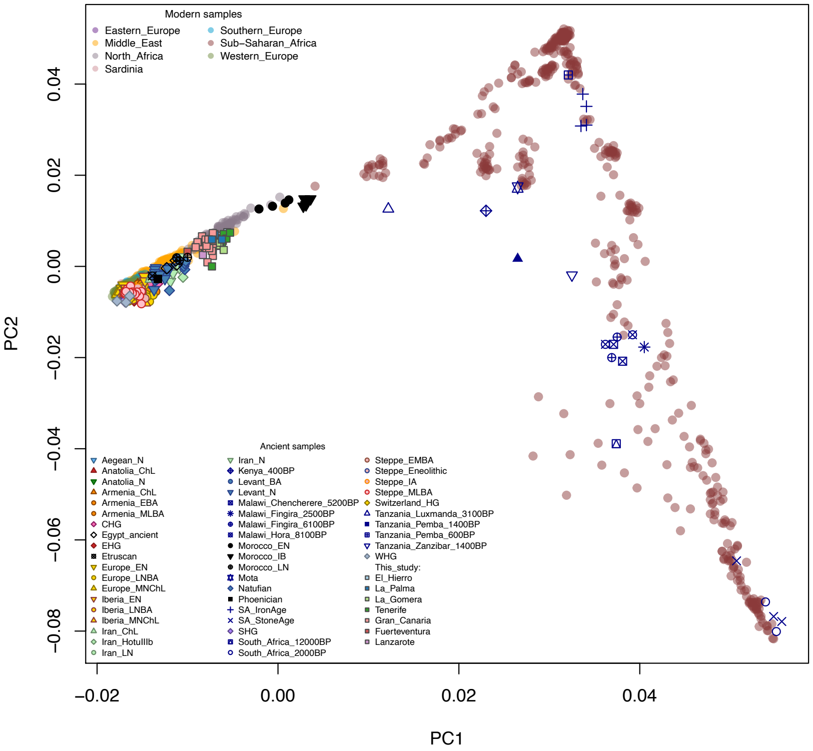

Supplementary Fig. 17. PCA of the Human Origins panel with the indigenous population of the Canary Islands and other ancient and modern individuals from Europe, the Middle East and Africa (PC1 vs. PC2).

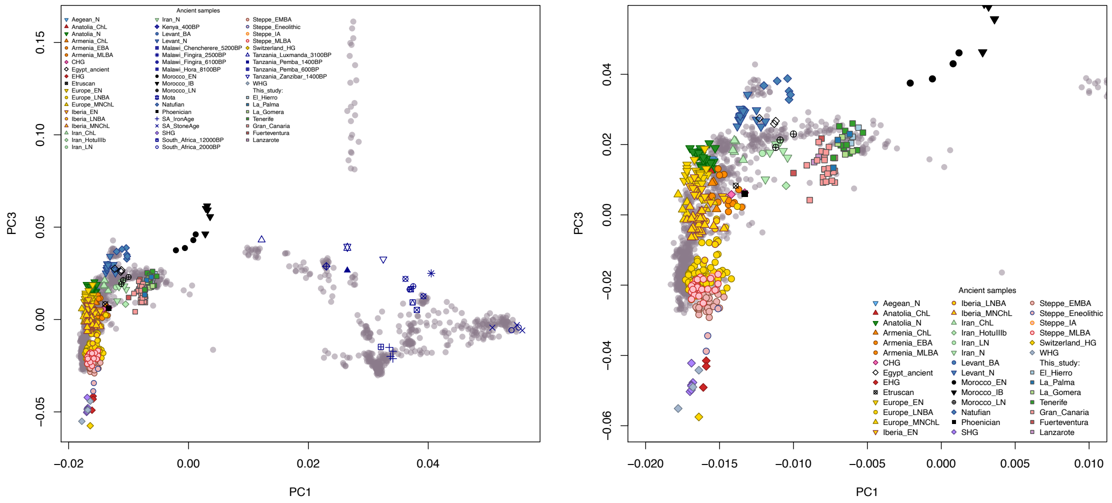

Supplementary Fig. 18. PCA of the Human Origins panel with the indigenous population of the Canary Islands and other ancient and modern individuals from Europe, the Middle East and Africa (PC1 vs. PC3). The right panel shows a close up on the Eurasian populations.

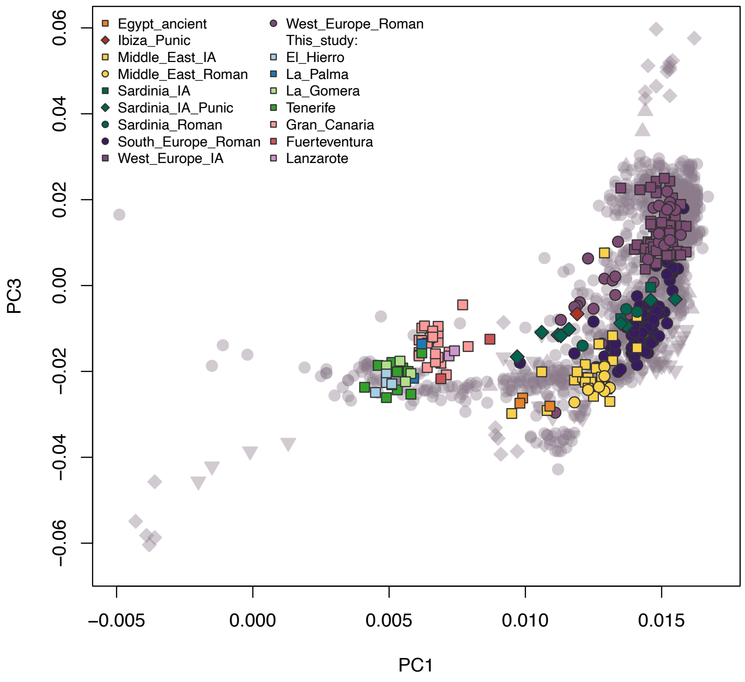

Supplementary Fig. 19. PCA of the Human Origins panel with the indigenous population of the Canary Islands and other ancient individuals from the Iron Age of Europe and the Middle East.

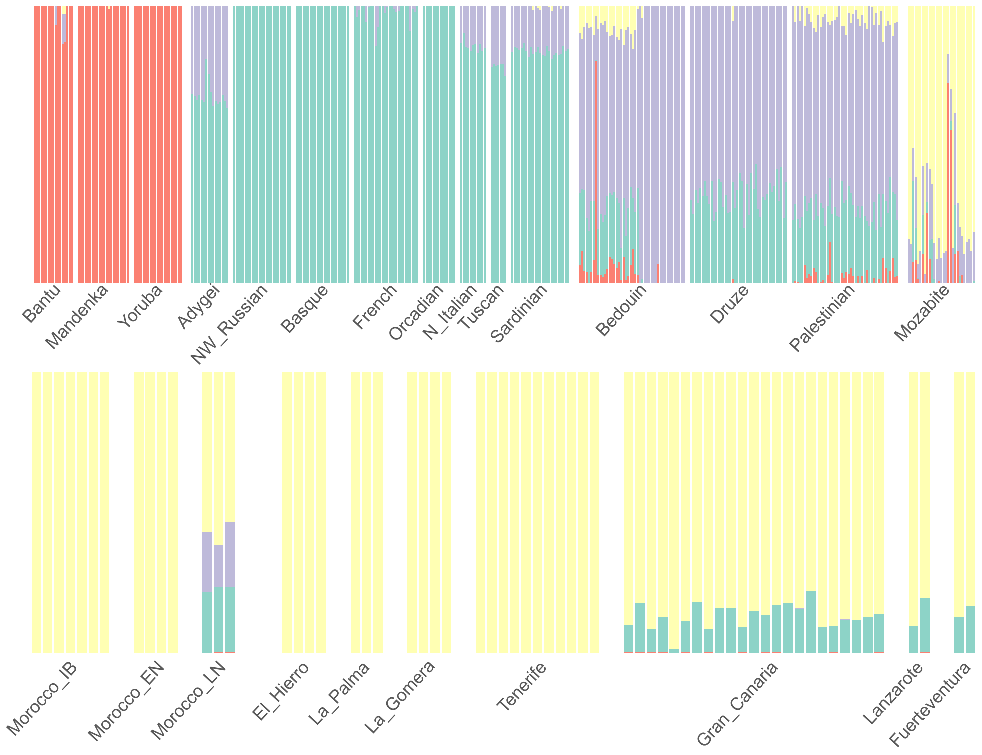

Supplementary Fig. 20. Unsupervised clustering analysis (*K*=4) of the modern populations of the HGDP-MEGA panel (upper panel) and the indigenous population of the Canary Islands and other ancient individuals from North Africa (lower panel).

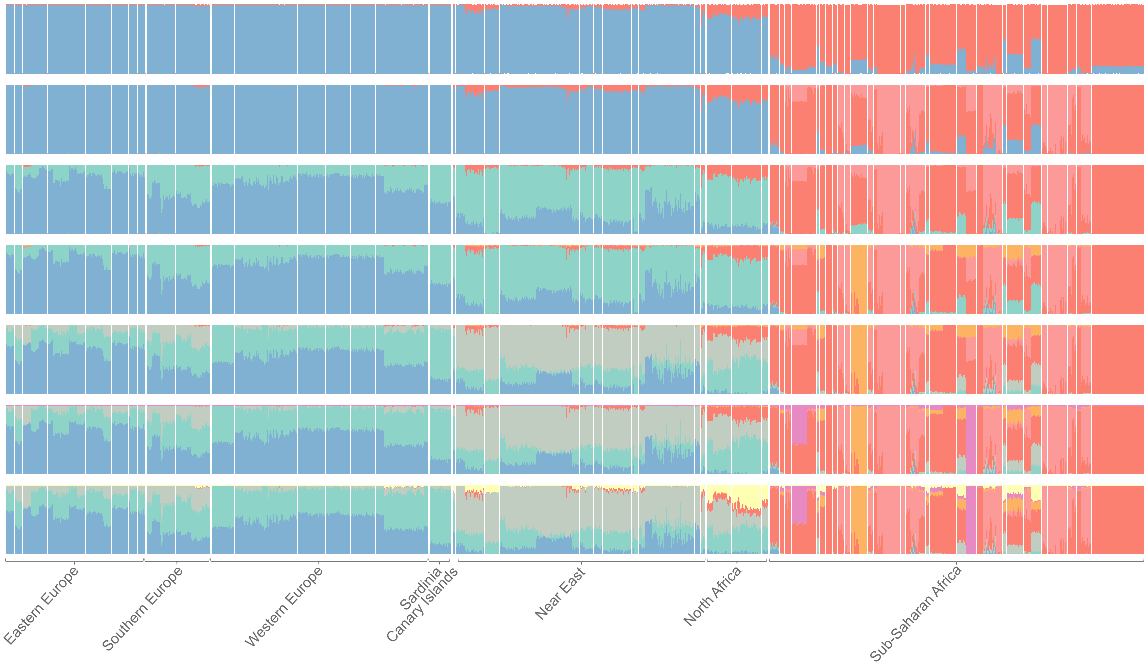

Supplementary Fig. 21. Unsupervised clustering analysis of the modern populations of the Human Origins panel including individuals from Europe, the Near East, North African and Sub-Saharan Africa from *K*=2 to *K*=8.

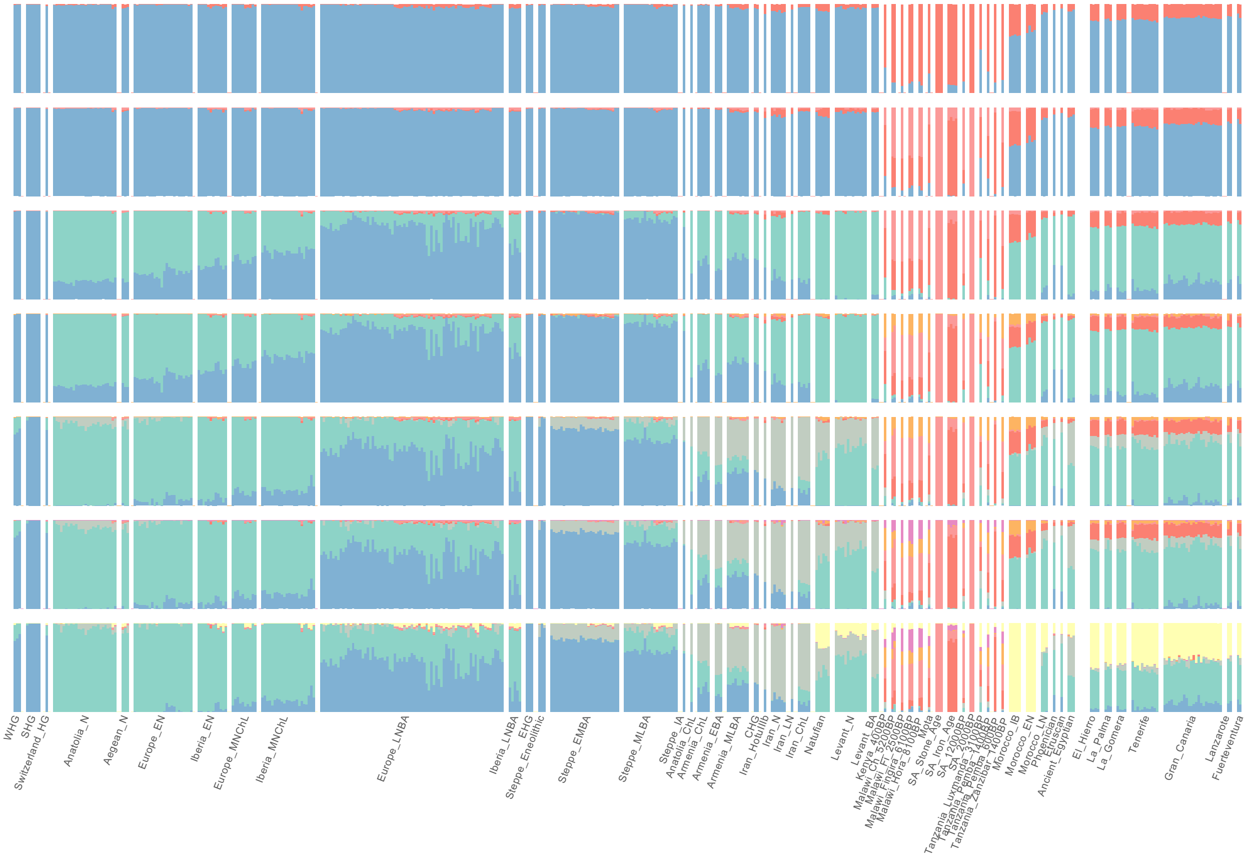

Supplementary Fig. 22. Unsupervised clustering analysis of the ancient populations of the Human Origins panel as described in Section 5.3.2 and the indigenous people of the Canary Islands from *K*=2 to *K*=8.

5

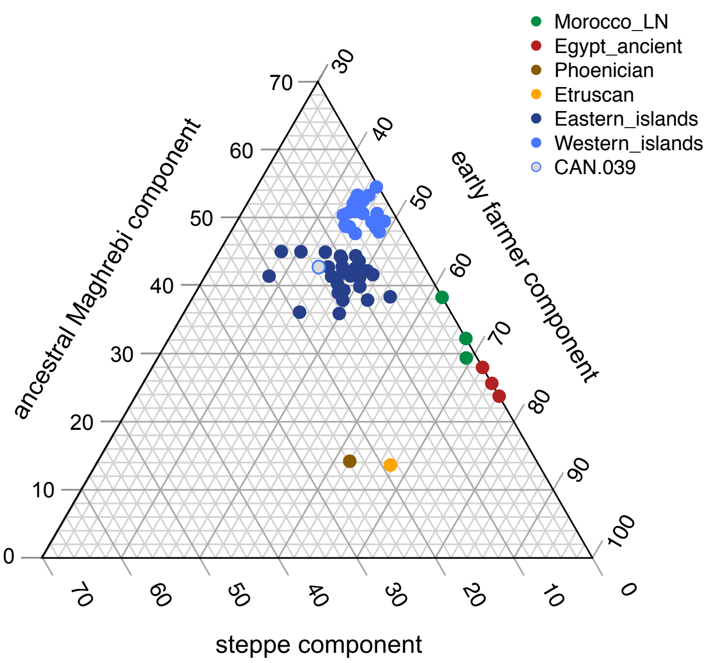

Supplementary Fig. 23. Ternary plot showing the proportion of ancestral Maghrebi, European early farmer and Steppe components on the Canarian indigenous people and other ancient populations, based on ADMIXTURE results for *K*=8.

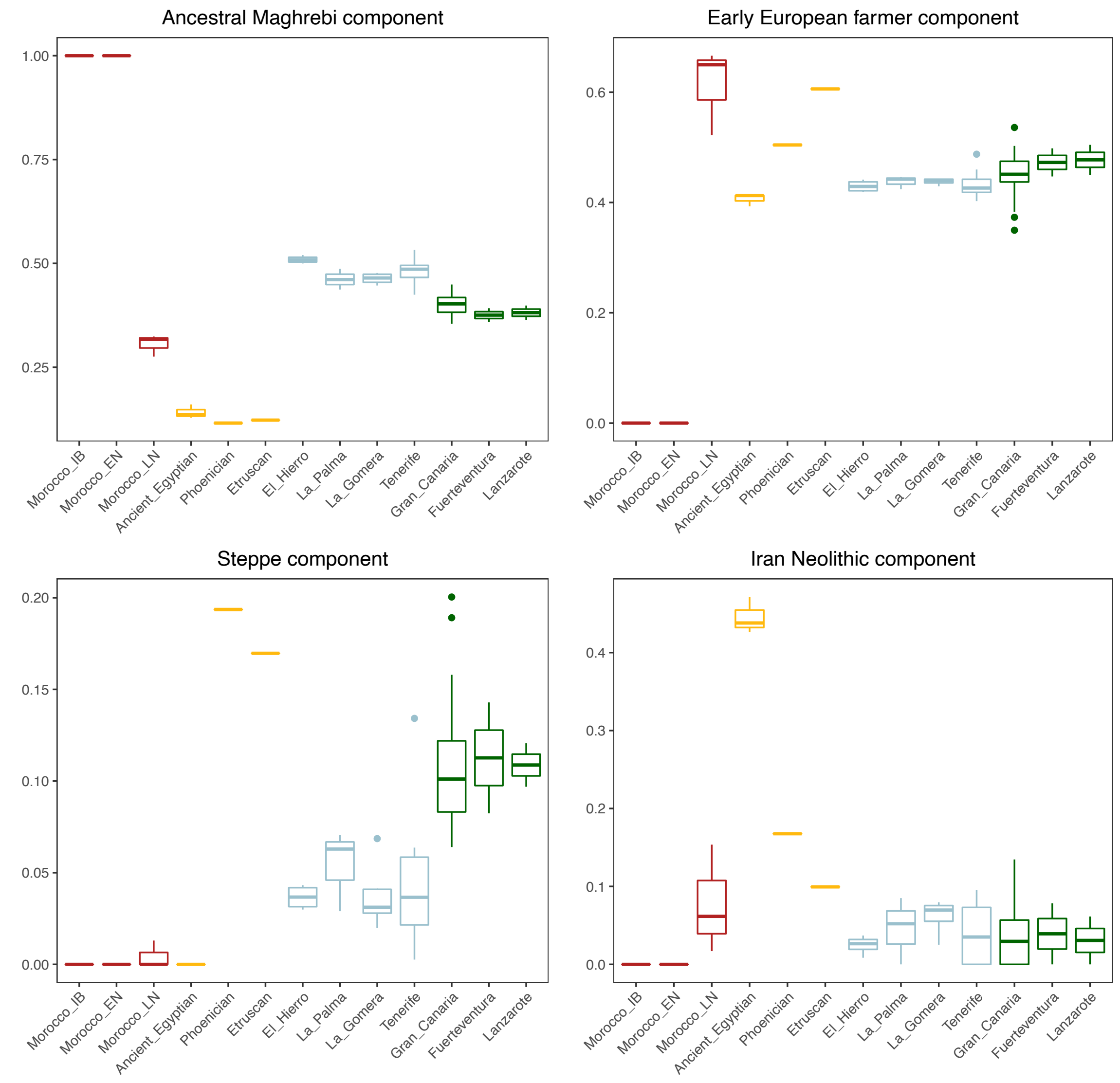

Supplementary Fig. 24. Contribution from the ancestral Maghrebi, European early farmer and the Iran Neolithic and Steppe components on the Canarian indigenous people and other ancient populations, based on ADMIXTURE results for *K*=8. Populations include: Morocco_IB (n=5), Morocco_EN (n=4), Morocco_LN (n=3), Phoenician (n=1), Etruscan (n=1), Ancient_Egyptian (n=3), El Hierro (n=4), La Palma (n=3), La Gomera (n=4), Tenerife (n=11), Gran Canaria (n=23), Lanzarote (n=2), Fuerteventura (n=2). The box represents the interquartile range (25 – 75 percentiles), the bold line is the median and the whiskers represent the minimum and maximum values observed for each population. Outlier values are shown in colored dots.

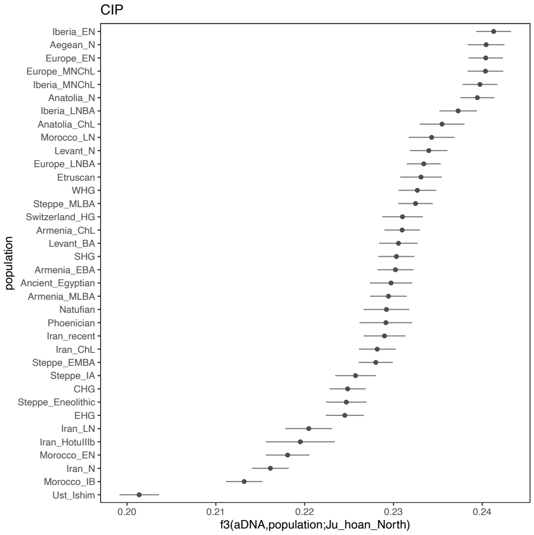

Supplementary Fig. 25. Outgroup *f3*-statistic results for the indigenous population of the Canary Islands (n = 49). Dots represent the mean of the *f3*-statistics results and error bars represent the standard errors.

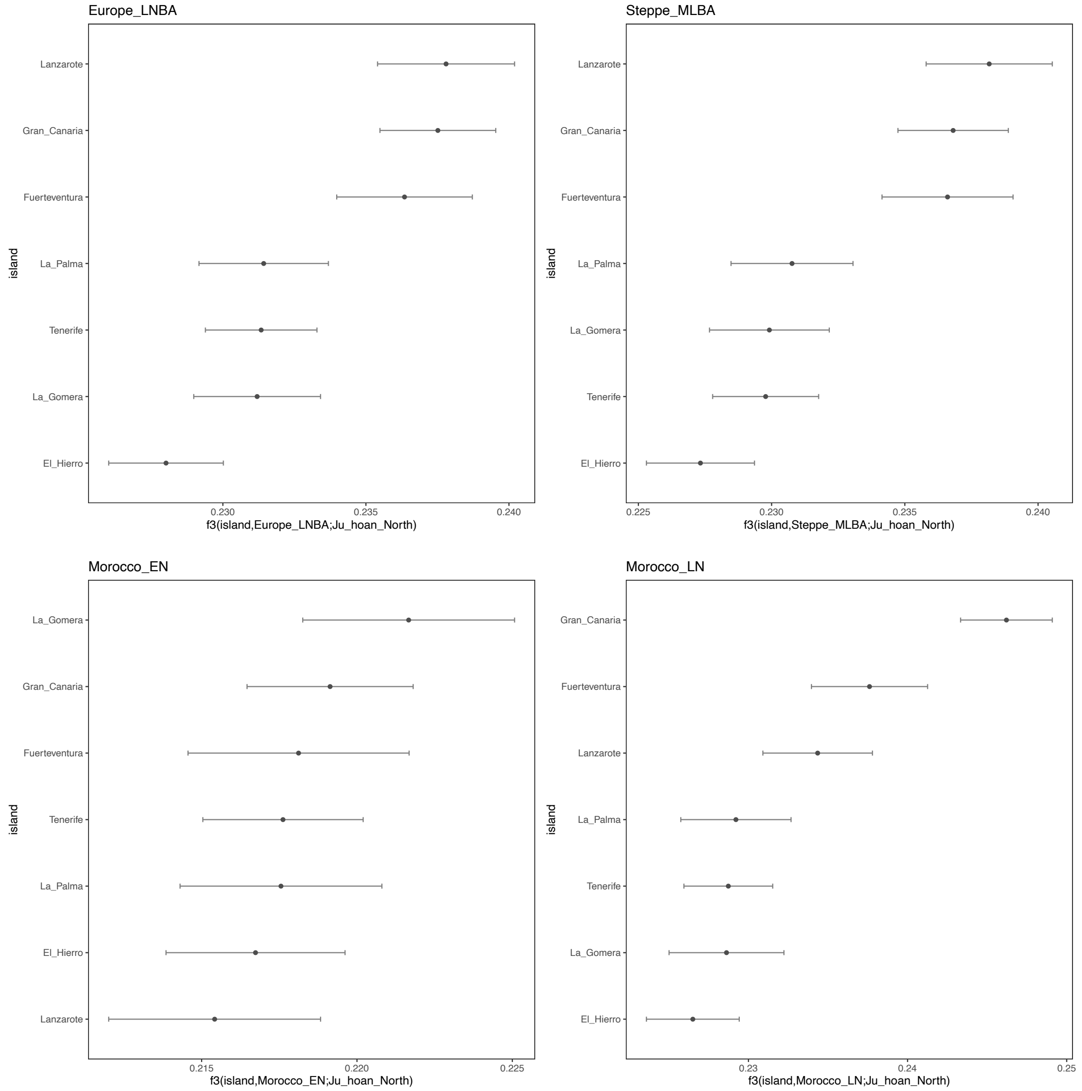

Supplementary Fig. 26. Outgroup *f3*-statistic results for the islands populations when compared to Europe_LNBA, Steppe_MLBA, Morocco_EN and Morocco_LN. Dots represent the mean of the *f3*-statistics and error bars represent the standard errors. Analyses performed using El Hierro (n=4), La Palma (n=3), La Gomera (n=4), Tenerife (n=11), Gran Canaria (n=23), Lanzarote (n=2), Fuerteventura (n=2).

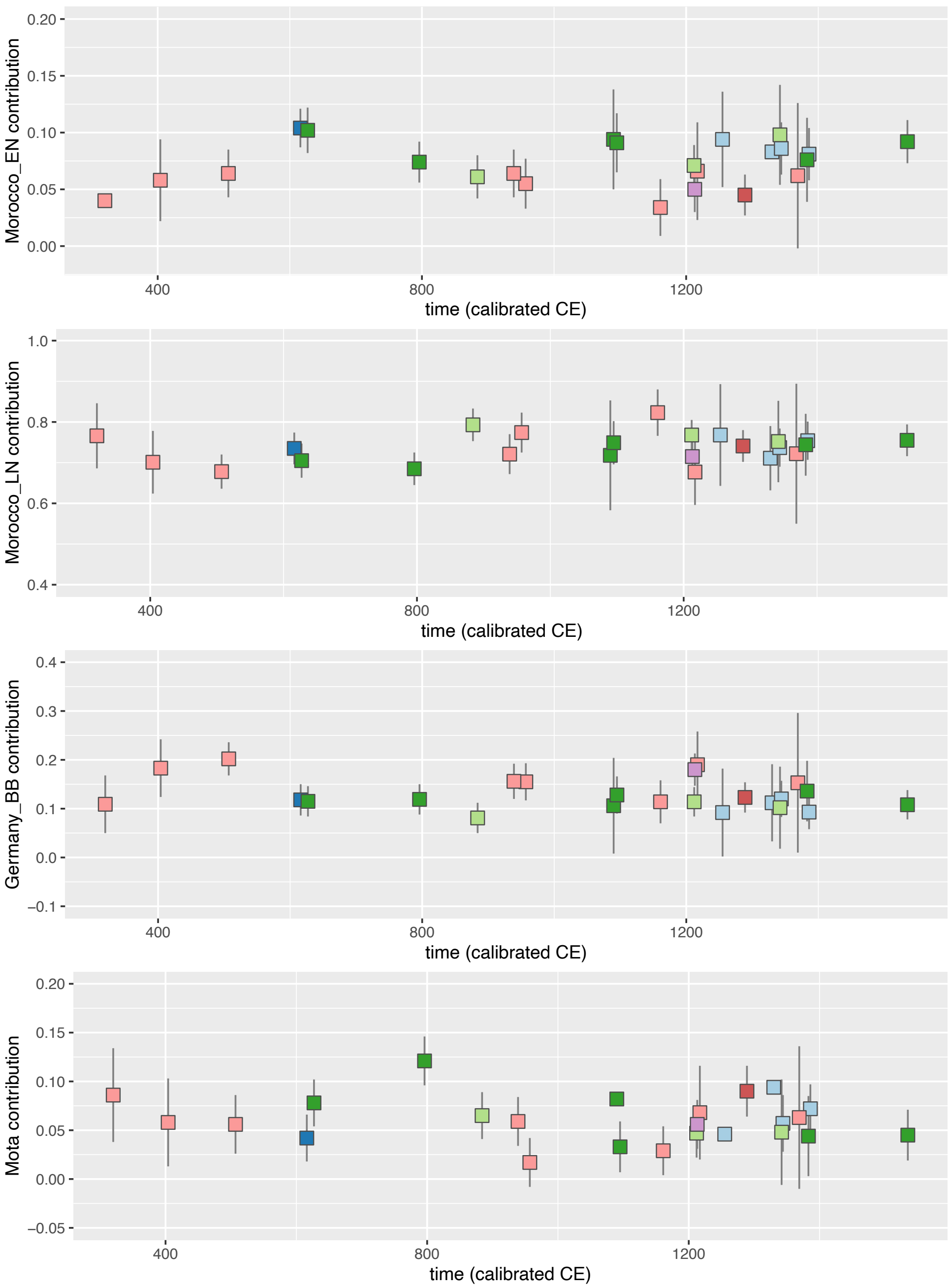

Supplementary Fig. 27. Contributions for the 4-stream model at individual level over time. Analysis performed using four individuals from El Hierro, three individuals from La Palma; four individuals from La Gomera, 11 individuals from Tenerife, 23 individuals from Gran Canaria, two individuals from Lanzarote, and two from Fuerteventura. Color code for islands is as in Supplementary Fig. 15. The squares represent the estimated contribution, while the whiskers represent its standard error.

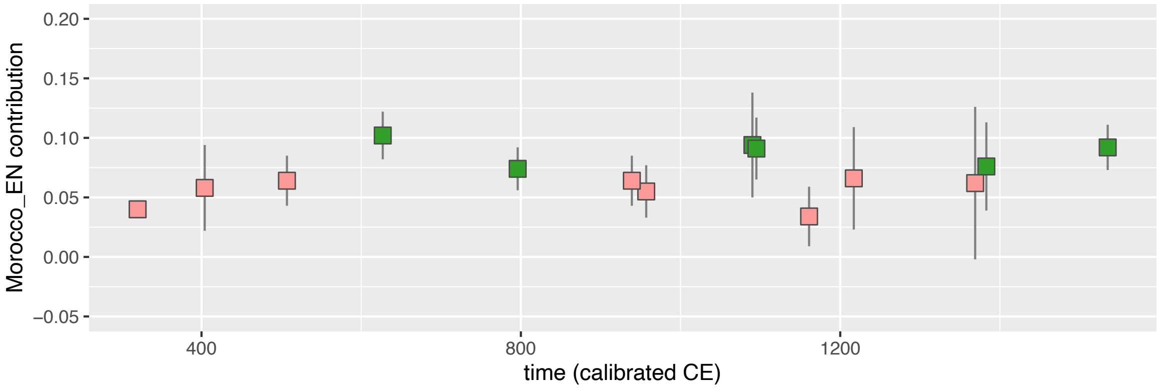

Supplementary Fig. 28. Contributions for the Morocco_EN component in the 4-stream model in individuals from Tenerife (N=11; green) and Gran Canaria (N=23; pink) over time. The squares represent the estimated contribution, while the whiskers represent its standard error.

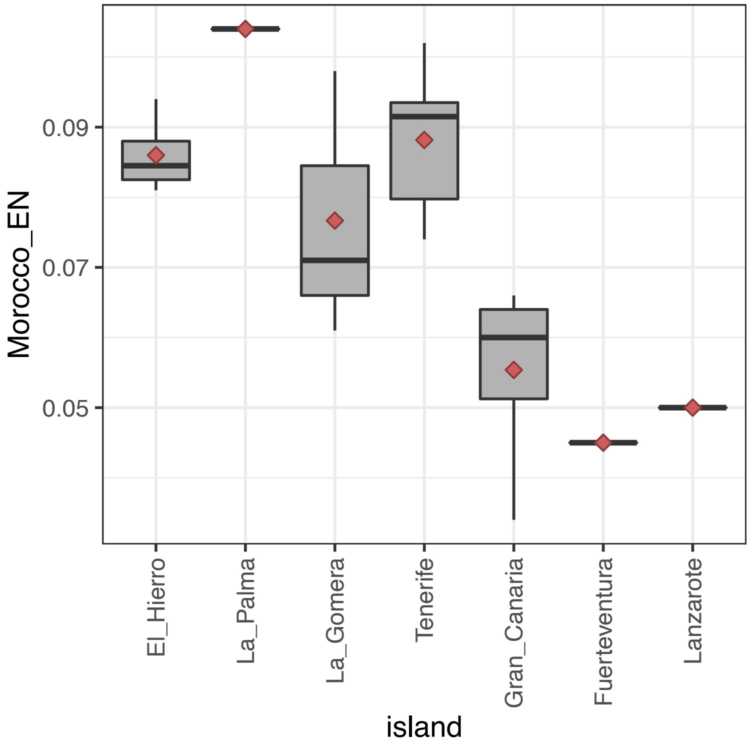

Supplementary Fig. 29. Contributions for the Morocco_EN component in the qpAdm 4-stream model for the sub-sampling test in the different islands. Analysis performed using four individuals from El Hierro, three individuals from La Palma; four individuals from La Gomera, 11 individuals from Tenerife, 23 individuals from Gran Canaria, two individuals from Lanzarote, and two from Fuerteventura. The box represents the interquartile range (25 – 75 percentiles), the bold line is the median and the red diamonds the mean. The whiskers represent the minimum and maximum values observed for each population.

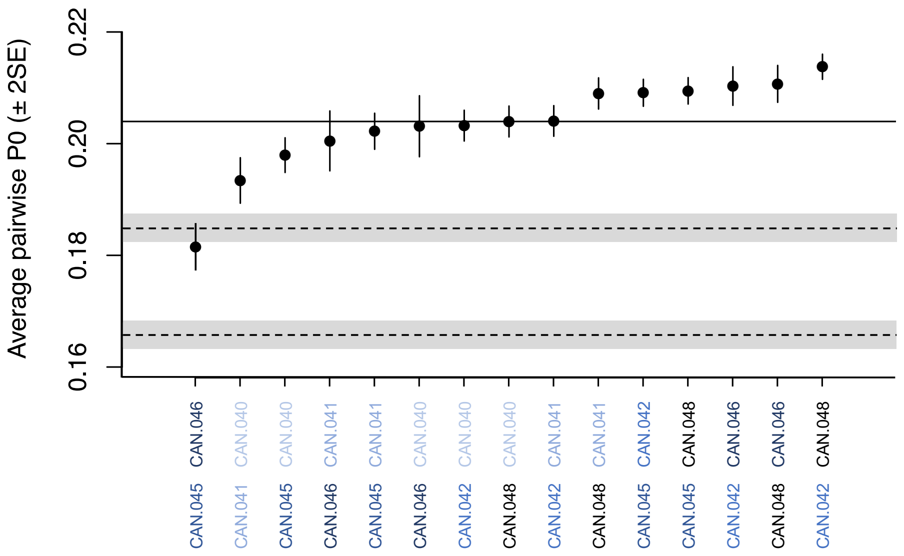

Supplementary Fig. 30. P0 estimations for the indigenous individuals of Tenerife. Each dot represents the average pairwise P0 obtained for each pair of individuals, while the error bars represent the standard error.

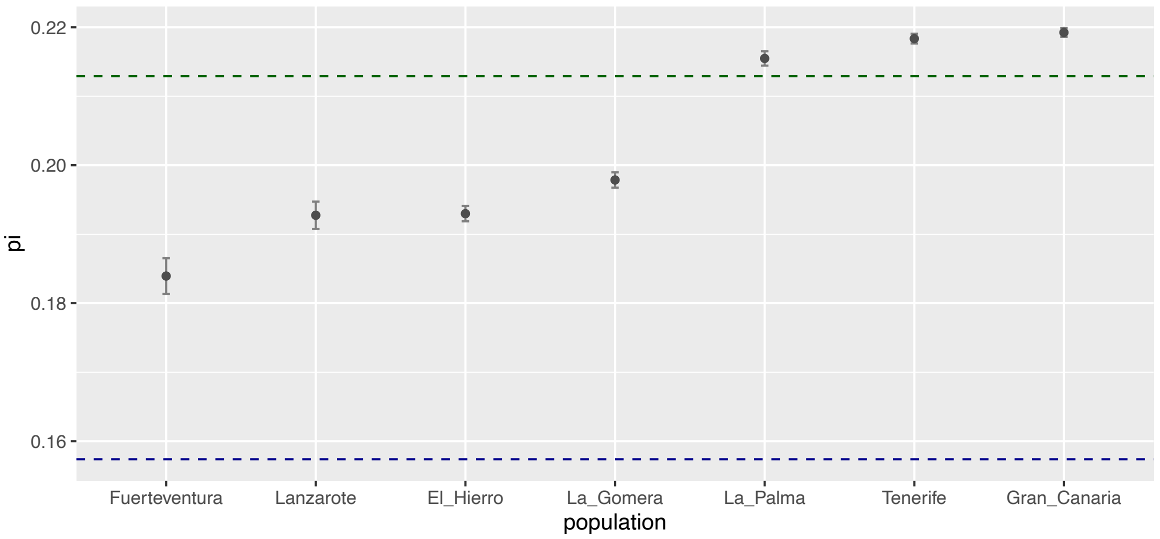

Supplementary Fig. 31. Heterozygosity estimations obtained for all the islands' populations. Each dot represents the average *pi* estimation obtained for each group of individuals, while the error bars represent the standard errors. The analysis was performed using four individuals from El Hierro, three individuals from La Palma; four individuals from La Gomera, 11 individuals from Tenerife, 23 individuals from Gran Canaria, two individuals from Lanzarote, and two from Fuerteventura. The green dotted line represents the *pi* estimation obtained from Morocco_EN (N=4; *pi* = 0.157) and the red dotted line the *pi* estimation from Morocco_LN (N=3; *pi* = 0.213).

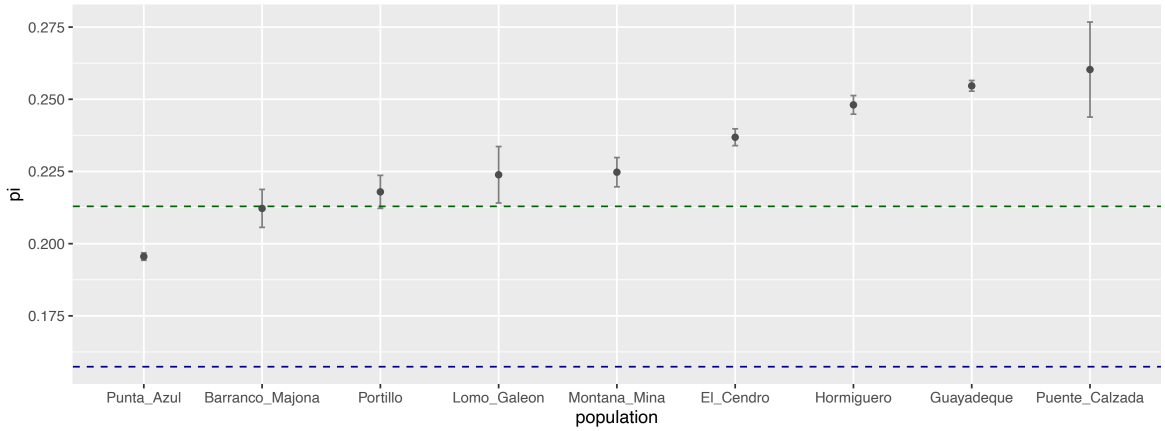

Supplementary Fig. 32. Heterozygosity estimations obtained for all the archaeological sites with at least two individuals. Each dot represents the average *pi* estimation obtained for each group of individuals, while the error bars represent the standard error. The green and red dotted line represents the same values as in Supplementary Figure 31. Sample size is four for Punta Azul, Lomo Galeón, El Cendro, El Hormiguero and Guayadeque, while sample size is two for Barranco Majona, El Portillo, Montaña and Puente de La Calzada.

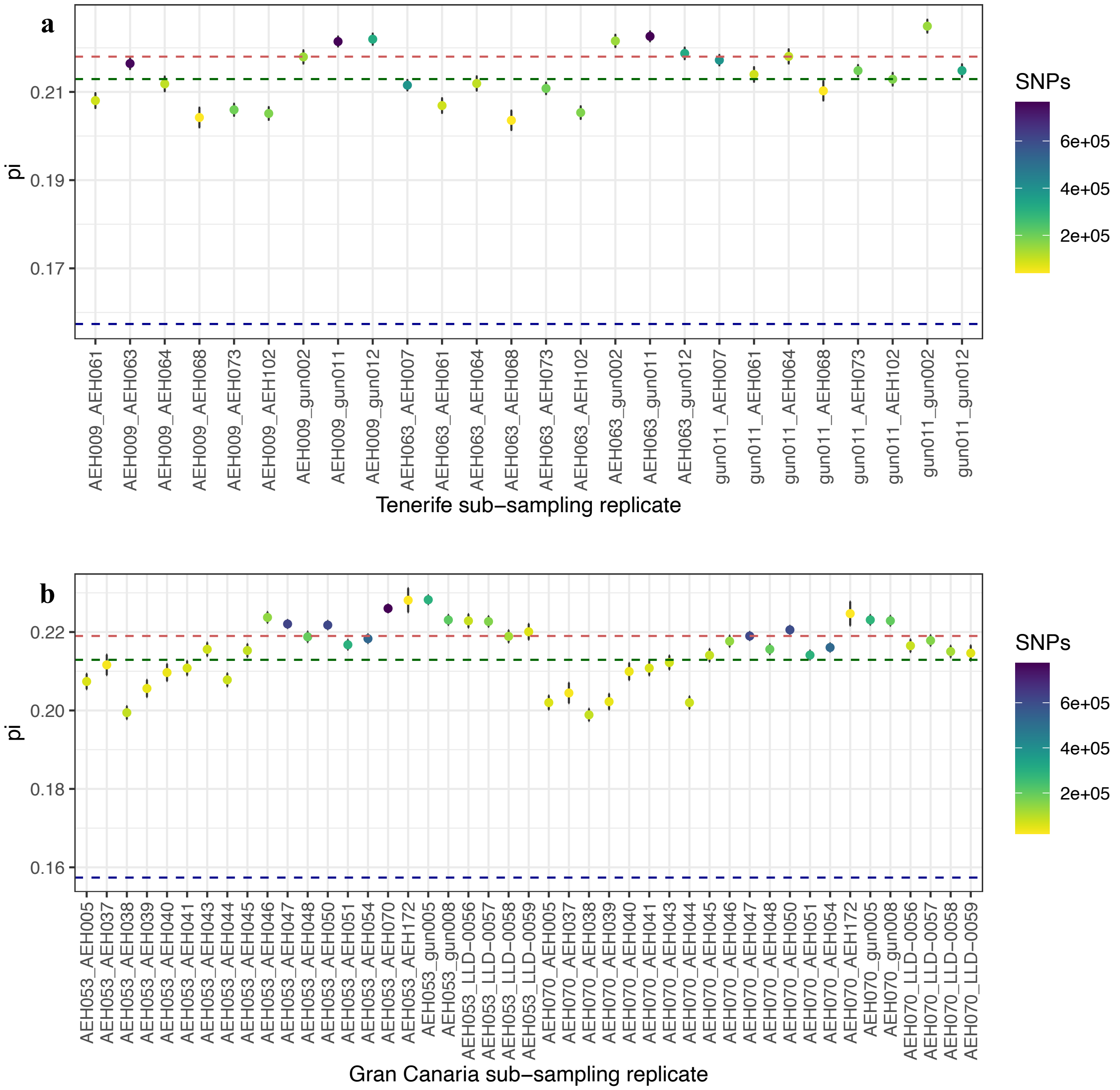

Supplementary Fig. 33. Heterozygosity estimations obtained for all sub-sampling replicates for the islands of Tenerife (a) and Gran Canaria (b). Each dot represents the average *pi* estimation obtained for each sub-sampling replicate, while the error bars represent the standard error. The green and red dotted line represents the same values as in Fig. 3a.

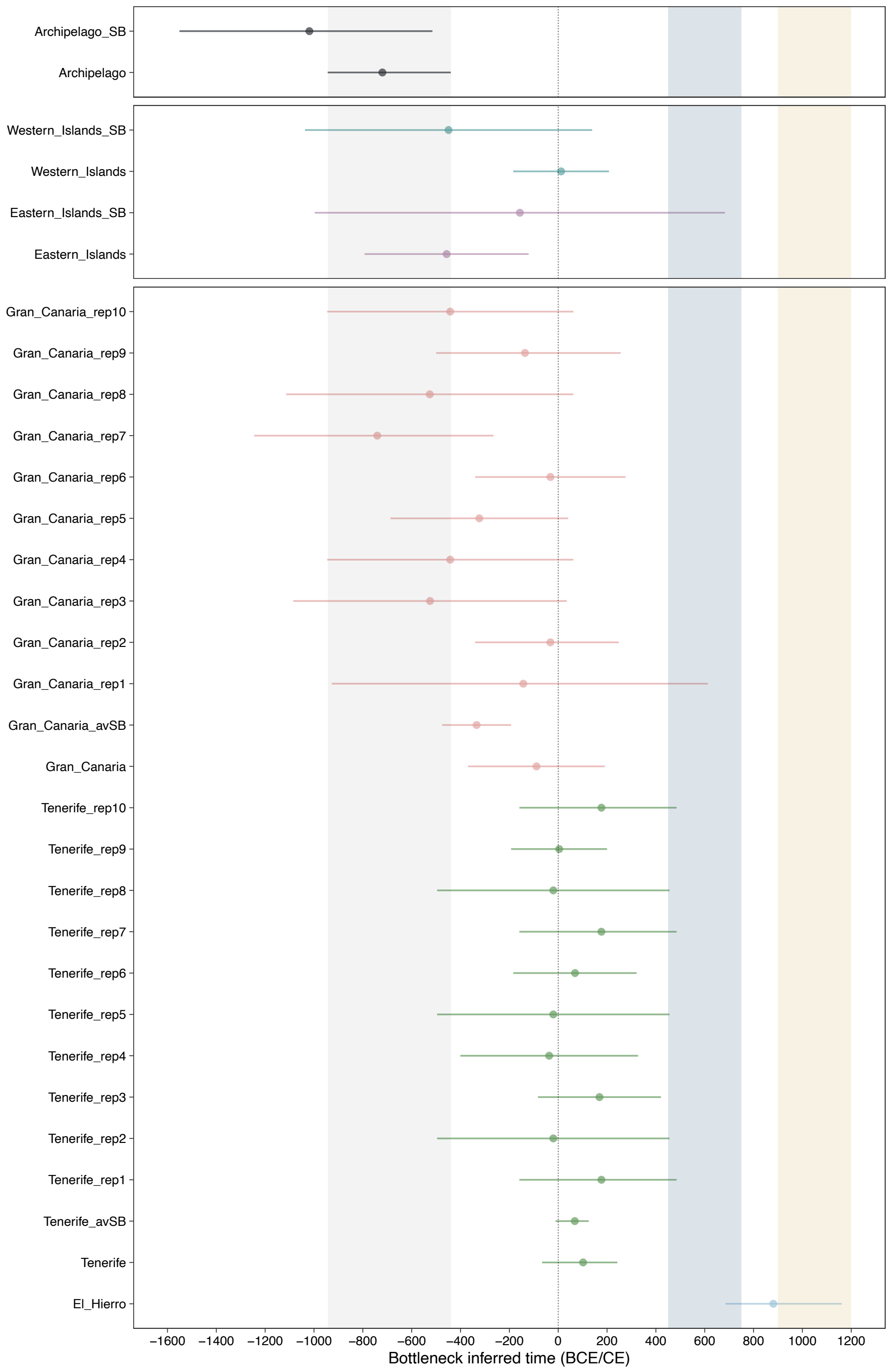

Supplementary Fig. 34. Founder events observed in the indigenous people of the archipelago, eastern, and western islands; Tenerife, Gran Canaria, and El Hierro. For each dataset we evaluated the putative effects of the sampling bias (_SB; N=10 in each replicate) and included in the plot mean values of the founder event time period (dots) and their associated standard error (error bars). For Gran Canaria and Tenerife, we included the average founder event time for 10 replicates (_rep) with four individuals each and the average time period of the 10 replicates (_avSB). For more information, see Supplementary Note 9.2. The grey tile represents the time range of the putative archipelago's founder event, while the blue tile

represents the Vandal Minimum period range, and the yellow tile represents the Medieval Warm Period.

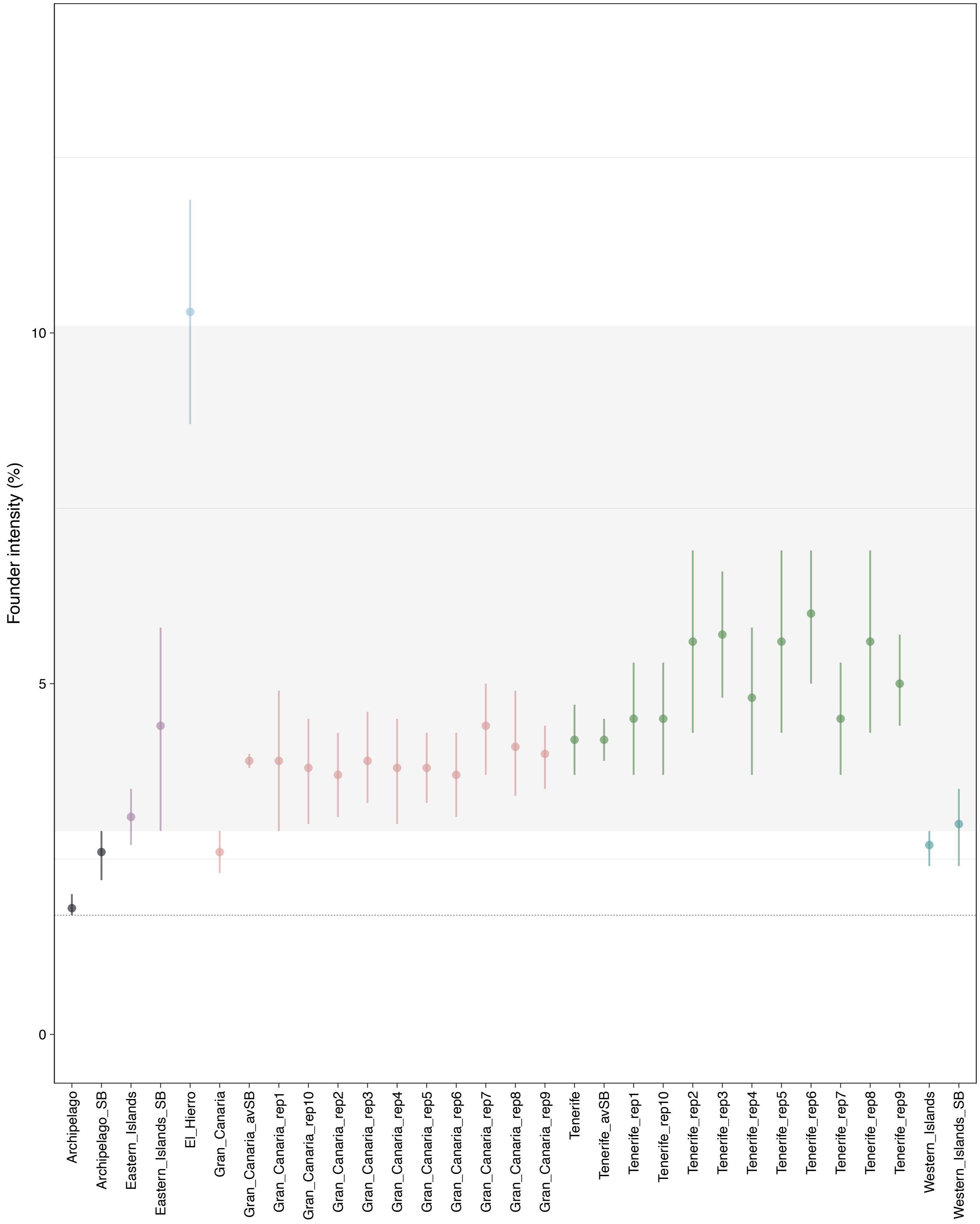

Supplementary Fig. 35. Intensity observed for each founder event studied. For each dataset we evaluated the putative effects of the sampling bias (_SB; N=10 in each replicate) and included in the plot mean values of the founder event intensity (dots) and their associated standard error (error bars). For Gran Canaria and Tenerife, we included the average founder

event intensity for 10 replicates (_rep) including four individuals each and the average intensity of the 10 replicates (_avSB). The grey tile represents the intensity range of most island founder events according to ref. [130], while the dotted line represents the founder intensity of Ashkenazi Jews (~1.7%).

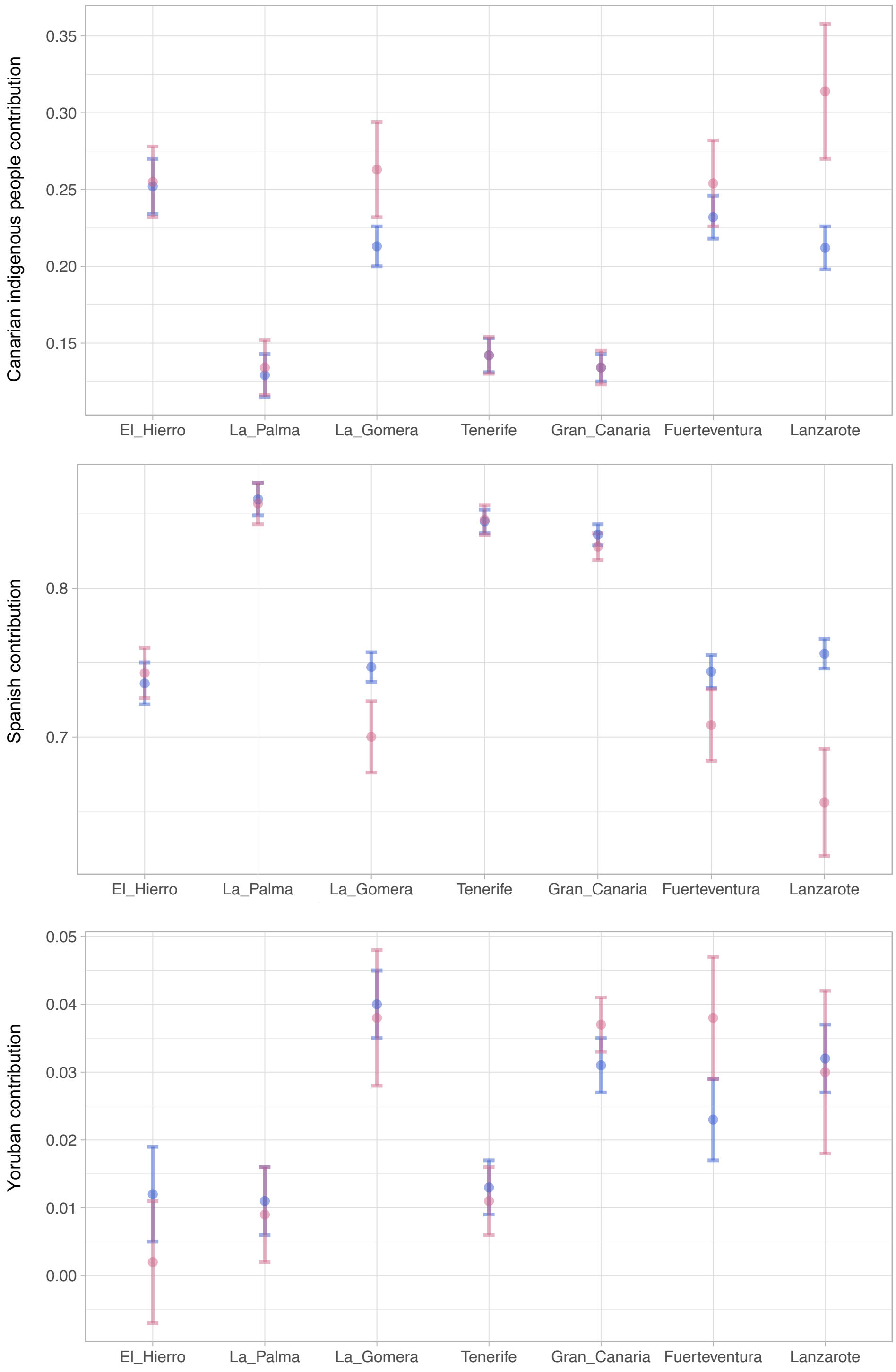

Supplementary Fig. 36. Admixture estimations based on genome-wide data considering the global indigenous people (blue) and each insular indigenous population (red). Each dot represents the average admixture contribution of each population source (Canarian indigenous, Spanish or Yoruban) to each present-day island population, associated to their standard error. This analysis has been performed using 34 present-day individuals from El Hierro; 35 from La

Palma; 78 from La Gomera; 64 from Tenerife; 117 from Gran Canaria; 32 from Fuerteventura and 56 from Lanzarote.

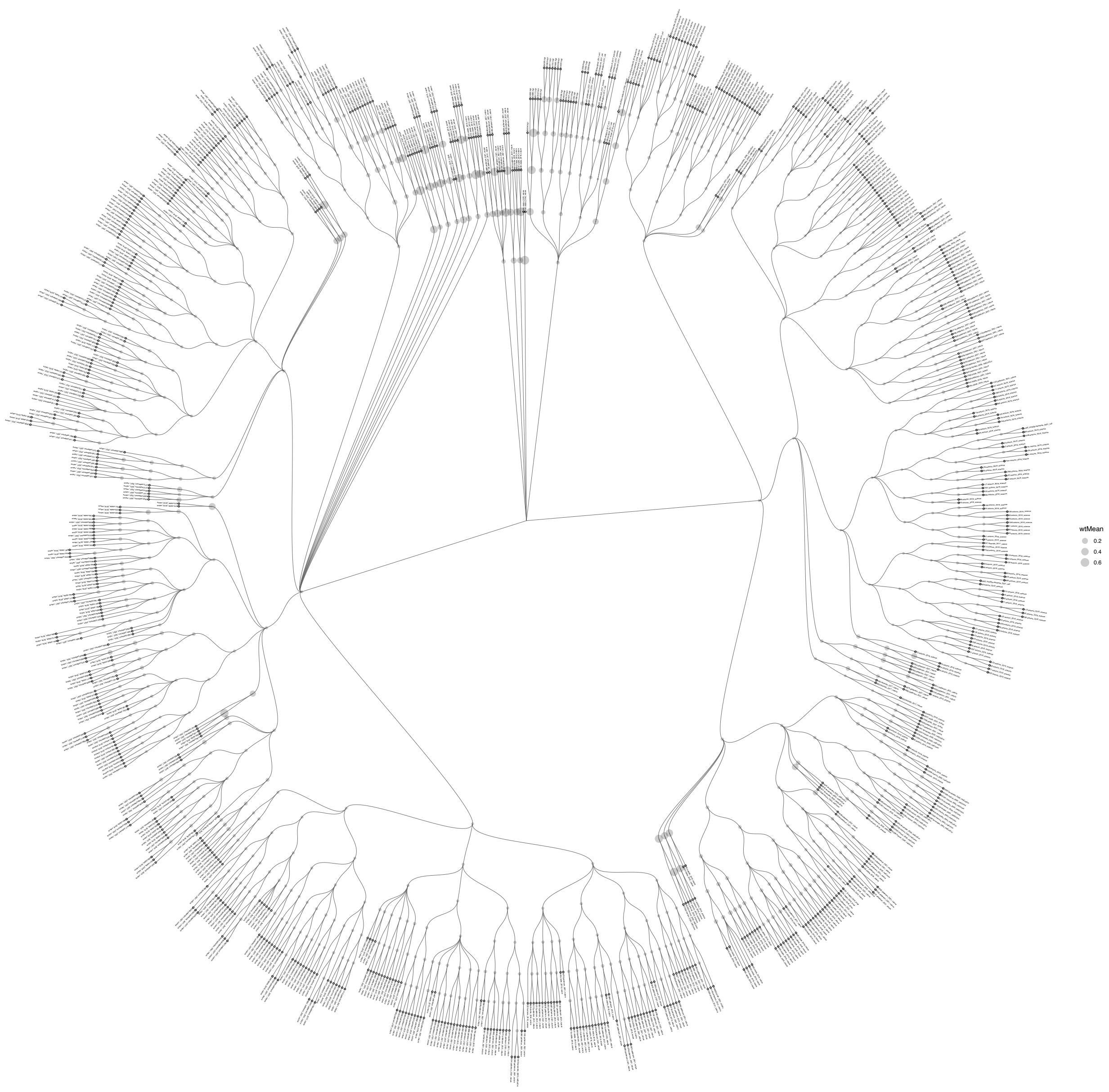

Supplementary Fig. 37. Hierarchical genetic clustering plot of IBD segments detected in the ancient dataset.

Supplementary References

1. Arnay de la Rosa, M. M. & González Reimers, E. Una cueva sepulcral en la Cañada de la Angostura (Las Cañadas-Tenerife). in *Homenaje al profesor Dr. Telesforo Bravo* vol. 2 55–72 (Universidad de La Laguna, 1991).
2. Arnay de la Rosa, M. M., González-Reimers, E., Pou-Hernández, S., Marrero-Salas, E. & García-Ávila, C. Prehispanic (Guanches) mummies and natrium salts in burial caves of Las Cañadas del Teide (Tenerife). *Anthropol. Anz.* **74**, 143–153 (2017).
3. Galván, B. *et al.* Poblamiento prehistórico en la costa de Buenavista del Norte (Tenerife). El conjunto arqueológico Fuente-Arenas. *Investig. Arqueol.* 9–258 (1999).
4. Galván, B. *et al. Orígenes de Buenavista del Norte: de los primeros pobladores a los inicios de la colonización europea, Tenerife*. (Ayuntamiento de Buenavista del Norte-Cabildo Insular de Tenerife-Caja de Ahorros de Tenerife, 1999).
5. Sánchez-Cañadillas, E. *et al.* Dietary changes across time: Studying the indigenous period of La Gomera using δ13C and δ15N stable isotope analysis and radiocarbon dating. *Am. J. Phys. Anthropol.* **175**, 137–155 (2021).
6. Arnay de la Rosa, M. M., González Reimers, C. E. & Barandiarán Maestu, I. Investigaciones arqueológicas en el Parque Nacional del Teide. *Veleia Rev. Prehist. Hist. Antig. Arqueol. Filol. Clásicas* 1245–1256 (2007).
7. Ordóñez, A. C. *et al.* Use of Molecular Genetic Procedures for Sex Determination in 'Guanches' Children's Remains. in *Children, Spaces and Identity* (eds. Sánchez Romero, M., Alarcón García, E. & Aranda Jiménez, G.) 218–229 (Oxbow Books, 2015).
8. Alberto-Barroso, V. *et al. Guía del Patrimonio Arqueológico de Gran Canaria*. (Cabildo de Gran Canaria, 2012).
9. Cuenca Sanabria J., B. R. A. La práctica del infanticidio femenino como método de control de la natalidad entre los aborígenes canarios: las evidencias arqueológicas en Cendro, Telde, Gran Canaria. *El Mus. Canar.* **51**, 103–180 (1996).
10. Alberto-Barroso, V. *et al.* Postmortem Animal Interference in an Indigenous Burial (Lanzarote, Canary Islands). *Bioarchaeology Int.* **5**, (2021).
11. Morales, J., Rodríguez, A. & Henríquez, P. Agricultura y recolección vegetal en la arqueología prehispánica de las Islas Canarias (siglos III-XV d.C.): la contribución de los estudios carpológicos. in (2017).

12. Delgado-Darias, T. *La Historia en los Dientes. Una aproximación a la prehistoria de Gran Canaria desde la antropología dental*. (Cabildo de Gran Canaria, 2009).

13. Santana, J. Marcadores óseos de actividad física en la población aborigen de Gáldar (Siglos XI-XV). *Vegueta Anu. Fac. Geogr. E Hist.* **11**, 101–122 (2010).

14. Jiménez Gómez, M. de la C. La cueva sepulcral de la Cañada del Capricho (Las Cañadas del Teide, Tenerife). *Tabona Rev. Prehist. Arqueol.* 11–20 (1983).

15. Arnay de la Rosa, M. M. Estudio antropológico de los restos procedentes de la cueva sepulcral de la Cañada del Capricho. *Tabona Rev. Prehist. Arqueol.* 21–28 (1983).

16. Afonso Vargas, J. Á. Aplicación del análisis de fitolitos y otros microfósiles al estudio de yacimientos, materiales arqueológicos y edáficos de las Islas Canarias: los ejemplos de Las Cañadas del Teide (Tenerife), La Cerera (Arucas, Gran Canaria) y otras zonas de aplicación experimental. (Universidad de La Laguna, 2014).

17. Arnay de la Rosa, M. *et al.* Paleonutritional and paleodietary survey on prehistoric humans from Las Cañadas del Teide (Tenerife, Canary Islands) based on chemical and histological analysis of bone. *J. Archaeol. Sci.* **38**, 884–895 (2011).

18. Trujillo Mederos, A. & González Toledo, J. M. Tafonomía de Alta Montaña: aproximación multidisciplinar al estudio de restos parcialmente conservados. in *Actas de las IV Jornadas 'Prebendado Pacheco' de Investigación Histórica* 71–96 (2011).

19. Navarro Mederos, J. F. Excavaciones arqueológicas en El Hormiguero de Casablanca, Firgas (Gran Canaria). in 329–336 (1979).

20. Santana Cabrera, J., Mendoza Medina, F., Suárez Medina, I. & Moreno Benítez, M. Niños en la memoria: el depósito funerario del Barrranquillo del Cabezo. *El Mus. Canar.* 9–30 (2010).

21. Moreno Benítez, M. A., Mendoza Medina, F. & Suárez Medina, I. *Descripción bioantropológica y diagnóstico de conservación de los restos bioantropológicos de Huriamen para su certificación cronológica e histórica*. (2014).

22. Arnay de la Rosa, M. M. *et al. Estudios sobre el patrimonio arqueológico del Parque Nacional del Teide*. (2017).

23. Chil Naranjo, G. Expedición a Guayadeque (I). *El Mus. Canar.* **1**, 129–133 (1880).

24. Chil y Naranjo, G. *Estudios históricos, climatológicos y patológicos de las Islas Canarias*. (1876).

25. Fusté Ara, M. Algunas observaciones acerca de la antropología de las poblaciones prehistóricas y actual de Gran Canaria. *El Mus. Canar.* **65**, (1958).

26. Grau Bassas, V. Las cuevas de Guayadeque. *El Mus. Canar.* **1**, 65–69 (1880).

27. Archivo de El Museo Canario, Archivos y Correspondencia julio-diciembre 1956. (1956).

28. Pais Pais, J. & Álvarez Rodríguez, N. La Necrópolis aborigen del Huerto de Los Morales (Barranco de Fernando Porto, Garafía). in (2010).

29. Tibicena Arqueología y Patrimonio. *Proyecto de musealización del yacimiento arqueológico de La Fortaleza (T.M de Santa Lucía de Tirajana)*. (2010).

30. Santana, J. & Moreno, M. Movilidad y Adaptación Biomecánica: Una aproximación a la relación entre territorio y propiedades geométricas de la extremidad inferior en la población prehispánica de Gran Canaria. *Rev. Cienc. Tecnol. UPSE* **2–3**, (2014).

31. Velasco Vázquez, J., Martín Rodríguez, E., González Reimers, E., Arnay de la Rosa, M. & Betancor Rodríguez, A. Contribución de la bioantropología a la reconstrucción de los procesos productivos prehistóricos. Exostosis en el canal auditivo en la población prehispánica de Gran Canaria. *Trab. Prehist.* **58**, 109–125 (2001).

32. Santana Cabrera, J., Velasco Vázquez, J. & Rodríguez Rodríguez, A. del C. Patrón cotidiano de actividad física y organización social del trabajo en la Gran Canaria prehispánica (siglos XI-XV): la aportación de los marcadores óseos de actividad física. *Tabona Rev. Prehist. Arqueol.* 125–163 (2012).

33. Alberto-Barroso, V., Delgado-Darias, T., Moreno Benítez, M. & Velasco Vázquez, J. La dimensión temporal y el fenómeno sepulcral entre los antiguos canarios. *Zephyrus Rev. Prehist. Arqueol.* **84**, 139–160 (2019).

34. Socas Martín, D., Cámalich Massieu, M. D. & Thovar Melián, M. D. La cueva funeraria de la Montaña de Mina (San Bartolomé, Lanzarote) y su entorno. in *50 Aniversario del Instituto de Estudios Canarios1932-1982* vol. Tomo II 275–301 (1982).

35. Guillén, J. La cueva funeraria del Puente de La Calzada. *Bol. Patrim. Histórico* **2**, 18–19 (2004).

36. Ordóñez, A. C. *et al.* Genetic studies on the prehispanic population buried in Punta Azul cave (El Hierro, Canary Islands). *J. Archaeol. Sci.* **78**, 20–28 (2017).

37. Velasco Vázquez, J., Ruíz González, T. N. & Sánchez Perera, S. *El Lugar de los antepasados, La necrópolis bimbape de montaña la lajura, El Hierro*. (Cabildo Insular de El Hierro, 2005).

38. Velásco Vázquez, J. *El Lugar de los antepasados, La necrópolis bimbape de montaña la lajura, El Hierro*. (Cabildo insular de El Hierro, 2005).

39. Álvarez Delgado, J. *Excavaciones arqueológicas en Tenerife (Canarias).* vol. 14 (Ministerio de Educación Nacional, 1947).

40. Jiménez Gómez, M. de la C. *El Hierro y los Bimbaches*. (Centro de la Cultura Popular Canaria, 1993).

41. Arnay de la Rosa, M., Mas Pascual, A., González Reimers, E., Machado-Calvo, M. & Velasco Vázquez, J. Osteoporosis vertebral en la población prehispánica de El Hierro. *Anu. Inst. Estud. Canar.* **47**, 127–138 (2002).

42. Castaneyra-Ruiz, M., Trujillo-Mederos, A., Arnay-de-la-Rosa, M. & Gonzalez-Reimers, E. Osteoarthritis among the prehispanic population from La Gomera and El Hierro (Canary Islands): a comparative study. *Anthr. Anz* **72**, 347–58 (2015).

43. González-Reimers, E., Mas-Pascual, A., Arnay-De-La-Rosa, M., Velasco-Vásquez, J. & Jiménez-Gómez, M. C. Klippel-Feil syndrome in the prehispanic population of El Hierro (Canary Islands). *Ann. Rheum. Dis.* **60**, 174–174 (2001).

44. González-Reimers, E., Trujillo-Mederos, A., Ordóñez, A. C. & Arnay-da-la-Rosa, M. A case of calcaneal osteomyelitis from the prehispanic population of El Hierro (Canary Islands). *Int. J. Paleopathol.* **8**, 36–41 (2015).

45. Mas-Pascual, M. A. & González-Reimers, E. Spina bifida in the Prehispanic population of El Hierro (Canary Islands).

46. Arnay de la Rosa, M., Gonzalez-Reimers, E., Castilla-Garcia, A. & Santolaria-Fernandez, F. Radiopaque transverse lines (Harris lines) in the prehispanic population of El Hierro (Canary Islands). *Anthr. Anz* **52**, 53–7 (1994).

47. Arnay de la Rosa, M. *et al.* Paleodietary analysis of the prehistoric population of the Canary Islands inferred from stable isotopes (carbon, nitrogen and hydrogen) in bone collagen. *J. Archaeol. Sci.* **37**, 1490–1501 (2010).

48. Velasco Vázquez, J., Arnay de la Rosa, M. M., González Reimers, C. E. & Chinea Díaz, D. J. Estudio de las líneas de Harris de la población inhumada en la iglesia de la Concepción (Santa Cruz de Tenerife). in *XII Coloquio de Historia Canario-Americana: (1996)* vol. 1 493–506 (1998).

49. Ordóñez, A. C. *et al.* Genetic sexing to determine the optimal discriminant functions for the analysis of archaeological remains from El Hierro (Canary Islands). *J. Archaeol. Sci.* **40**, 4411–4419 (2013).

50. Emilio González-Reimers, M. A.-R. Relationships between bending and torsional strengths of the tibia and simple anthropometric variables among the prehispanic population of El Hierro (Canary Islands). *Eur J Anat* **18**, 8–15 (2014).

51. Gonzalez-Reimers, E., Castaneyra-Ruiz, M., Trujillo-Mederos, A. & Arnay-de-la-Rosa, M. Differences in tibial shape among the Prehispanic inhabitants from Gomera, Punta Azul (El Hierro), and Gran Canaria (Canary Islands). *Anthr. Anz* **74**, 309–319 (2017).

52. Cuscoy, L. D. *Tres cuevas sepulcrales guanches (Tenerife)*. (1965).

53. Arnay-de-la-Rosa, M., Gonzalez-Reimers, E., Pou-Hernandez, S., Marrero-Salas, E. & Garcia-Avila, C. Prehispanic (Guanches) mummies and natrium salts in burial caves of Las Canadas del Teide (Tenerife). *Anthr. Anz* **74**, 143–153 (2017).

54. Fregel, R. *et al.* Ancient genomes from North Africa evidence prehistoric migrations to the Maghreb from both the Levant and Europe. *Proc. Natl. Acad. Sci.* **115**, 6774–6779 (2018).

55. Schubert, M., Lindgreen, S. & Orlando, L. AdapterRemoval v2: rapid adapter trimming, identification, and read merging. *BMC Res. Notes* **9**, 88 (2016).

56. Li, H. & Durbin, R. Fast and accurate short read alignment with Burrows–Wheeler transform. *Bioinformatics* **25**, 1754–1760 (2009).

57. Jun, G., Wing, M. K., Abecasis, G. R. & Kang, H. M. An efficient and scalable analysis framework for variant extraction and refinement from population-scale DNA sequence data. *Genome Res.* **25**, 918–925 (2015).

58. Li, H. *et al.* The Sequence Alignment/Map format and SAMtools. *Bioinformatics* **25**, 2078–2079 (2009).

59. R Core Team. R: a language and environment for statistical computing. *Found. Stat. Comput.* (2020).

60. Wickham, H. *ggplot2: Elegant Graphics for Data Analysis*. (Springer, 2016).

61. Ginolhac, A., Rasmussen, M., Gilbert, M. T. P., Willerslev, E. & Orlando, L. mapDamage: testing for damage patterns in ancient DNA sequences. *Bioinformatics* **27**, 2153–2155 (2011).

62. Jónsson, H., Ginolhac, A., Schubert, M., Johnson, P. L. F. & Orlando, L. mapDamage2.0: fast approximate Bayesian estimates of ancient DNA damage parameters. *Bioinformatics* **29**, 1682–1684 (2013).

63. Fu, Q. *et al.* DNA analysis of an early modern human from Tianyuan Cave, China. *Proc. Natl. Acad. Sci.* **110**, 2223–2227 (2013).

64. Renaud, G., Slon, V., Duggan, A. T. & Kelso, J. Schmutzi: estimation of contamination and endogenous mitochondrial consensus calling for ancient DNA. *Genome Biol.* **16**, 224 (2015).

65. Korneliussen, T. S., Albrechtsen, A. & Nielsen, R. ANGSD: Analysis of Next Generation Sequencing Data. *BMC Bioinformatics* **15**, 356 (2014).

66. Fregel, R. *et al.* Mitogenomes illuminate the origin and migration patterns of the indigenous people of the Canary Islands. *PLoS ONE* **14**, e0209125 (2019).

67. Skoglund, P. *et al.* Genetic evidence for two founding populations of the Americas. *Nature* **525**, 104–108 (2015).

68. Rodríguez-Varela, R. *et al.* Genomic Analyses of Pre-European Conquest Human Remains from the Canary Islands Reveal Close Affinity to Modern North Africans. *Curr. Biol.* **27**, 3396-3402.e5 (2017).

69. Poznik, G. D. *et al.* Punctuated bursts in human male demography inferred from 1,244 worldwide Y-chromosome sequences. *Nat. Genet.* **48**, 593–599 (2016).

70. Martiniano, R., De Sanctis, B., Hallast, P. & Durbin, R. Placing Ancient DNA Sequences into Reference Phylogenies. *Mol. Biol. Evol.* **39**, msac017 (2022).

71. Yu, G., Smith, D. K., Zhu, H., Guan, Y. & Lam, T. T.-Y. ggtree: an R package for visualization and annotation of phylogenetic trees with their covariates and other associated data. *Methods Ecol. Evol.* **8**, 28–36 (2017).

72. Alberto-Barroso, V. *et al.* Perinatal burials at pre-Hispanic noncemetery sites in Gran Canaria: Tophet, infanticide, or natural mortality? *Int. J. Osteoarchaeol.* **32**, 100–110 (2022).

73. Fregel, R. *et al.* Demographic history of Canary Islands male gene-pool: replacement of native lineages by European. *BMC Evol. Biol.* **9**, 181 (2009).

74. Solé-Morata, N. *et al.* Whole Y-chromosome sequences reveal an extremely recent origin of the most common North African paternal lineage E-M183 (M81). *Sci. Rep.* **7**, 15941 (2017).

75. Flores, C., Maca-Meyer, N., Pérez-Pérez, J. A., Hernández, M. & Cabrera, V. M. Y-Chromosome Differentiation in Northwest Africa. *Hum. Biol.* **73**, 513–524 (2001).

76. Bosch, E. *et al.* High-resolution analysis of human Y-chromosome variation shows a sharp discontinuity and limited gene flow between northwestern Africa and the Iberian Peninsula. *Am. J. Hum. Genet.* **68**, 1019–1029 (2001).

77. Myres, N. M. *et al.* A major Y-chromosome haplogroup R1b Holocene era founder effect in Central and Western Europe. *Eur. J. Hum. Genet.* **19**, 95–101 (2011).
78. Kivisild, T. The study of human Y chromosome variation through ancient DNA. *Hum. Genet.* **136**, 529–546 (2017).
79. Ben-Ncer, A., Bokbot, Y., Amani, F. & Ouachi, M. Study of the Chalcolithic Burial 2 and 3 of Ifri n'Amr ou Moussa (Morocco). in *Around the Petit-Chasseur Site in Sion (Valais, Switzerland) and New Approaches to the Bell Beaker Culture.* (ed. Besse, M.) 251–258 (Archaeopress Archaeology, 2014).
80. Bokbot, Y. & Ben-Ncer, A. Découvertes campaniformes récentes dans les plateaux de Zemmour (Maroc). in *Bell Beaker in everyday life. Proceedings of the 10th "Meeting Archéologie et Gobelets" (Florence – Siena – Villanuova sul Clisi, May 12-15, 2006)* (eds. Baioni, M. et al.) 327–330 (Museo Fiorentino di Preistoria «Paolo Graziosi», 2008).
81. Mendez, F. L. *et al.* Increased Resolution of Y Chromosome Haplogroup T Defines Relationships among Populations of the Near East, Europe, and Africa. *Hum. Biol.* **83**, 39–53 (2011).
82. Haak, W. *et al.* Massive migration from the steppe was a source for Indo-European languages in Europe. *Nature* **522**, 207–211 (2015).
83. Cruciani, F. *et al.* Tracing Past Human Male Movements in Northern/Eastern Africa and Western Eurasia: New Clues from Y-Chromosomal Haplogroups E-M78 and J-M12. *Mol. Biol. Evol.* **24**, 1300–1311 (2007).
84. Trombetta, B. *et al.* Phylogeographic Refinement and Large Scale Genotyping of Human Y Chromosome Haplogroup E Provide New Insights into the Dispersal of Early Pastoralists in the African Continent. *Genome Biol. Evol.* **7**, 1940–1950 (2015).
85. van de Loosdrecht, M. *et al.* Pleistocene North African genomes link Near Eastern and sub-Saharan African human populations. *Science* **360**, 548–552 (2018).
86. Cann, H. M. *et al.* A Human Genome Diversity Cell Line Panel. *Science* **296**, 261–262 (2002).
87. Lazaridis, I. *et al.* Genomic insights into the origin of farming in the ancient Near East. *Nature* **536**, 419–424 (2016).
88. Olalde, I. *et al.* The genomic history of the Iberian Peninsula over the past 8000 years. *Science* **363**, 1230–1234 (2019).
89. Valdiosera, C. *et al.* Four millennia of Iberian biomolecular prehistory illustrate the impact of prehistoric migrations at the far end of Eurasia. *Proc. Natl. Acad. Sci.* **115**, 3428–3433 (2018).

90. Hofmanová, Z. *et al.* Early farmers from across Europe directly descended from Neolithic Aegeans. *Proc. Natl. Acad. Sci.* **113**, 6886–6891 (2016).

91. Cassidy, L. M. *et al.* Neolithic and Bronze Age migration to Ireland and establishment of the insular Atlantic genome. *Proc. Natl. Acad. Sci.* **113**, 368–373 (2016).

92. Gallego-Llorente, M. *et al.* The genetics of an early Neolithic pastoralist from the Zagros, Iran. *Sci. Rep.* **6**, 31326 (2016).

93. Schuenemann, V. J. *et al.* Ancient Egyptian mummy genomes suggest an increase of Sub-Saharan African ancestry in post-Roman periods. *Nat. Commun.* **8**, 15694 (2017).

94. Schlebusch, C. M. *et al.* Southern African ancient genomes estimate modern human divergence to 350,000 to 260,000 years ago. *Science* **358**, 652–655 (2017).

95. Skoglund, P. *et al.* Reconstructing Prehistoric African Population Structure. *Cell* **171**, 59-71.e21 (2017).

96. Zalloua, P. *et al.* Ancient DNA of Phoenician remains indicates discontinuity in the settlement history of Ibiza. *Sci. Rep.* **8**, 17567 (2018).

97. Antonio, M. L. *et al.* Ancient Rome: A genetic crossroads of Europe and the Mediterranean. *Science* **366**, 708–714 (2019).

98. Wang, C. *et al.* Ancestry estimation and control of population stratification for sequence-based association studies. *Nat. Genet.* **46**, 409–415 (2014).

99. Wang, C., Zhan, X., Liang, L., Abecasis, G. R. & Lin, X. Improved Ancestry Estimation for both Genotyping and Sequencing Data using Projection Procrustes Analysis and Genotype Imputation. *Am. J. Hum. Genet.* **96**, 926–937 (2015).

100. Price, A. L. *et al.* Principal components analysis corrects for stratification in genome-wide association studies. *Nat. Genet.* **38**, 904–909 (2006).

101. Henn, B. M. *et al.* Genomic Ancestry of North Africans Supports Back-to-Africa Migrations. *PLoS Genet.* **8**, e1002397 (2012).

102. Fregel, R., Ordóñez, A. C. & Serrano, J. G. The demography of the Canary Islands from a genetic perspective. *Hum. Mol. Genet.* **30**, R64–R71 (2021).

103. Marcus, J. H. *et al.* Genetic history from the Middle Neolithic to present on the Mediterranean island of Sardinia. *Nat. Commun.* **11**, 939 (2020).

104. Moots, H. M. *et al.* A Genetic History of Continuity and Mobility in the Iron Age Central Mediterranean. Preprint at https://doi.org/10.1101/2022.03.13.483276 (2022).

105. Purcell, S. *et al.* PLINK: A Tool Set for Whole-Genome Association and Population-Based Linkage Analyses. *Am. J. Hum. Genet.* **81**, 559–575 (2007).

106. Alexander, D. H., Novembre, J. & Lange, K. Fast model-based estimation of ancestry in unrelated individuals. *Genome Res.* **19**, 1655–1664 (2009).

107. Velasco Vázquez, J. *et al.* Poblamiento, colonización y primera historia de Canarias: El C14 como paradigma. *Anu. Estud. Atlánticos* **AEA**, 1–24 (2020).

108. Acosta Armas, J. Notas sobre la aspiración en los guanchismos. *Rev. Filol. Univ. Laguna* 9–49 (2017).

109. Alberto-Barroso, V. *et al.* Sobre el tiempo de los majos. Nuevas fechas para el conocimiento del poblamiento aborigen de Lanzarote. *Anu. Estud. Atlánticos* 1–0 (2022).

110. Arco Aguilar, M. C. De nuevo sobre el descubrimiento y colonización antiguos de Canarias. Reflexiones sobre aspectos teóricos y datos empíricos. *Anu. Estud. Atlánticos* (2021).

111. Arco Aguilar, M. C., Arco Aguilar, M. M., Benito Mateo, C. & Rosario Adrian, M. C. *Un taller romano de púrpura en los limites de la Ecumene: Lobos 1*. (Cabildo de Tenerife, 2017).

112. Atoche Peña, P. & Ramírez Rodríguez, M. Á. El yacimiento de Buenavista, un asentamiento fenicio púnico en Lanzarote, Islas Canarias (circa 960-360 a.n.e.). in *La vie, la mort et la religión dans l'univers phénicien et punique: Actes du VIIème Congrès International des Études Phéniciennes et Puniques* vol. 1 365–380 (Institut National du Patrimoine, 2019).

113. Atoche Peña, P. Consideraciones en relación con la colonización protohistórica de las Islas Canarias. *Anu. Estud. Atlánticos* 521–564 (2013).

114. Martín Culebras, J. & Atoche Peña, P. Canarias en la expansión fenicio-púnica por el África Atlántica. in *II Congreso de Arqueología Peninsular: Zamora, del 24 al 27 de septiembre de 1996* vol. 3 485–500 (Fundación Rei Afonso Henriques, 1999).

115. Martín Ruiz, J. A. La colonización fenicia en las Islas Canarias. Una cuestión a debate. *Albahri Entre Oriente Occidente Rev. Independiente Estud. Históricos* 9–42 (2015).

116. Méndez Rodríguez, D. M. *Momias, xaxos y mirlados: las narraciones sobre el embalsamiento de los aborígenes de las Islas Canarias (1482-1803)*. (Instituto de Estudios Canarios, 2014).

117. Mora Aguiar, I. Influencias e innovaciones gráficas en la creación del alfabeto líbico oriental (Túnez y Argelia). *Vegueta* 24–0 (2017).

118. Mora Aguiar, I. La dispersión de la escritura líbico-bereber desde Numidia hasta Canarias. in *Aprender la escritura, olvidar la escritura* (eds. Moncunill Martí, N. & Ramírez-Sánchez, M.) 39–64 (Universidad del País Vasco/Euskal Herriko Unibertsitatea, 2021).

119. Mora Aguiar, I. El origen de la escritura líbico-bereber: dataciones e hipótesis. *Tabona Rev. Prehist. Arqueol.* 11–28 (2015).

120. Springer Bunk, R. El alfabeto líbico-bereber canario: la distribución geográfica de los signos en el Norte de África y Sáhara. *Vegueta* 759–772 (2019).

121. Atoche Peña, P., Ramírez, M., Rodríguez-Martín, C., Rodríguez, M. & Perez, S. De antropología, ritos y creencias en la Protohistoria de Lanzarote (Islas Canarias). in *Mummies and Science. World Mummies Research. Proceedings of the VI World Congress on Mummy Studies* 165–180 (Academia Canaria de la Historia, 2008).

122. Springer Bunk, R. La escritura líbico-bereber de las Islas Canarias: ¿uno o varios alfabetos? *Tabona Rev. Prehist. Arqueol.* (2017).

123. Atoche Peña, P. & Ramírez Rodríguez, M. Á. Manifestaciones de la religiosidad mediterránea en la Protohistoria canaria ('circa' s. X a.n.e. al s. XV d.n.e.). *Gerión* **26**, 183–202 (2008).

124. Harney, É., Patterson, N., Reich, D. & Wakeley, J. Assessing the performance of qpAdm: a statistical tool for studying population admixture. *Genetics* **217**, iyaa045 (2021).

125. Martiniano, R. *et al.* Genomic signals of migration and continuity in Britain before the Anglo-Saxons. *Nat. Commun.* **7**, 10326 (2016).

126. Monroy Kuhn, J. M., Jakobsson, M. & Gunther, T. Estimating genetic kin relationships in prehistoric populations. *Plos One* **13**, e0195491 (2018).

127. Skoglund, P. & Reich, D. A genomic view of the peopling of the Americas. *Curr. Opin. Genet. Dev.* **41**, 27–35 (2016).

128. Ringbauer, H., Novembre, J. & Steinrücken, M. Parental relatedness through time revealed by runs of homozygosity in ancient DNA. *Nat. Commun.* **12**, 5425 (2021).

129. Guillen-Guio, B. *et al.* Genomic Analyses of Human European Diversity at the Southwestern Edge: Isolation, African Influence and Disease Associations in the Canary Islands. *Mol. Biol. Evol.* **35**, 3010–3026 (2018).

130. Tournebize, R., Chu, G. & Moorjani, P. Reconstructing the history of founder events using genome-wide patterns of allele sharing across individuals. *PLoS Genet.* **18**, e1010243 (2022).

131. Anguita-Ruiz, A., Aguilera, C. M. & Gil, Á. Genetics of Lactose Intolerance: An Updated Review and Online Interactive World Maps of Phenotype and Genotype Frequencies. *Nutrients* **12**, 2689 (2020).

132. Guillen-Guio, B. *et al.* Admixture mapping of asthma in southwestern Europeans with North African ancestry influences. *Am. J. Physiol. - Lung Cell. Mol. Physiol.* **318**, L965–L975 (2020).

133. Flores, C. *et al.* The Origin of the Canary Island Aborigines and Their Contribution to the Modern Population: A Molecular Genetics Perspective. *Curr. Anthropol.* **42**, 749–755 (2001).

134. Flores, C. *et al.* A Predominant European Ancestry of Paternal Lineages from Canary Islanders. *Ann. Hum. Genet.* **67**, 138–152 (2003).

135. Alvarez, L. *et al.* Y-chromosome variation in South Iberia: Insights into the North African contribution. *Am. J. Hum. Biol.* **21**, 407–409 (2009).

136. Flores, C. *et al.* Reduced genetic structure of the Iberian peninsula revealed by Y-chromosome analysis: implications for population demography. *Eur. J. Hum. Genet.* **12**, 855–863 (2004).

137. Long, J. C. The genetic structure of admixed populations. *Genetics* **127**, 417–428 (1991).

138. Arnay de la Rosa, M., Ordóñez, A. C. & Pérez Alvarez, A. R. Archaeological evidence of the movement of people and goods in the Canary Islands in the 18th century. Slaves and tobacco in the archaeological site of La Iglesia de Nuestra Señora de La Concepción de Santa Cruz de Tenerife. *Vegueta Anu. Fac. Geogr. E Hist.* **15**, 37–64 (2015).

139. Rubinacci, S., Ribeiro, D. M., Hofmeister, R. J. & Delaneau, O. Efficient phasing and imputation of low-coverage sequencing data using large reference panels. *Nat. Genet.* **53**, 120–126 (2021).

140. Allentoft, M. E. *et al.* Population Genomics of Stone Age Eurasia. Preprint at https://doi.org/10.1101/2022.05.04.490594 (2022).

141. Browning, B. L. & Browning, S. R. Improving the Accuracy and Efficiency of Identity-by-Descent Detection in Population Data. *Genetics* **194**, 459–471 (2013).

142. Villalba-Mouco, V. *et al.* Genomic transformation and social organization during the Copper Age–Bronze Age transition in southern Iberia. *Sci. Adv.* **7**, eabi7038 (2021).

143. Posth, C. *et al.* The origin and legacy of the Etruscans through a 2000-year archeogenomic time transect. *Sci. Adv.* **7**, eabi7673 (2021).

144. Patterson, N. *et al.* Large-scale migration into Britain during the Middle to Late Bronze Age. *Nature* **601**, 588–594 (2022).

145. Fernandes, D. M. *et al.* The spread of steppe and Iranian-related ancestry in the islands of the western Mediterranean. *Nat. Ecol. Evol.* **4**, 334–345 (2020).

146. Clemente, F. *et al.* The genomic history of the Aegean palatial civilizations. *Cell* **184**, 2565-2586.e21 (2021).

147. Csardi, G. & Nepusz, T. The igraph software package for complex network research. (2006).

148. Traag, V. A., Waltman, L. & van Eck, N. J. From Louvain to Leiden: guaranteeing well-connected communities. *Sci. Rep.* **9**, 5233 (2019).
