# Supplementary material for: The genomic history of the indigenous people of the Canary Islands
Source: Nat Commun. 2023 Aug 15;14:4641. doi: 10.1038/s41467-023-40198-w (PMC10427657; doi:10.1038/s41467-023-40198-w)
Supplement: Supplementary file 3 — Description of Additional Supplementary Files [file 41467_2023_40198_MOESM3_ESM.pdf]

Description of Additional Supplementary Files

File Name: Supplementary Data 1
Description: Summary of results for all ancient samples analyzed in this study.

File Name: Supplementary Data 2
Description: Y-chromosome derived marker assignation for each method/database used.

File Name: Supplementary Data 3
Description: qpAdm admixture modelling results.

File Name: Supplementary Data 4
Description: qpAdm admixture results for the CIP when considering Romans and Punics as sources of steppe ancestry.

File Name: Supplementary Data 5
Description: qpAdm admixture results for regions and island populations.

File Name: Supplementary Data 6
Description: qpAdm admixture results for individuals.

File Name: Supplementary Data 7
Description: Heterozygosity values obtained for all the populations considered.

File Name: Supplementary Data 8
Description: Runs of Homozygosity observed for the CIP using hapROH.

File Name: Supplementary Data 9
Description: Effective population size (Ne) results when correcting for sampling bias.

File Name: Supplementary Data 10
Description: Bottleneck events inferred using ASCEND.
