# Supplementary material for: The genomic history of the indigenous people of the Canary Islands
Source: Nat Commun. 2023 Aug 15;14:4641. doi: 10.1038/s41467-023-40198-w (PMC10427657; doi:10.1038/s41467-023-40198-w)
Supplement: Supplementary file 5 — Reporting Summary [file 41467_2023_40198_MOESM5_ESM.pdf]

nature portfolio

Corresponding author(s): Rosa Fregel
Last updated by author(s): Jun 30, 2023

Reporting Summary

Nature Portfolio wishes to improve the reproducibility of the work that we publish. This form provides structure for consistency and transparency in reporting. For further information on Nature Portfolio policies, see our Editorial Policies and the Editorial Policy Checklist.

Statistics

For all statistical analyses, confirm that the following items are present in the figure legend, table legend, main text, or Methods section.

n/a	Confirmed	
☐	☒	The exact sample size (*n*) for each experimental group/condition, given as a discrete number and unit of measurement
☐	☒	A statement on whether measurements were taken from distinct samples or whether the same sample was measured repeatedly
☒	☐	The statistical test(s) used AND whether they are one- or two-sided<br>*Only common tests should be described solely by name; describe more complex techniques in the Methods section.*
☒	☐	A description of all covariates tested
☐	☒	A description of any assumptions or corrections, such as tests of normality and adjustment for multiple comparisons
☐	☒	A full description of the statistical parameters including central tendency (e.g. means) or other basic estimates (e.g. regression coefficient) AND variation (e.g. standard deviation) or associated estimates of uncertainty (e.g. confidence intervals)
☒	☐	For null hypothesis testing, the test statistic (e.g. *F*, *t*, *r*) with confidence intervals, effect sizes, degrees of freedom and *P* value noted<br>*Give P values as exact values whenever suitable.*
☒	☐	For Bayesian analysis, information on the choice of priors and Markov chain Monte Carlo settings
☒	☐	For hierarchical and complex designs, identification of the appropriate level for tests and full reporting of outcomes
☒	☐	Estimates of effect sizes (e.g. Cohen's *d*, Pearson's *r*), indicating how they were calculated

Our web collection on statistics for biologists contains articles on many of the points above.

Software and code

Policy information about availability of computer code

Data collection	Specific software was not used for data acquisition.
Data analysis	OxCal version 4.4; AdapterRemoval v.2.1; BWA v.0.7.12; bamUtil v.1.0.14; samtools v.0.1.19; mapDamage v.2.02; ContamMix v.1.0-10; Schmutzi v1.5.4; pathPhynder v1.a; smartpca v.181602; PLINK v.1.90; ADMIXTURE v.1.3.0; ADMIXTOOLS v.7.0; popstats v.1.0; hapROH v.0.54; GLIMPSE v.10.1; IBDseq v.r1206.; WLSAdmix; READ v.1.0; ASCEND v.8.0 . We used the R language versions 3.6 and 4.1 and the R packages igraph v.1.3.5; and leidenAlg v.1.0.5.

For manuscripts utilizing custom algorithms or software that are central to the research but not yet described in published literature, software must be made available to editors and reviewers. We strongly encourage code deposition in a community repository (e.g. GitHub). See the Nature Portfolio guidelines for submitting code & software for further information.

Data

Policy information about availability of data

All manuscripts must include a data availability statement. This statement should provide the following information, where applicable:

- Accession codes, unique identifiers, or web links for publicly available datasets
- A description of any restrictions on data availability
- For clinical datasets or third party data, please ensure that the statement adheres to our policy

The sequence data generated in this study have been deposited in the European Nucleotide Archive (ENA) database under accession number PRJEB61655 (https://

www.ebi.ac.uk/ena/browser/view/PRJEB61655). Additional Canarian individuals included in this study were obtained from their ENA accession no. PRJEB86458 (https://www.ebi.ac.uk/ena/browser/view/PRJEB86458) and PRJEB46005 (https://www.ebi.ac.uk/ena/browser/view/PRJEB46005). The human reference sequence build 37 (GRCh37/hg19; https://www.ncbi.nlm.nih.gov/assembly/GCF_000001405.13/) and the Revised Cambridge Reference Sequence (rCRS; NC_012920 (https://www.ncbi.nlm.nih.gov/nuccore/251831106) were downloaded from the National Center for Biotechnology Information (NCBI). 1000 Genomes Project phase 372 used as reference dataset for imputation is available through the ENA accession number PRJEB31736 (https://www.ebi.ac.uk/ena/browser/view/PRJEB31736). The Human Genome Diversity Project (HGDP) genotyped with the MEGA array is available at https://bustamantelab.stanford.edu, and the ISOGG Y-DNA Haplogroup tree 2019-2020 database (v.15.73) at https://isogg.org/tree/. The Allen Ancient DNA Resource dataset (AADR) version 42.4 is publicly available at https://reich.hms.harvard.edu/ancient-genome-diversity-project. The remaining ancient genomic data not included in the AADR were collected from the ENA through their accession no.: Neolithic genomes from Iran (PRJEB13189: https://www.ebi.ac.uk/ena/browser/view/PRJEB13189); ancient Mediterranean genomes (PRJEB35980: https://www.ebi.ac.uk/ena/browser/view/PRJEB35980); Bronze Age individuals from Greece (PRJEB37782: https://www.ebi.ac.uk/ena/browser/view/PRJEB37782); ancient Sardinians (PRJEB35094: https://www.ebi.ac.uk/ena/browser/view/PRJEB35094); Etruscans (PRJEB42866: https://www.ebi.ac.uk/ena/browser/view/PRJEB42866); and ancient genomes from the Iberian Peninsula (PRJEB46907: https://www.ebi.ac.uk/ena/browser/view/PRJEB46907); and from Great Britain (PRJEB47891: https://www.ebi.ac.uk/ena/browser/view/PRJEB47891). Array data from present-day Canary Islanders was obtained from 39 (https://www.iter.es/wp-content/uploads/2018/09/AffyCEU1_data_from_Canary_Islanders_MBE-Guillen-Guio-et-al.2018.zip).

Research involving human participants, their data, or biological material

Policy information about studies with human participants or human data. See also policy information about sex, gender (identity/presentation), and sexual orientation and race, ethnicity and racism.

Reporting on sex and gender	Not applicable.
Reporting on race, ethnicity, or other socially relevant groupings	Not applicable.
Population characteristics	Not applicable.
Recruitment	Not applicable.
Ethics oversight	Not applicable.

Note that full information on the approval of the study protocol must also be provided in the manuscript.

Field-specific reporting

Please select the one below that is the best fit for your research. If you are not sure, read the appropriate sections before making your selection.

☐ Life sciences ☐ Behavioural & social sciences ☒ Ecological, evolutionary & environmental sciences

For a reference copy of the document with all sections, see nature.com/documents/nr-reporting-summary-flat.pdf

Ecological, evolutionary & environmental sciences study design

All studies must disclose on these points even when the disclosure is negative.

Study description	In this study, we generated 9 medium to low-coverage genomes (5.82X – 0.36X) by shotgun sequencing and genome-wide data from another 31 individuals by in-solution capture from the Canarian indigenous population. The samples are distributed over twenty-three archaeological sites from the seven main islands, comprising a time transect of c. 1,300 years from the Canarian indigenous history, from the 3rd to the 16th century CE. With this dataset, we performed ancestry inference of the Canary Islands indigenous population, determined their population structure and explored their signatures of isolation. We performed the analyses hierarchically, first by analyzing samples from the whole archipelago and then all the insular populations independently. When possible (e.g. heterozygosity estimation), we also analyzed samples grouped by archaeological site.
Research sample	We generated genome-wide data from 40 individuals from the indigenous population of the Canary Islands, dated between the 3rd – 16th centuries CE. Previous paleogenomic studies were focused on individuals from the central islands of Gran Canaria and Tenerife. However, available mitochondrial data suggested that the genetic composition of the archipelago was heterogenous. For that reason, we selected individuals from all main islands: El Hierro (n = 4), La Palma (n = 3), La Gomera (n = 4), Tenerife (n = 8), Gran Canaria (n = 17), Fuerteventura (n = 2) y Lanzarote (n = 2). Sample selection was performed first based on the availability of samples in the local museums and then based on their conservation status both macroscopically and, once sequenced, based on their endogenous DNA content. We also included all published data from this population: five decontextualized individuals from the islands of Tenerife and Gran Canaria, ranging from the 7th to the 15th centuries (Rodríguez-Varela et al. 2017), and four from the Cendro site in Gran Canaria dated around the 12th century (Alberto-Barroso et al. 2022).
Sampling strategy	The sampling strategy was focused on obtaining well-conserved bone or teeth samples from at least five individuals from each island. However, as it is the case for most paleogenomic studies, we were limited by the availability of human remains from certain regions and by poor DNA conservation in some individuals. Data collection was performed by geneticists and archaeologists by writing down all information associated to the sample in an excel spreadsheet.
Data collection	Human remains were selected by our team of archaeologists and anthropologists with the aim of selecting the best-conserved

Data collection	human remains from the larger number of archaeological sites. We also focused, whenever possible, on human remains belonging to known archaeological contexts rather than decontextualized individuals from museum collections.
Timing and spatial scale	All samples were collected at the beginning of the study. Sample collection was performed in the insular museums and local universities between 1/8/2016 and 1/14/2016.<br>If data was not already available, the time period of each individual was determined by radiocarbon dating.
Data exclusions	Some analyses were performed on those samples with a higher genome coverage. This information is included in the Supplementary File.
Reproducibility	This project involves the analysis of ancient DNA from selected archaeological material. For guaranteeing the authenticity of the results, we followed strict protocols to avoid contamination with modern DNA, including the extraction and manipulation of ancient DNA in a clean dedicated laboratory, as well as a proper analysis of the genomic data to avoid artefactual results. As sample sizes for some islands are small, we tested for sampling bias for calculating heterozygosity, effective population size, bottleneck effects and admixture estimations. We also tested for the effect of genome coverage for estimating bottleneck effects and heterozygosity values.
Randomization	Individuals were grouped based on their island and archaeological site location if available. No more covariates were considered.
Blinding	Blinding was not of particular relevance for this study giving that we work with archaeological human remains. However, all exploratory analyses, including PCA and unsupervised clustering analyses, allow to characterize the genetic composition of our sample without having into consideration known geographical ascriptions.

Did the study involve field work? ☐ Yes ☒ No

Reporting for specific materials, systems and methods

We require information from authors about some types of materials, experimental systems and methods used in many studies. Here, indicate whether each material, system or method listed is relevant to your study. If you are not sure if a list item applies to your research, read the appropriate section before selecting a response.

Materials & experimental systems

n/a	Involved in the study
☒	☐ Antibodies
☒	☐ Eukaryotic cell lines
☐	☒ Palaeontology and archaeology
☒	☐ Animals and other organisms
☒	☐ Clinical data
☒	☐ Dual use research of concern
☒	☐ Plants

Methods

n/a	Involved in the study
☒	☐ ChIP-seq
☒	☐ Flow cytometry
☒	☐ MRI-based neuroimaging

Palaeontology and Archaeology

Specimen provenance	Samples were taken from human remains conserved at the insular museums of Gran Canaria (El Museo Canario), La Palma (Museo Arqueológico Benehaorita) and La Gomera (Museo Arqueológico de La Gomera), as well as, from archaeological remains that have been under study at the universities of La Laguna (Tenerife) and Las Palmas de Gran Canaria (Gran Canaria), and by the company Tibicena Arqueología y Patrimonio Ltd. (Gran Canaria).
Specimen deposition	Specimens used in this study are deposited in the institutions mentioned in the previous section. All the museum IDs have been recorded in order to allow future analyses on the same human remains.
Dating methods	Bone and tooth collagen were sampled from the same specimen used for ancient DNA analysis, but enamel was also employed in samples where tooth collagen was exhausted in the DNA extraction. The 14C dates were then calibrated with the internationally agreed IntCal20 atmospheric calibration curve using the OxCal online software version 4.4 (https://c14.arch.ox.ac.uk/oxcal.html) (Ramsey, 2017). The two-sigma probability interval (95.4%), recommended by Millard (2014), was used when discussing the 14C measurements.

☒ Tick this box to confirm that the raw and calibrated dates are available in the paper or in Supplementary Information.

Ethics oversight	Authorizations for accessing the archaeological material have been granted by Dirección General de Patrimonio Cultural del Gobierno de Canarias (reference 51/2020-0717115014).<br>The study of ancient populations involves following a series of requirements that ensure an ethical treatment of the archaeological remains and a proper heritage conservation (Prendergast and Sawchuk, 2018). To accomplish this, we only took the archaeological material strictly necessary to meet the objectives of this study and, whenever possible, we used less-destructive sampling methods such as the use of teeth and/or small bones, instead of the cutting of small fragments from long bones, such as tibiae or femurs.

Note that full information on the approval of the study protocol must also be provided in the manuscript.
